# A detailed picture of a protein–carbohydrate hydrogen-bonding network revealed by NMR and MD simulations

**DOI:** 10.1093/glycob/cwaa081

**Published:** 2020-09-08

**Authors:** Gustav Nestor, Alessandro Ruda, Taigh Anderson, Stefan Oscarson, Göran Widmalm, Angela M Gronenborn

**Affiliations:** 1 Department of Structural Biology, University of Pittsburgh School of Medicine,1051 BST3, 3501 Fifth Ave, Pittsburgh, PA 15261, USA; 2 Department of Molecular Sciences, Swedish University of Agricultural Sciences, P.O. Box 7015, SE-750 07, Uppsala, Sweden; 3 Department of Organic Chemistry, Stockholm University, Svante Arrhenius väg 16C, Stockholm, Sweden; 4 Centre for Synthesis and Chemical Biology, University College Dublin, Belfield, Dublin 4, Ireland

**Keywords:** carbohydrates, cyanovirin-N, hydrogen bonds, hydroxyls, NMR spectroscopy

## Abstract

Cyanovirin-N (CV-N) is a cyanobacterial lectin with antiviral activity towards HIV and several other viruses. Here, we identify mannoside hydroxyl protons that are hydrogen bonded to the protein backbone of the CV-N domain B binding site, using NMR spectroscopy. For the two carbohydrate ligands Manα(1→2)Manα*O*Me and Manα(1→2) Manα(1→6)Manα*O*Me five hydroxyl protons are involved in hydrogen-bonding networks. Comparison with previous crystallographic results revealed that four of these hydroxyl protons donate hydrogen bonds to protein backbone carbonyl oxygens in solution and in the crystal. Hydrogen bonds were not detected between the side chains of Glu41 and Arg76 with sugar hydroxyls, as previously proposed for CV-N binding of mannosides. Molecular dynamics simulations of the CV-N/Manα(1→2)Manα(1→6)Manα*O*Me complex confirmed the NMR-determined hydrogen-bonding network. Detailed characterization of CV-N/mannoside complexes provides a better understanding of lectin-carbohydrate interactions and opens up to the use of CV-N and similar lectins as antiviral agents.

## Introduction

**Fig. 1:**
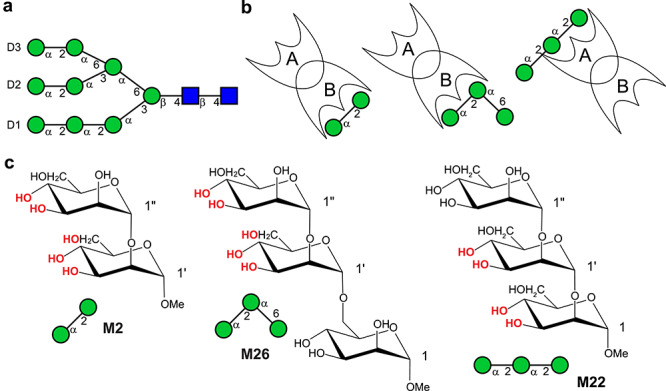
(**A**) Schematic representation of mannose-9 (Man-9) with mannose and *N*-acetylglucosamine units shown as green filled circles and blue filled squares, respectively. (**B**) Schematic view of CV-N (domain A and B) binding to Manα(1→2)Manα*O*Me (M2), Manα(1→2)Manα(1→6)Manα*O*Me (M26), and Manα(1→2)Manα(1→2)Manα*O*Me (M22). The high-affinity site for each mannoside is highlighted. (**C**) Structural formula of M2, M26 and M22. The experimentally observed hydroxyl groups in the CV-N/mannoside complexes are shown in red.

Cyanovirin-N (CV-N) is a small (11-kDa) lectin isolated from the cyanobacterium *Nostoc ellipsosporum*, which possesses antiviral activity against HIV and other enveloped viruses, such as Ebola and influenza (Barrientos and Gronenborn 2005; Koharudin and Gronenborn 2014). Its anti-HIV activity is mediated through binding to high mannose glycans (such as Man-9; Figure 1A) decorating the envelope glycoprotein gp120. Wild-type CV-N exists in solution as a monomer (Bewley et al*.* 1998), while in the crystal a domain-swapped dimer was observed (Yang et al*.* 1999). For application as an anti-HIV microbicide, a stabilizing mutant, P51G, was generated and this variant is commonly used. CV-N has two sugar binding sites (Figure 1B), one in each of its two domains (A and B), with the domain A binding site exhibiting a slight preference for a Manα(1→2)Manα(1→2)Manα trimannose unit, while the binding site on domain B preferentially interacts with a Manα(1→2)Manα dimannose unit (Bewley et al*.* 2002; Shenoy et al*.* 2002). Subsequent to the first structure determination of CV-N by NMR in 1998 (Bewley et al*.* 1998), additional structural studies by X-ray crystallography (Botos et al*.* 2002; Fromme et al*.* 2007; Fromme et al*.* 2008; Koharudin et al*.* 2013), NMR (Bewley 2001; Bewley et al*.* 2002; Nestor et al*.* 2017; Nestor et al*.* 2018; Shenoy et al*.* 2002) and computational (Ahlstrom et al*.* 2017; Margulis 2005; Vorontsov and Miyashita 2009) examinations were performed to elucidate further details of structure and sugar binding. CV-N since has become one of the most comprehensively characterized lectins, and as such frequently serves as a model system to develop and establish novel approaches for studying carbohydrate binding (Nestor et al*.* 2017; Nestor et al*.* 2018), protein folding (Koharudin et al*.* 2013), structure determination (Matei and Gronenborn 2016) and molecular dynamics (MD) simulations (Ahlstrom et al*.* 2017).

**Fig. 2:**
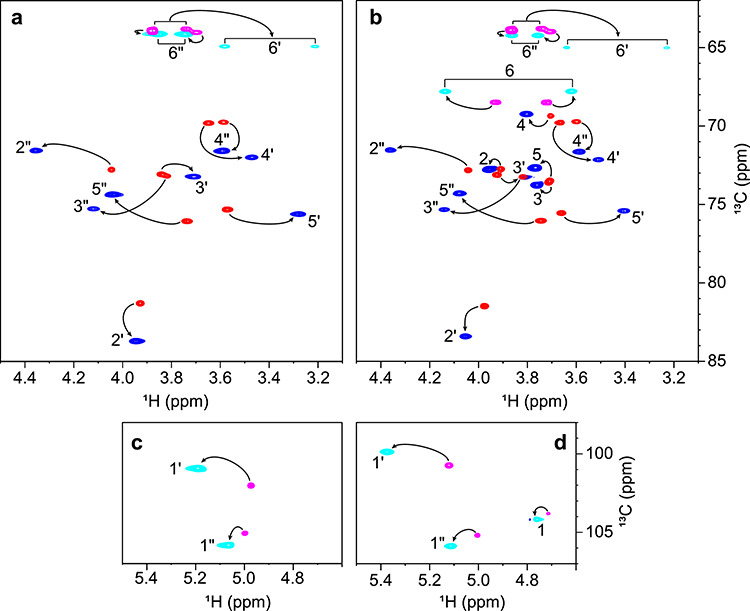
Superposition of the ring proton region (**A**, **B**) and anomeric region (**C**, **D**) of the ^1^H,^13^C-CT-HSQC spectra of free and CV-N bound M2 (**A**, **C**) and M26 (**B**, **D**). Resonances of the free and bound mannosides are shown in red/magenta and blue/cyan, respectively.

Despite this extensive body of work on CV-N, there are still open questions as to specifics of mannoside binding. Bewley (2001) determined the NMR structure of a complex between CV-N and Manα(1→2)Manα bound in the domain B binding site and proposed a hydrogen bonding network in this interaction. This NMR structure (PDB ID: 1IIY) was subsequently used by Margulis (2005) as the starting point for an MD simulation, which suggested that Glu41 and Arg76 side chains were binding to the sugar more tightly than other residues. These two residues were hypothesized to exhibit a cap-and-lock mechanism for the Arg76 side chain on the nanosecond timescale and a hydrogen bond between the Glu41 carboxylate group and the OH2″ of the dimannoside (see Figure 1C for OH numbering) was proposed (Margulis 2005). The crystal structure of the P51G-m4-CVN variant in which the sugar binding site in domain A had been abolished was solved with the dimannoside bound in the domain B binding site (Fromme et al*.* 2008). The overall structure of the protein backbone is preserved in this crystal structure (PDB accession code 2RDK), compared to the NMR structure, although the sugar is more intimately bound in the protein binding site and some amino acid side chains are differently oriented. The cap-and-lock mechanism suggested by Margulis (2005) was further inferred from the crystal structure (Fromme et al*.* 2008) but later questioned by Vorontsov and Miyashita (2009). They did not find any preferred conformations of the Arg76 side chain in their MD simulations in which the proposed hydrogen bond between the OH2″ hydroxyl and the Glu41 carboxylate group was seen only 9% of the time. As an alternative, they proposed a hydrogen bonding network with eight hydrogen bonds involving the OH3′, OH4′, OH3″ and OH4″ hydroxyls, with an average occupancy of 94% (Vorontsov and Miyashita 2009). Notably, none of these hydrogen bonds were suggested from the NMR structure (Bewley 2001).

We recently prepared the uniformly ^13^C-labeled trimannoside Manα(1→2) Manα(1→2)Manα*O*Me (M22) and assessed its structure when bound in the domain A binding site of CV-N (Nestor et al*.* 2018). Four hydroxyl protons were directly observed by NMR spectroscopy and the precise orientations of the hydroxyl groups was determined. Furthermore, a specific hydrogen bonding network was revealed. Here, we performed an analogous study to map the interactions in the domain B binding site. To that end, two different uniformly ^13^C-labeled carbohydrates were prepared: Manα(1→2)Manα*O*Me (M2) and Manα(1→2)Manα(1→6)Manα*O*Me (M26), both of which represent substructures of Man-9 with the highest affinity for the domain B binding site. The latter contains a (1→6)-linkage and thus mimics the entire D3 arm of Man-9 (Figure 1C). For each oligosaccharide, five hydroxyl protons were observed in the NMR spectra and conformational analysis by NMR permitted us to determine their orientations. Intermolecular NOEs between the sugar protons and the protein revealed similar structural features as in the 2RDK crystal structure (Fromme et al*.* 2008), although no protons are observed by crystallography. Our data also support the computational proposed hydrogen bonding network by Vorontsov and Miyashita (2009). The orientation and conformation of M26 bound to the B domain of CV-N were also investigated by molecular docking and MD simulations.

## Results and discussion

### Conformation of bound M2 and M26

Uniformly ^13^C-labeled mannosides were synthesized from ^13^C-labeled mannose and utilized for the current NMR investigations. Complexes of UL-^13^C M2 or M26, bound to the domain B binding site of ^15^N-labeled CV-N, were prepared using a 1.5:1 ratio for CV-N/M2 and a 1:1 ratio for CV-N/M26. The ^1^H,^13^C-HSQC spectra of the two carbohydrates (Figure 2) exhibited similar behavior, with chemical shift changes and line broadening effects upon CV-N binding, paralleling our earlier observations for the CV-N/M22 complex (Nestor et al*.* 2017). All chemical shifts (δ) and chemical shift differences (Δδ) between free and bound M2 and M26 are listed in Table SI. The largest proton chemical shift differences (Δδ_H_) were observed for the H6′a resonance of M2 (−0.54 ppm) and the H6′a resonance of M26 (−0.53 ppm), possibly due to the restricted rotation of the hydroxymethyl group in the bound sugar, compared to the free sugar. For the carbon shifts, Δδ_C_ were the largest for the C2′ resonance of M2 (+2.4 ppm) and the C5 resonance of M26 (−2.9 ppm). These differences may arise from differences in the conformation of the glycosidic linkages between the free and the bound form; the latter compound is known to be quite flexible at the ω torsion angle of its α(1→6)-linkage (Kotsyubynskyy et al*.* 2012), and restriction or alterations of conformation will be manifest in chemical shift changes upon binding.

Using ^13^C-labeled M2 and M26 permitted a detailed NMR investigation of the CV-N bound sugar conformations. Intramolecular NOEs in the bound M26 sugar were compared to those measured for M26 in solution. Differences between bound M26 and bound M2 were also evaluated. These, together with previous data obtained for free M2 (Lycknert et al*.* 2004; Säwén et al*.* 2010) allowed us to determine the bound conformations of the sugars. NOEs were measured using 1D selective T-ROESY spectra for free M26, and for both bound mannosides, ^1^H,^13^C-HSQC-NOESY spectra were recorded and distances were extracted from cross-peak intensities using the isolated spin-pair approximation. The data did not indicate any deviations from the common ^4^*C*_1_ ring conformation; the H3–H5 distances in both bound sugars is close to 2.6 Å, compatible with the 1,3-diaxial interaction in the ^4^*C*_1_ conformation (2.67 Å for H3′–H5′ of M2; 2.65 Å for H3′–H5′ of M26; 2.55 Å for H3″–H5″ of M26).

The conformation of the glycosidic (1″→2′)-linkage was determined using three inter-residue NOEs from H2′–H1″, H1′–H5″ and H1′–H1″. Experimental NOE-derived distances for bound M2 and M26 determined here were compared to those present in the crystal structure 2RDK (Fromme et al*.* 2008), the NMR structure 1IIY (Bewley 2001) and free M2 (Säwén et al*.* 2010). No significant differences were noted (Table SII). For the CV-N-bound M2, we measured dihedral angles of ϕ_H_ = −34° and ψ_H_ = 38°, very similar to those of the disaccharide bound in the domain B binding site in the 2RDK crystal structure (ϕ_H_ = −30°, ψ_H_ = 35°). Comparable results were also obtained for the (1″→2′) linkage in the bound M26 sugar. The derived dihedral angles are close to those in the major conformers described for the free disaccharide, e.g., ψ_H_ = 38° (Pendrill et al*.* 2019) or ϕ_H_ = −40°, ψ_H_ = 33° (Säwén et al*.* 2010) or ϕ_H_ = −29°, ψ_H_ = 20° (Zhang et al*.* 2019).

### Binding interface between M2/M26 and CV-N

**Fig. 3:**
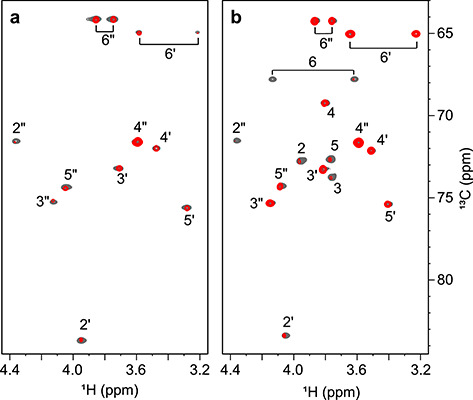
Superposition of a 2D version of a ^13^C-filtered NOESY-HSQC spectrum (red, 80 ms mixing time) and a ^1^H,^13^C-CT-HSQC spectrum (gray) of CV-N-bound M2 (**A**) and M26 (**B**).

In order to map the interaction between mannosides and CV-N at the atomic level, isotope-filtered and isotope-edited experiments were employed, using methodology previously developed by us for characterizing M22 bound to the domain A binding site of CV-N (Nestor et al*.* 2017). In the ^1^H,^13^C-dimension of ^13^C-filtered NOESY-HSQC experiments, NOESY cross-peaks between protein and sugar resonances are observed (Figure 3), yielding information important for characterizing the binding epitope of the glycan. As can be appreciated, the spectrum of the M26 complex is similar to that of the M2 complex, except that resonances associated with the reducing end residue of M26 exhibit lower intensities. This suggests that the two non-reducing end residues of M26, which are equivalent to M2, are the binding epitope, in accord with the fact that similar binding affinities were measured for M2 and M26.

The observed NOEs in the ^13^C-filtered NOESY-HSQC experiment for CV-N-bound M26, taking the ^1^H,^1^H-dimension of the experiment into account, revealed that magnetization transfer from the hydroxyl protons added a substantial contribution to the NOESY cross-peaks of the non-exchangeable H4′, H6′, H3″, H4″ and H6″ protons. To a lesser extent, a contribution from the *O*Me group, which was not ^13^C-labeled, to the cross-peaks of H2, H3 and H5 was also noted. In addition, an exchange contribution from water increased the intensity of the H2, H3, H4, H5 and H6″ cross-peaks. We therefore questioned whether the ^13^C-filtered NOESY-HSQC experiment alone is an unequivocally reliable instrument for mapping of the carbohydrate binding epitope, as we previously suggested (Nestor et al*.* 2017). However, since hydroxyl proton resonances were only observed for OH groups engaged in hydrogen bonds with the protein (see below), the observed additional NOE contribution confirms their tight interaction with the protein, although any quantitative extraction and use of intensities from ^13^C-filtered NOESY-HSQC experiments have to be treated with caution.

The binding interface for M26 on the protein was mapped using cross-peak information extracted from the ^1^H,^15^N-dimension of the CNH-NOESY experiment. The ^13^C-labeled ligand and ligand-protein NOEs yield information similar to that from chemical shift mapping (Nestor et al*.* 2017). In the CNH-NOESY spectrum of the CV-N/M26 complex (Figure S1b), NOEs were observed between the sugar and backbone amide proton resonances of N42, D44, G45, E56, T57, K74, R76 and Q78, as well as the side chain amino protons of N42, N53 and Q78. The equivalent amide protons were previously shown to undergo chemical shift changes between free and sugar-bound CV-N (Bewley et al*.* 2002). Backbone amide proton resonances of N42 and D44 and side chain amino proton resonances of N42, N53 and Q78 exhibited NOEs to M26 protons in the ^1^H,^13^C-HSQC-NOESY spectra (Figure S1a). Similar NOEs were observed in the CV-N/M2 complex, although fewer in numbers (Figure S2), compared to the CV-N/M26 complex.

### Detection of hydroxyl proton resonances

**Fig. 4:**
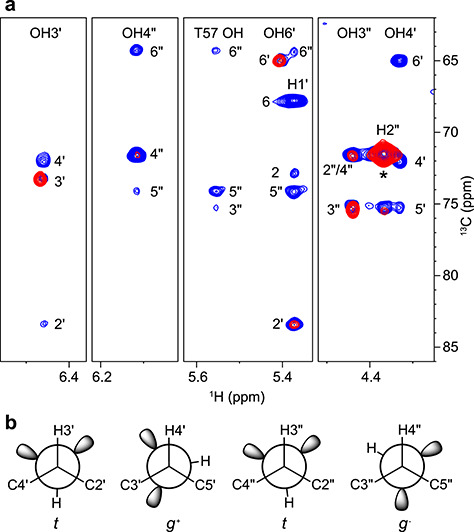
(**A**) Superposition of selected strips of the ^1^H,^13^C-HSQC-TOCSY (red, 10 ms mixing time) and the ^1^H,^13^C-HSQC-NOESY spectra (blue, 60 ms mixing time) of M26 bound to CV-N at 20°C. The H2″ HSQC cross-peak is marked with an asterisk. (**B**) Newman projections of M26 hydroxyl proton rotamers (OH3′, OH4′, OH3″ and OH4″), depicting the hydroxyl oxygen free electron pairs as orbital lobes. The carbon atom is positioned at the front and the hydroxyl oxygen at the back.

Five hydroxyl protons of CV-N-bound M2 and M26 were detected in the NMR spectra, and these are highlighted in Figure 1C. They were the OH3′, OH4′ and OH6′ hydroxyls of the middle mannose unit in M26 (reducing end sugar of M2) and the OH3″ and OH4″ hydroxyls of the non-reducing end mannose unit of M26 and M2. They were assigned from ^1^H,^13^C-HSQC-TOCSY spectra, in which scalar couplings (^3^*J*_HCOH_) to the vicinal positioned ring protons are present (Figure 4 and Figure S3). The much stronger intensities of the OH3′ and OH3″ correlations in the ^1^H,^13^C-HSQC-TOCSY spectra, compared to OH4′ and OH4″, indicated a *trans* orientation of OH3′ and OH3″ and a *gauche* orientation of OH4′ and OH4″. Hydroxyl proton resonances were not observed from the reducing end mannose of M26, most likely due to less intimate positioning of this reducing end sugar into the protein, suggesting a lack of persistent hydrogen bonds with the protein.

### Temperature coefficients of OH resonances

Temperature coefficients of hydroxyl proton resonances were extracted from a set of spectra recorded at temperatures from 5°C to 31°C (Table I and Table SIII). Similar to our previous findings for another CV-N complex (Nestor et al*.* 2018), the hydroxyl proton resonances are well dispersed from 4.33 ppm for OH4′ of M26 to 6.54 ppm for OH3′ of M2 at 20°C. Comparison of the bound M26 sugar resonances to those of the free oligosaccharide (Hakkarainen et al*.* 2007) revealed that the OH3′ resonance is downfield shifted by 0.5 ppm, while OH4′ and OH3″ resonances were upfield shifted by 1.7 and 1.3 ppm, respectively. Only small differences were observed for the OH6′ and OH4″ resonances. Given the numerous factors that influence the OH chemical shifts, such as hydrogen bonding, hydration and local structural changes, unambiguous interpretation as to the origin of the changes cannot be offered at the present time.

**Table I:** Summary of M26 hydroxyl proton data.

	OH3′	OH4′	OH6′	OH3″	OH4″	T57 OH
δ_H_^a^	6.46	4.33	5.41	4.42	6.12	5.55
Δδ^b^	0.5	−1.7	−0.2	−1.3	0.1	−0.6
Δδ/ΔT_bound_^a^	−5.5	−1.8	−9.3	−5.2	−3.2	−4.7
Δδ/ΔT_free_^b^	−11.7	−12.0	−12.4	−10.4	−11.2	
*θ*_HCOH_ ^c^	175	80	n. d.	155	−65	160
Rotamer	*t*	*g* ^+^		*t*	*g* ^−^	*t*
^3^ *J*_HCOH_ ^d^	14.5	−1.0		12.0	0.5	
HB_acceptor_	N42 C′O	K74 C′O		S52 C′O	N53 C′O	OH4″
*r*_OH-A_ ^e^	1.82	2.11		2.07	1.93	1.71
*θ*_OH-A_ ^e^	157	134		149	130	158
*θ*_C′O-H_ ^e^	140	124		147	120	
HB_donor_	D44 NH			N42 NH	T57 OH	N42 δNHb
*r*_D-OH_ ^e^	1.96			1.98	1.71	1.82
*θ*_DH-O_ ^e^	171			161	158	169

^a^NMR chemical shifts (δ_H_, ppm) at 20°C and temperature coefficients (Δδ/ΔT, ppb/K).

^b^δ_H_ and Δδ/ΔT of free M26 are from Hakkarainen et al*.* (2007). Chemical shifts of free M26 measured at −10°C in 85% H_2_O/15% acetone-*d*_6_ were extrapolated to 20°C. Δδ = δ(bound) − δ(free).

^c^OH dihedral angles (°) were determined from intramolecular OH-CH NOEs (see Table II).

^d^Coupling constants (Hz) were calculated from the Karplus relationship: ^3^*J*_HCOH_ = 5.76–2.05 cos *θ* + 6.78 cos (2*θ*), parameterized by Zhao et al*.* (2007).

^e^Hydrogen bond (HB) acceptor/donor distances (Å) and angles (°) were extracted from the X-ray structure model (PDB accession code 2RDK) after adjustment of the carbohydrate glycosidic dihedral angles and OH dihedral angles based on the NMR data.

For bound M2 and M26, the temperature coefficients ranged from −1.8 ppb/K for OH4′ of M26 to −9.9 ppb/K for OH6′ of M2 and are of similar magnitude to those previously observed for M22, which ranged from −1.2 to −5.8 ppb/K (Nestor et al*.* 2018). Not surprisingly, they are smaller than the temperature coefficients for hydroxyls of the free sugars, which range from −10.4 to −14.9 ppb/K (Hakkarainen et al*.* 2007), since the bound hydroxyl groups are mostly embedded in protein and less solvated than hydroxyls of a free sugar.

### Directionality of the hydroxyl groups

Intramolecular NOEs between hydroxyl protons and neighboring ring protons were used to derive distances, which, in turn allowed to extract hydroxyl proton dihedral angles (H–O–C–H) as described previously (Nestor et al*.* 2018). NOEs were extracted from ^1^H,^13^C-HSQC-NOESY spectra of the two CV-N/mannoside complexes for different mixing times from 10 to 120 ms (Figure 4A and Figure S3). At least two NOEs have to be available to determine the dihedral angle. Unfortunately, the OH4′ and OH3″ hydroxyl resonances of M2 and M26 are overlapped with the H2″ resonance, reducing the number of useful NOEs. In the case of M2, only one NOE could be obtained for OH3″, and for both complexes only one NOE was present for the OH6′ hydroxyl proton, thus precluding dihedral angle determination.

For M2 and M26, very similar OH dihedral angles were obtained and their values lie within 10 degrees of each other (Table I and Table SIII). The conformations with lowest root-mean-square deviation (RMSD) to distances derived from NOE data have OH3′ and OH3″ in the *trans* conformation (175–180° and 155°, respectively), whereas OH4′ and OH4″ are close to *gauche* (70–80°, *g*^+^ and −55 to −65°, *g*^−^, respectively). These values agree well with observations in the ^1^H,^13^C-HSQC-TOCSY spectra, which showed more intense cross-peaks for OH3′ and OH3″, caused by the larger ^3^*J*_HCOH_ couplings present in *trans* rotamers. In addition, intermolecular NOEs between T57 OH and sugar ring protons, as well as a NOE between T57 OH and T57 NH (*vide infra*), were used to predict the T57 OH dihedral angle to be close to a *trans* conformation (160°).

The above pattern is identical to our findings for the CV-N/M22 complex, where the hydroxyl group at position 3 was *trans* and the one at position 4 was *gauche* (Nestor et al*.* 2018). The preference for the *gauche* conformation of OH4 seems to be a general feature for lectin-bound sugars, while no general trend was seen for OH3 (Elgavish and Shaanan 1997).

Gratifyingly, the above determined NMR-derived distances and those extracted from the crystallographic models are within ±0.1 Å, with only a few exceptions (Table II). The models were generated by adding protons to the crystal structure of the CV-N/dimannoside complex (2RDK). The largest difference was found for OH3′ of M2.

**Table II:** NOE-derived distances (Å) between hydroxyl protons and mannoside ring protons, compared to distances derived from an X-ray structure (PDB accession code 2RDK).

		NOE derived^a^		Crystal structure^b^	
		*r*_M2_	*r*_M26_	*r*_M2_	*r*_M26_
OH3′	H2′	3.44	3.10	3.13	3.07
OH3′	H3′	2.99	2.93	2.85	2.85
OH3′	H4′	2.79	2.46	2.40	2.45
OH3′	H1″		3.83		3.81
OH3′	H3″		3.24		3.35
OH4′	H4′		2.46		2.42
OH4′	H5′	2.77	2.65	2.76	2.64
OH4′	H6′a/b	2.27	2.35	2.30^c^	2.10^c^
OH6′	H6′a/b	2.72	2.83		
OH3″	H2″/H4″		2.46		2.41^d^
OH3″	H3″	2.67	2.80		2.81
OH4″	H3″		2.77		2.76
OH4″	H4″	2.62	2.33	2.28	2.34
OH4″	H5″	3.50	3.20	3.51	3.47
OH4″	H6″a/b	3.17	3.16	3.17^c^	3.24^c^
T57 OH	H3″	2.99	2.78	2.97	2.97
T57 OH	H5″	3.17	3.08	3.15	3.15

^a^Standard errors are ≤ 0.09 for M2 and ≤0.07 for M26.

^b^Distances were derived after addition of hydroxyl protons based on the dihedral angles in Table I and Table SIII.

^c^The distance was calculated based on cross-relaxation involving the two H6(′/″) protons.

^d^The distance was calculated from OH3″ to H2″ (2.70 Å) and OH3″ to H4″ (2.71 Å).

### Sugar hydroxyl-protein contacts

**Fig. 5:**
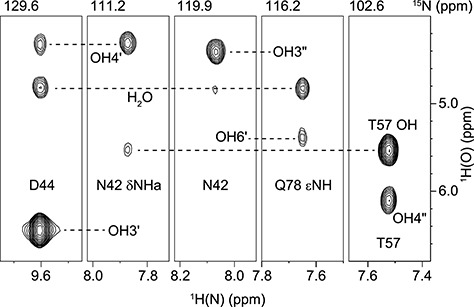
Selected strips of a ^13^C/^15^N-filtered NOESY-^1^H,^15^N-HSQC spectrum of the CV-N/M26 complex recorded at 20°C (50 ms mixing time).

Intermolecular NOEs between sugar OH protons and protein amide protons were measured in ^13^C/^15^N-filtered NOESY-^1^H,^15^N-HSQC spectra of ^13^C-labeled M26 complexed with ^13^C/^15^N-labeled CV-N (Figure 5). NOE build-up curves were generated using a series of mixing times (20–100 ms) for extraction of distances. The NOE-derived distances were compared to those from the 2RDK crystal (Fromme et al*.* 2008) and 1IIY NMR (Bewley 2001) structures of the CV-N/M2 complex (Table III). As can been noted, the distances obtained from the crystal structure are closer to the NOE-derived distances than those from the NMR structure. The former differ by ≤ 0.3 Å, except for OH4′, while the latter exhibit differences from 0.4 up to 5.7 Å. In the NMR structure the sugar is positioned slightly differently, compared to the crystal structure, translating into larger distances.

**Table III:** Intermolecular NOE-derived distances (Å) between M26 OH and CV-N amide protons, compared to distances derived from an X-ray structure (PDB accession code 2RDK) and an NMR structure (PDB accession code 1IIY).

M26	CV-N	*r*_exp_ ^a^	*r*_2RDK_ ^b^	*r*_1IIY_ ^b^
OH3′	D44	2.3	2.4	3.1
OH4′	N42 δNHa	2.5	3.2	7.7
OH4′	N42 δNHb	3.1	4.3	8.8
OH4′	D44	3.3	3.6	6.5
OH4′	G45	2.8	3.4	6.5
OH4′	R76	3.6	3.3	4.7
OH6′	Q78 εNHb	3.1	n. d. ^c^	n. d.^c^
OH3″	N42	2.5	2.5	2.1
OH3″	F54	3.3	3.6	4.8
OH4″	E56	3.0	3.2	4.1
OH4″	T57	2.9	3.2	3.4

^a^Standard errors are < 0.06.

^b^Hydroxyl protons were added based on the dihedral angles in Table I.

^c^Not determined since the conformation of OH6′ is not known.

In addition to NOEs between M26 hydroxyl protons and CV-N amide protons, the NOESY spectra also contained several NOEs between CV-N hydroxyls and amide protons. A total of 31 distances were extracted for three serines, S11, S20 and S32, and six threonines, T7, T19, T57, T61, T75 and T83 (Table SIV). These distances were compared to those extracted for the CV-N/M22 complex, in which the mannoside is bound in the domain A binding site. Only small differences were noted for these distances (≤0.2 Å); exceptions were the distances between T75 OH and R76 amide NH and between T57 OH and N42 δNHb. These differences may either reflect an alternative conformation for the N42 side chain in the two different glycan-bound structures or spin diffusion adversely affecting the size of the NOE. Overall, the fact that no substantive differences between the intra-protein NOEs for side-chain hydroxyls were found for these two different structures underscores that CV-N possesses a relatively fixed backbone structure that does not change significantly upon sugar binding.

### Hydrogen bonding network

**Fig. 6:**
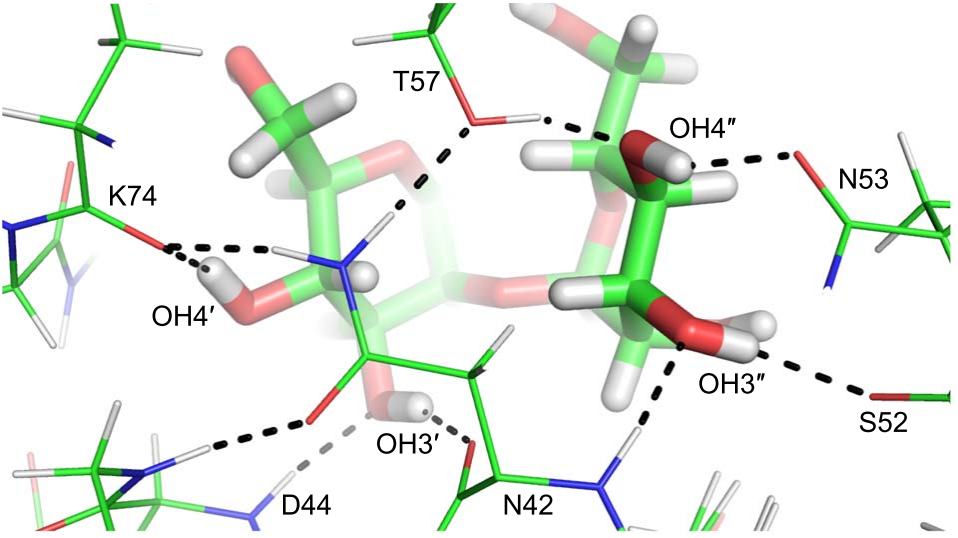
Molecular model depicting hydrogen bonds (by dashed lines) between M2 and CV-N. The model is based on the 2RDK crystal structure of a CV-N variant without the sugar binding site in domain A in the presence of M2, with hydroxyl protons added and positioned on the basis of NOE data.

Since the 2RDK crystal structure (Fromme et al. 2008)) of the CV-N/M2 complex agreed well with OH-NH NOE-derived distances, this structure was used for further analysis. Hydroxyl protons were added to the dimannoside in the crystal structure with dihedral angles set according to Table SIII, except for OH3″, which was set to 155° (Table I). The resulting structure reveals a hydrogen-bonding network in which all four OH protons point towards protein backbone carbonyl oxygens (Figure 6). The OH3′ hydroxyl proton donates a hydrogen bond to N42 C′O and D44 NH forms a hydrogen bond with the OH3′ oxygen. The OH4′ hydroxyl proton donates a hydrogen bond to K74 C′O, but the OH4′ oxygen is not hydrogen bonded. The OH3″ hydroxyl proton donates a hydrogen bond to S52 C′O and the N42 amide NH forms a hydrogen bond with the OH3″ oxygen. The OH4″ hydroxyl proton donates a hydrogen bond to N53 C′O and the T57 OH forms a hydrogen bond with the OH4″ oxygen. A similar hydrogen bonding network was observed for the CV-N/M22 complex (Nestor et al*.* 2018), where the sugar is bound in the other binding site and N42 is equivalent to N93, both forming key contacts with the sugar. Residue T57 is engaged in the same interactions as T7, D44 is equivalent to D95, and so on. The main difference is that the OH6′ hydroxyl was observed in the M2 (and M26) complex investigated here, but not in the M22 complex (Nestor et al*.* 2018). Since OH6′ was observed in the room temperature spectra, it may be possible that this hydroxyl is involved in hydrogen bonding. However, as we could not determine the orientation of the hydroxyl proton or the C6′ hydroxymethyl group with certainty, we refrain from proposing possible hydrogen bonds.

Since carbonyl carbon chemical shifts are sensitive to hydrogen bonding of the carbonyl oxygen, we also inspected the carbonyl carbon chemical shift differences (Δδ_C′_) between M26-bound and free CV-N (Figure S4). Hydrogen bond-accepting carbonyl carbons exhibited sizable differences, such as N42 C′O (−0.9 ppm), S52 C′O (−0.5 ppm), N53 C′O (+1.3 ppm) and K74 C′O (+0.2 ppm). However, other carbonyl carbons in the binding site also exhibited similar size Δδ_C′_ values, such as R76 C′O (+0.9 ppm), which may be caused by the interaction with the reducing-end sugar residue of M26. The upfield shift of T75 C′O (−0.5 ppm), on the other hand, may be the result of intra-residue hydrogen bonding. Clearly, several other carbonyl carbon resonances are affected by sugar binding, such as I40 C′O (−0.6 ppm) and Q79 C′O (+0.6 ppm). These carbonyls are located further away from the bound M26, and these chemical shift differences cannot be explained by a direct interaction with the sugar. Since both these carbonyls are within hydrogen bonding distance to amide protons (F54 NH and T75 NH, respectively) in the crystal structure of the complex, the associated chemical shift changes could be caused by small changes in the β-sheet structure upon M26 binding, reflecting the extremely sensitive nature of chemical shifts to any changes in structure of electronic environment.

At this juncture, it is worth pointing out that the capability of NMR to determine the correct orientation of hydroxyl protons is a unique feature and distinctive advantage of NMR over crystallography (Fromme et al*.* 2007; Fromme et al*.* 2008). Intriguingly, the experimentally determined hydrogen bonding network elucidated here resembles the proposed network suggested from MD simulations by Vorontsov and Miyashita (2009), and is clearly different from the predicted hydrogen bonding network based on the NMR structure 1IIY (Bewley 2001).

Our findings are also in line with earlier studies on CV-N mutants. Bolia et al*.* (2014) measured binding of M2 to single-site mutants of P51G-m4-CVN, a variant in which the domain A-binding site was abolished. The N42A mutation completely abolished binding, E41A or E41G reduced binding (*K_d_* = 0.4–0.5 mM), the T57A mutant exhibited low mM binding, and N53S did not affect binding significantly. N42 and T57 (and probably E41) are the only amino acids whose side chains are in direct hydrogen bond contact with the sugar.

### Molecular dynamics simulations of the CV-N/M26 complex

The prediction of hydroxyl orientations and the hydrogen bonding network from NMR data in combination with a crystal structure encouraged us to investigate whether a similar model could be obtained from molecular docking and MD simulations. The molecular docking procedure was tested for the CV-N/M26 complex. In total 15 conformational states of M26 were considered and the docked poses were evaluated by scoring with Vina in AutoDock (Trott and Olson 2010). Less likely poses were eliminated on the basis of the experimental OH-NH NOE data. Both, the number of OH-NH contacts < 5 Å and their numerical agreement with the experimental values were taken into account. Of the fifteen binding poses, the three top ones were examined further. Two of these were quite similar, with the M″ residue at the non-reducing end being identical in the two states, differing solely by the orientation of the hydroxyl protons. This agreement was less pronounced for the M′ and M residues. In the third pose, the orientation of the reducing end sugar residue pointed to the opposite side of the Arg76 residue. Since experimental data for the reducing end residue M were not available, it was not possible to unambiguously define which of the two poses represents the most probable one.

The latter docked structure, exhibiting the best agreement with the experimental data, was then solvated in a box of water and subjected to energy minimization, after which an MD simulation of the CV-N/M26 complex was performed by heating to 293 K. A final 50-ns production simulation yielded a stable RMSD of the atomic coordinates to within ~ 1.5 Å for the protein (Figure S5). RMSDs were also calculated for each mannosyl residue and plotted against their probability density distributions (Figure S6). The reducing end residue (M) shows a broader RMSD distribution compared with the other two residues (M′ and M′′), revealing a larger fluctuation of the atomic coordinates for the reducing end unit. An occupancy analysis shows the occurrence of seven key hydrogen bonds accounting for > 93% of the trajectory, namely, N42 NH—O3″, N53 C′O—OH4′′, S52 C′O—OH3″, K74 C′O—OH4′, N42 C′O—OH3′, T57 OH—O4″, D44 NH—O3′ (Table SV). Hydrogen bonds between the protein and the reducing end M residue were observed, although to a lesser degree. Arg76 engaged in three main interactions, namely, R76 η^1^NH—O2 (49%), R76 C′O—OH4 (33%), R76 C′O—OH3 (29%).

**Fig. 7:**
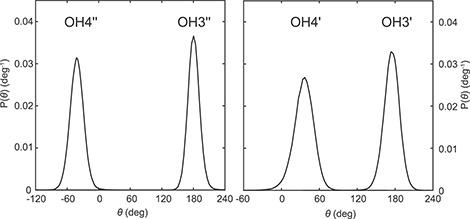
Kernel density estimation of the hydroxyl torsion probability densities of the M″ and M′ residues; (left) OH3″ and OH4″, and (right) OH3′ and OH4′, all of which are present in a single conformational state.

**Fig 8:**
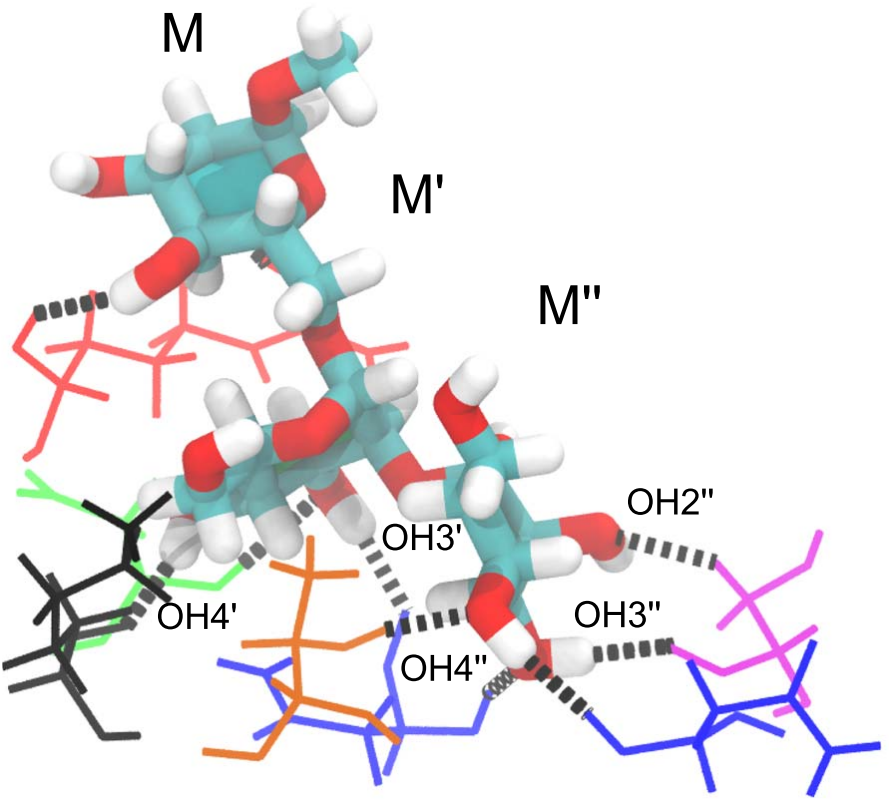
Binding pose extracted from clustering analysis using a 1-Å RMSD cutoff. This structure represents the main cluster (>80% of the frames). Hydrogen bonds between M26 and Arg76 (red), Asp44 (green), Lys74 (black), Thr57 (orange), Asn42 and Asn53 (blue; left and right, respectively) and Ser52 (pink) are illustrated by dashed lines.

The conformational states of the hydrogen bonding hydroxyl protons were identified as *gauche*^+^, *gauche*^−^ and *trans*; the conformation of the four hydroxyl groups of M26 agreed very well with those experimentally determined from the NMR data (Table SV), and the hydrogen bonds were present > 90% of the simulation time. The probability distributions indicate well-defined states for the secondary hydroxyl groups of M″ and M′, which are involved in the key hydrogen bonding interactions with CV-N (Figure 7 and Table SVI). Since rotation of hydroxyl groups in disaccharides in solution takes place on the order of a few tens of picoseconds (Pereira et al*.* 2006) the present 50-ns MD simulation should suffice to extract the preferred conformational states for hydrogen-bonded glycan hydroxyl groups in the CV-N/M26 complex. The OH6′ dihedral angle (OH6′–O6′–C6′–H6′_pro-*R*_), which could not be determined experimentally here by NMR (Table I), populated in the MD simulation not only the *g*^+^ conformation (71%) but also the *t* conformation (28%). The *g*^−^ conformation, however, only occurred transiently at a very low percentage (1%). The spatial proximity of the OH6′ hydroxyl proton to the Q78 εNHb hydrogen, which was clearly observed by NMR (Table III), was also supported by the hydrogen bonding arrangement with Q78 εNHb as a donor and O6′ as an acceptor (65%) as well as OH6′ as a donor and Q78 Oε^1^ as an acceptor (25%). Analysis of the conformational distribution at the glycosidic torsion angles of M26 in complex with CV-N shows a single conformational state at each glycosidic linkage (Figure S7 and Figure 8) with torsion angle averages in the M26 trisaccharide of 〈ϕ″〉 = −36.53°, 〈ψ″〉 = 38.67°, 〈ϕ′〉 = −46.04°, and 〈ψ′〉 = 180.83°, corresponding to an *exo*-anomeric *syn* conformation at the α(1→2)-linkage and an *exo*-anomeric *antiperiplanar* conformation at the α(1→6)-linkage (Pendrill et al*.* 2013; Yang et al*.* 2016). Overall, the hydrogen bonding geometries predicted from the MD simulations are consistent with those proposed from the NMR data.

## Conclusions

We determined the orientation of four hydroxyl protons of the bound sugar in the CV-N/M2 and CV-N/M26 complexes by NMR. The hydroxyls donate hydrogen bonds to four different backbone carbonyl oxygens of CV-N and are pivotal for the sugar–protein interaction. Key interactions around the side chains of T57 and N42 were identified. MD simulations of the carbohydrate–protein complex were fully consistent with the experimental observation. Our results highlight the complementarity of NMR, crystallography and MD simulations for identifying details in lectin–glycan interactions.

## Materials and methods

### Carbohydrate synthesis

Uniformly ^13^C-labeled Manα(1→2)Manα*O*Me (M2) and Manα(1→2)Manα(1→6)Manα*O*Me (M26) were synthesized from d-[^13^C_6_]-mannose. See Supplementary data for details.

### Protein expression and purification

P51G CV-N, uniformly labeled with ^15^N or ^13^C/^15^N, was prepared as described previously (Barrientos et al*.* 2004; Nestor et al*.* 2017). Samples for NMR were buffer-exchanged into 10 mM sodium phosphate buffer, 3 mM NaN_3_, 95/5% H_2_O/D_2_O, pH 6.6.

### NMR spectroscopy

NMR spectra were recorded over the temperature range 5–31°C on Bruker 600, 800, and 900 MHz AVANCE spectrometers, equipped with 5-mm-triple-resonance, *z*-axis gradient cryoprobes. Parameter settings for the NMR experiments are summarized in Table SVII. Assignments of M2, M26 and CV-N resonances were obtained essentially as described before (Nestor et al*.* 2017; Nestor et al*.* 2018). Spectra were referenced to internal DSS (δ_H_ = 0.00 ppm, δ_C_ = 0.00 ppm). For temperature calibration, an external methanol standard was used.

Intramolecular OH–CH and CH–CH cross-relaxation rates of CV-N-bound mannosides (^13^C labeled) were measured at 20°C and 900 MHz, using a sample of CV-N/M26 (1.0 mM; 1:1 molar ratio) and a sample of M2 (1.5 mM) and CV-N (1.0 mM; 1.5:1 ligand/protein molar ratio). 2D ^1^H,^13^C-HSQC-NOESY experiments were recorded with eleven different mixing times, ranging from 10 to 120 ms.

Intermolecular OH–NH cross-relaxation rates of the CV-N/M26 complex were measured at 20°C and 900 MHz for a 1.5:1 ligand/protein molar ratio of ^13^C-labeled M26 (0.9 mM) and ^13^C/^15^N-labeled CV-N (0.6 mM). 2D ^13^C/^15^N-filtered NOESY-^1^H,^15^N-HSQC experiments were recorded with ten different mixing times, ranging from 20 to 100 ms. In addition, a 3D version of the same experiment was recorded on the same sample for assignment purposes.

Proton–proton cross-relaxation rates of free M26 (without ^13^C-labeling) were measured at 20°C and 600 MHz on a 43 mM sample in 10 mM phosphate buffer in D_2_O (pD 7.0, equivalent to pH 6.6). 1D selective ^1^H,^1^H-T-ROESY spectra were recorded with 80 ms Gaussian shaped pulses, selective on H1, H1′, H1″, H2′, H2″, H3″ and H4″. Eight different mixing times ranging from 50 to 400 ms were used for each individual resonance. The recovery delay was set to 10 s to ensure > 5 × *T*_1_.

NMR spectra were processed with Topspin 3.5 (Bruker), and ccpNMR (Vranken et al*.* 2005) was used for resonance and NOE cross-peak assignments. Distances were calculated from NOE cross-peak intensities as previously described (Nestor et al*.* 2017; Nestor et al*.* 2018). The proton-proton cross-relaxation rates of free M26 were referenced to the H1″–H2″ distance (2.53 Å), which was set equal to the one in free M22 (Nestor et al*.* 2017). Intramolecular OH–CH cross-relaxation rates were referenced to the H1′–H2′ distance (2.57 Å), which was set equal to that determined for CV-N-bound M22 (Nestor et al*.* 2017). OH-NH cross-relaxation rates were referenced to the T75 OH–A77 NH distance of free CV-N (2.21 Å). This NOE cross-peak was observed in spectra of both, the CV-N/M22 (Nestor et al*.* 2018) and the CV-N/M26 complex.

Experimentally determined distances were compared to the equivalent distances from an X-ray structure (PDB accession code 2RDK; chain A) and an NMR structure (PDB accession code 1IIY) of the CV-N/M2 complex. Protons were added using PyMOL (The PyMOL Molecular Graphics System, Schrödinger, LLC). The glycosidic torsion angles of the (1″→2′)-linkage in M2 and M26 were adjusted (Table SII) to agree with the experimental NOE data. H–C–O–H dihedral angles (*θ*_HCOH_) were varied in 30° increments, and the corresponding distances were compared to the experimentally determined data by calculating RMSD. The lowest value of RMSD was considered to correspond to the optimum dihedral angle.

### Molecular docking and MD simulation

The NMR solution structure of wild type CV-N (Bewley et al*.* 1998) was used as the input coordinates and the program CarbBuilder was used to build the trisaccharide coordinate file (Kuttel et al*.* 2016).

Molecular docking was carried out in AutoDock VINA 1.1.2 (Trott and Olson 2010) in a simulation box of 22 Å, with the domain B of CV-N at its center. Torsions were set flexible for the following residues in the B-domain binding site: Glu41, Asn42, Val43, Asp44, Ser52, Asn53, Phe54, Glu56, Thr57, Lys74, Arg76, Gln78. The mannopyranosyl rings in the trisaccharide were fixed in the ^4^*C*_1_ conformation. The energies were explored with a 2 kcal·mol^−1^ range and 64 cycles per run. The resulting CV-N/M26 complex pose pdb files were evaluated for extracting low energy conformations.

MD and minimization were done using NAMD v. 2.12 (Phillips et al*.* 2005). Protein structure files for molecular dynamics of the complex were generated using the psfgen tool of VMD with the latest CHARMM force field, viz., CHARMM36 (Best et al*.* 2012; Brooks et al*.* 2009). The complex was solvated in a 70-Å box of water using a TIP3P water model (Durell et al*.* 1994). Ions were added to 0.2 M NaCl. An initial 1000 steps of potential energy minimization on solvent molecules was performed using conjugate gradient and line search algorithms. Harmonic restraints were applied to the complex with a force constant of 500 kcal mol^−1^ Å^−2^. A second minimization cycle without restraints was subsequently applied to the solvated complex. The system was then gradually heated to 293 K and equilibrated during 300 ps. A 50-ns production simulation was then performed in NPT ensemble with a time-step of 1 fs. A stochastic Langevin thermostat was used to keep the temperature constant at 293 K with a damping coefficient of 1 ps^−1^. The Nosé–Hoover Langevin piston pressure control method was employed to keep the pressure constant at 1 atm with a 100-fs oscillating time constant and a damping time constant of 50 fs. The particle mesh Ewald method was used to calculate non-bonded interactions with a grid spacing of 1 Å while electrostatic and van der Waals interactions were forced to zero at a 12 Å cutoff distance with switching functions smoothing the forces from 10 Å. ShakeH algorithm was used on bonds to the hydrogen atoms of the water molecules to keep them rigid. Coordinates were saved every 1 fs. Statistical analysis was carried out with MatLab R2017a using in-house scripts and the Circular Statistics Toolbox for directional statistics (Berens 2009). Hydrogen bonds were analyzed in VMD using > 135° and <3.4 Å as D–HA angle and DA distance criteria, respectively.

## Supplementary Material

Supplementary_data_cwaa081

## Abbreviations

CT: constant time
CV-N: cyanovirin-N
HIV: human immunodeficiency virus
HSQC: heteronuclear single-quantum coherence
M2: Manα(1→2)Manα*O*Me
M22: Manα(1→2)Manα(1→2)Manα*O*Me
M26: Manα(1→2)Manα(1→6)Manα*O*Me
MD: molecular dynamics
NMR: nuclear magnetic resonance
NOE: nuclear Overhauser effect
NOESY: nuclear Overhauser effect spectroscopy
PDB: Protein Data Bank
RMSD: root-mean-square deviation
T-ROESY: transverse rotating-frame nuclear Overhauser effect spectroscopy
TOCSY: total correlation spectroscopy
UL: uniformly labeled
