# Supplementary material for: A detailed picture of a protein–carbohydrate hydrogen-bonding network revealed by NMR and MD simulations
Source: Glycobiology. 2020 Sep 8;31(4):508–18. doi: 10.1093/glycob/cwaa081 (PMC8091458; doi:10.1093/glycob/cwaa081)

A detailed picture of a protein-carbohydrate hydrogen-bonding network revealed by NMR and MD simulations

Gustav Nestor[1,2,*], Alessandro Ruda[3], Taigh Anderson[4], Stefan Oscarson[4], Göran Widmalm[3], and Angela M. Gronenborn[1,*]

[1]Department of Structural Biology, University of Pittsburgh School of Medicine, Pittsburgh, Pennsylvania 15261, USA

[2]Department of Molecular Sciences, Swedish University of Agricultural Sciences, Uppsala, Sweden

[3]Department of Organic Chemistry, Stockholm University, Stockholm, Sweden

[4]Centre for Synthesis and Chemical Biology, University College Dublin, Belfield, Dublin 4, Ireland

Supplementary data

1. Supplementary tables (SI-SVII)....................S2
2. Supplementary figures (S1-S7)....................S8
3. Experimental for the synthesis of ^{13}C-labeled mannosides....................S12
4. NMR spectra of the synthesized products....................S21

Table SI. Chemical shifts (ppm) of free and CV-N-bound M2 and M26.

			H1<br>C1	H2<br>C2	H3<br>C3	H4<br>C4	H5<br>C5	H6a; H6b<br>C6	*O*Me
**M2**	Man″	Free	4.99	4.05	3.82	3.58	3.74	3.70; 3.89	
			105.1	72.8	73.1	69.7	76.0	64.0	
		Bound	5.07	4.36	4.12	3.59	4.04	3.74; 3.85	
			105.8	71.6	75.3	71.6	74.4	64.2	
		$\Delta\delta^a$	0.07	0.31	0.30	0.00	0.30	0.04; -0.04	
			0.7	-1.2	2.1	1.8	-1.7	0.2	
	Man′	Free	4.97	3.93	3.84	3.64	3.57	3.75; 3.87	3.37
			102.1	81.3	73.1	69.8	75.3	63.8	57.6
		Bound	5.19	3.95	3.71	3.47	3.28	3.21; 3.58	3.39
			100.9	83.7	73.2	72.0	75.6	64.9	57.4
		$\Delta\delta^a$	0.22	0.02	-0.13	-0.18	-0.29	-0.54; -0.29	0.02
			-1.1	2.4	0.1	2.2	0.3	1.1	-0.2
**M26**	Man″	Free	5.00	4.04	3.82	3.60	3.74	3.73; 3.86	
			105.2	72.8	73.3	69.8	76.0	63.9	
		Bound	5.11	4.36	4.14	3.58	4.08	3.75; 3.86	
			105.9	71.5	75.3	71.6	74.3	64.2	
		$\Delta\delta^a$	0.10	0.31	0.31	-0.02	0.33	0.02; 0.00	
			0.7	-1.3	2.0	1.9	-1.8	0.3	
	Man′	Free	5.11	3.98	3.92	3.67	3.71	3.75; 3.87	
			100.8	81.5	73.1	69.8	73.6	63.8	
		Bound	5.37	4.05	3.81	3.50	3.40	3.22; 3.64	
			99.9	83.4	73.3	72.2	75.4	65.0	
		$\Delta\delta^a$	0.26	0.07	-0.12	-0.16	-0.31	-0.53; -0.23	
			-0.9	1.9	0.2	2.3	1.8	1.2	
	Man	Free	4.71	3.92	3.71	3.71	3.67	3.73; 3.93	3.38
			103.8	72.9	73.6	69.4	75.6	68.5	57.6
		Bound	4.76	3.96	3.75	3.80	3.76	3.61; 4.14	3.40
			104.2	72.8	73.7	69.2	72.7	67.8	57.6
		$\Delta\delta^a$	0.06	0.04	0.04	0.10	0.10	-0.12; 0.21	0.03
			0.3	-0.1	0.2	-0.2	-2.9	-0.7	0.0

[a] $\Delta\delta = \delta_{bound} - \delta_{free}$

Table SII. Comparison of glycosidic dihedral angles (°) and distances (Å) over the (1″→2′)-linkage of M2 and M26 between different structures in the PDB, free and CV-N bound form from the present study.

	$2RDK_{monomer 1}$	$2RDK_{monomer 2}$	1IIY	$M2_{free}$	$M2_{bound}$[c]	$M26_{free}$[d]	$M26_{bound}$[c]
ϕ_H	-30	-29	-49	-40[a]	-34		-35
ψ_H	35	32	37	33[a]	38		39
$r_{H2'-H1''}$	2.15	2.07	2.39	2.2[a]	2.18	2.21	2.17
$r_{H1'-H5''}$	2.91	2.79	2.43	2.6[b]	2.82	n.d.[e]	2.80
$r_{H1'-H1''}$	2.99	3.02	3.37	3.0[a]	2.97	3.34	2.94

[a]From Säwén et al. (2010).
[b]From Lycknert et al. (2004).
[c]From $^{1}H,^{13}C$-HSQC-NOESY spectra of CV-N-bound M2/M26.
[d]From 1D T-ROESY spectra of M26.
[e]Could not be determined due to spectral overlap.

Table SIII. Summary of M2 hydroxyl proton properties. Chemical shifts (δ_H, ppm) at 20 °C and temperature coefficients ($\Delta\delta/\Delta T$, ppb/K) were obtained by NMR, with OH dihedral angles, coupling constants (Hz), and hydrogen bond (HB) acceptor/donor distances (Å) extracted from NOEs and a related X-ray model.

	OH3′	OH4′	OH6′	OH3″	OH4″	T57 OH
δ_H	6.54	4.39	5.40	4.42	6.10	5.56
$\Delta\delta^a$	0.7	-1.6	-0.1	-1.3	0.1	-0.6
$d\delta/dT_{bound}$	-5.5	-2.0	-9.9	-4.2	-3.7	-4.2
$d\delta/dT_{free}{}^a$	-14.2	-12.8	-14.9	-13.0	-13.1	
$\theta_{HCOH}{}^b$	180	70	n. d.	n. d.	-55	160
rotamer[b]	*t*	g^+			g^-	*t*
$^3J_{HCOH}{}^c$	14.6	-0.1			2.3	
$HB_{acceptor}$	N42 C′O	K74 C′O			N53 C′O	OH4″
$r_{OH\text{-}A}{}^d$	1.80	2.01			1.83	1.71
$\theta_{OH\text{-}A}{}^d$	161	146			143	158
$\theta_{C'O\text{-}H}{}^d$	139	127			123	
HB_{donor}	D44 NH			N42 NH	T57 OH	N42 δNHb
$r_{D\text{-}OH}{}^d$	1.96			1.98	1.71	1.82
$\theta_{DH\text{-}O}{}^d$	171			161	158	169

[a]δ_H and $\Delta\delta/\Delta T$ of free M2 are from Hakkarainen et al.[1] Chemical shifts of free M2 measured at -10 °C in 85% H_2O/15% acetone-d_6, were extrapolated to 20 °C. $\Delta\delta = \delta(\text{bound}) - \delta(\text{free})$. [b]OH dihedral angles were determined from intramolecular OH-CH NOEs (see Table 2). [c]Coupling constants were calculated from the Karplus relationship: $^3J_{HCOH} = 5.76 - 2.05 \cos\theta + 6.78 \cos(2\theta)$, parameterized by Zhao et al.[2] [d]Extracted from the X-ray structure model (PDB accession code 2RDK) after adjustment of the carbohydrate glycosidic dihedral angles and OH dihedral angles based on the NMR data.

Table SIV. NOE-derived distances (Å) between CV-N OH and CV-N amide protons. Distances from the CV-N/M26 complex are compared to distances from the CV-N/M22 complex previously published by Nestor *et al.*[3]

OH	NH	r_{M22}[a]	r_{M26}[a]
T7	Q6		4.2
T7	T7	2.4	
T7	C8		3.7[b]
T7	N93 δNHa		3.5[b]
T7	N93 δNHb	2.6	2.6
S11	S11	2.8	2.9
S11	S20		4.0[b]
T19	T19		3.7
S20	Y9		3.6
S20	S11	3.7	3.7
S20	T19		4.3[b]
S20	T21	2.8	3.0
S32	N30 δNHb		3.6[b]
S32	S32	2.7	2.7
S32	S33		3.6[b]
S32	L98	2.3	2.4
T57	N42 δNHb	2.5	2.8
T57	E56	3.5[b]	
T57	T57	2.4	2.5
T57	C58		4.1[b]
T61	G15	2.8	2.8
T61	T61	3.2	3.2
T75	T75	3.0	3.3
T75	R76	3.8	3.2[b]
T75	A77	2.2	2.3
T75	Q78	3.5	3.4
T75	Q79	2.5	2.5
T83	L47	2.3	2.1
T83	K84	2.3	2.3

[a]Standard errors were ≤0.05 Å for M22 and ≤0.06 Å for M26.
[b]Probably affected by spin diffusion.

Table SV. Average hydrogen bonding geometries from a 50-ns MD trajectory of the CV-N/M26 complex.

		Distance (Å) H-A	Distance (Å) D-A	Angle (°) D-H-A	Distance (Å) H-A Expt	Angle (°) D-H-A Expt
D44 NH	O3′	2.14 (0.20)[a]	3.10 (0.18)	161.44 (8.15)	1.96	171
N42 C′O	OH3′	1.94 (0.17)	2.84 (0.15)	153.45 (10.00)	1.82	157
K74 C′O	OH4′	1.93 (0.18)	2.85 (0.15)	159.65 (11.28)	2.11	134
N42 NH	O3″	1.96 (0.13)	2.94 (0.12)	166.23 (7.67)	1.98	161
S52 C′O	OH3″	1.89 (0.23)	2.82 (0.21)	160.77 (9.51)	2.07	149
T57 OH	O4″	2.05 (0.23)	2.95 (0.18)	155.47 (11.42)	1.71	158
N53 C′O	OH4″	1.88 (0.17)	2.82 (0.15)	162.96 (10.04)	1.93	130

[a] One standard deviation. Angular data are calculated using circular statistics and are expressed in degrees (°) while the arithmetic average is used for the H-A and D-A distances in Å.

Table SVI. Average torsion angles of hydroxyl groups and their conformations from a 50-ns MD trajectory of the CV-N/M26 complex.

Hydroxyl group	Torsion angle (°) Model	Conf. state	Torsion angle (°) Expt	Conf. state
OH3′	174.38 (12.45)[a]	*t*	175	*t*
OH4′	35.12 (15.69)[a]	g^{+}	80	g^{+}
OH3″	–179.09 (11.34)[a]	*t*	155	*t*
OH4″	–41.34 (13.23)[a]	g^{-}	–65	g^{-}

[a] One standard deviation. Torsion angle data are calculated using circular statistics.

Table SVII. Parameter settings for all NMR experiments.

Sample	Fig.	Experiment	Pulse sequence	B_0 (T)	NS	Dimensions	SW (ppm)	TD	AQ (ms)	T (°C)	Comments	Exp. time
^{13}C-M2/M26 + ^{15}N-CV-N	2, 3	^{1}H,^{13}C-CT-HSQC	hsqcctetgpsisp	21.1	4	F2-F1	8×60	1k×256	71×9	20	2T = 22 ms	28 min
^{13}C-M2/M26 + ^{15}N-CV-N	6, S2	2D ^{1}H,^{13}C-HSQC-TOCSY	hsqcdietgpsisp.2	21.1	32	F2-F1	14×140	2k×256	81×4	20	τ_{mix} = 10 ms	3.7 h
^{13}C-M2/M26 + ^{15}N-CV-N	4, 6, S1, S2	2D ^{1}H,^{13}C-HSQC-NOESY	hsqcetgpnosp	21.1	32	F2-F1	14×140	2k×256	81×4	20[a]	τ_{mix} = 10-120 ms	3.8 h/exp.
^{13}C-M2/M26 + ^{15}N-CV-N	4, S1	^{1}H,^{15}N-HSQC	fhsqcf3gpph	21.1	8	F2-F1	14×36	2k×256	81×39	20		56 min
^{13}C-M2/M26 + ^{15}N-CV-N	-	2D HC(C)H-TOCSY	hcchdigp3d	14.1	16	F3-F2	15×140	2k×256	114×6	20	τ_{mix} = 11 ms	2 h
^{13}C-M2/M26 + ^{15}N-CV-N	3	2D ^{13}C-filt. NOESY-^{1}H,^{13}C-HSQC	noesyhsqcgpwgx13d	21.1	32	F3-F2	8×60	1k×256	71×9	20	τ_{mix} = 80 ms	4 h
^{13}C-M2/M26 + ^{15}N-CV-N	4, S1	2D CNH-NOESY	noesycngp3d	21.1	32	F3-F2	14×36	2k×256	81×39	20	τ_{mix} = 60 ms	4 h
^{13}C-M26 + ^{13}C,^{15}N-CV-N	-	3D HNCACB	hncacbgpwg3d	14.1	40	F3-F2-F1	14×33×60	1k×38×100	61×9×6	20		54 h
^{13}C-M26 + ^{13}C,^{15}N-CV-N	-	3D CBCA(CO)NH	cbcaconhgpwg3d	14.1	32	F3-F2-F1	14×33×60	1k×38×100	61×9×6	20		44 h
^{13}C-M26 + ^{13}C,^{15}N-CV-N	-	3D HC(C)H-TOCSY	hcchdigp3d	14.1	16	F3-F2-F1	12×84×12	1k×88×112	71×3×8	20	τ_{mix} = 11 ms	72 h
^{13}C-M26 + ^{13}C,^{15}N-CV-N	-	3D HNCO	hncogpwg3d	14.1	16	F3-F2-F1	14×34×16	1k×46×64	61×11×13	20		16.5 h
^{13}C-M26 + ^{13}C,^{15}N-CV-N	-	2D ^{13}C/^{15}N-filt. NOESY-^{1}H,^{15}N-HSQC	noesyhsqcf3gpwgx13d	21.1	32	F3-F1	14×12	2k×256	81×12	20	τ_{mix} = 20-100 ms	3.8 h/exp.
^{13}C-M26 + ^{13}C,^{15}N-CV-N	7	3D ^{13}C/^{15}N-filt. NOESY-^{1}H,^{15}N-HSQC	noesyhsqcf3gpwgx13d	14.1	24	F3-F2-F1	11×34×11	1k×48×104	78×12×8	20	τ_{mix} = 50 ms	47 h
M26 (unlabeled)	-	1D selective ^{1}H,^{1}H-T-ROESY	selrogp.2	14.1	192	F1	6	16k	2,277	20	τ_{mix} = 50-400 ms	44 min/exp.

[a]A series of 2D ^{1}H,^{13}C-HSQC-NOESY experiments were also performed at different temperatures (5-31 °C) to obtain temperature coefficients.

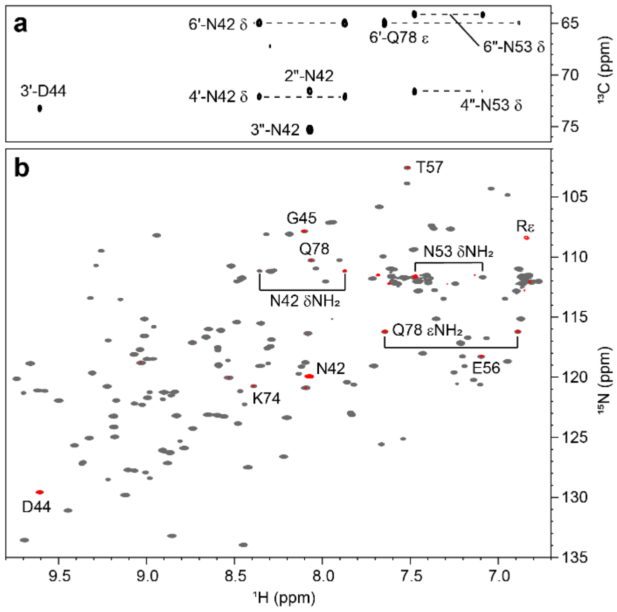

Figure S1. (a) Amide proton region of the ^{1}H,^{13}C-HSQC-NOESY spectrum (60 ms mixing time) of the CV-N/M26 complex. Intermolecular NOEs between sugar ring protons and protein amide protons are labeled. (b) ^{1}H,^{15}N 2D version of the CNH-NOESY spectrum (red, 60 ms mixing time) superimposed onto a ^{1}H,^{15}N-HSQC spectrum (gray).

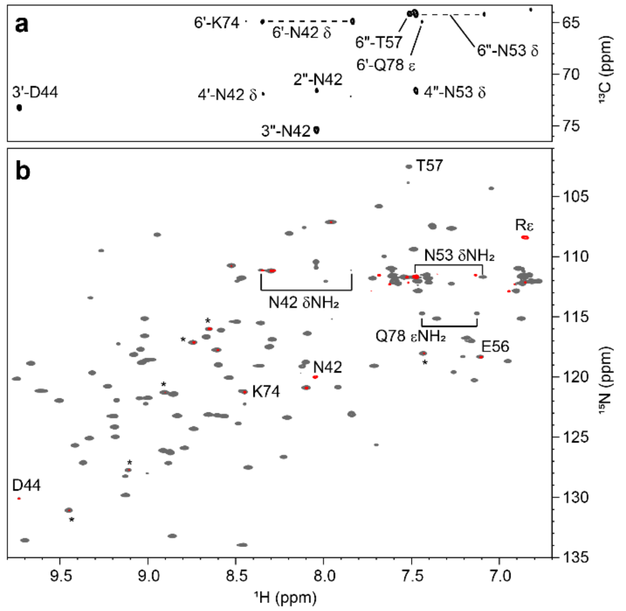

Figure S2. (a) Amide proton region of the ^{1}H,^{13}C-HSQC-NOESY spectrum (60 ms mixing time) of the CV−N/M2 complex. Intermolecular NOEs between sugar ring protons and protein amide protons are labeled. (b) ^{1}H,^{15}N 2D version of the CNH-NOESY spectrum (red, 60 ms mixing time) superimposed onto a ^{1}H,^{15}N-HSQC spectrum (gray). Asterisks (*) denote signals from the other binding site.

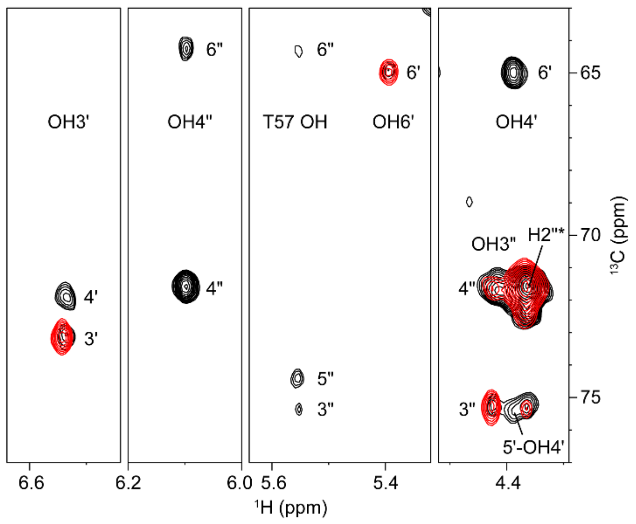

Figure S3. Selected strips of ^{1}H,^{13}C-HSQC-TOCSY (red, 10 ms mixing time) superimposed on ^{1}H,^{13}C-HSQC-NOESY spectra (black, 60 ms mixing time) of M2 bound to CV-N at 20 °C. The H2″ HSQC cross-peak is marked by an asterisk.

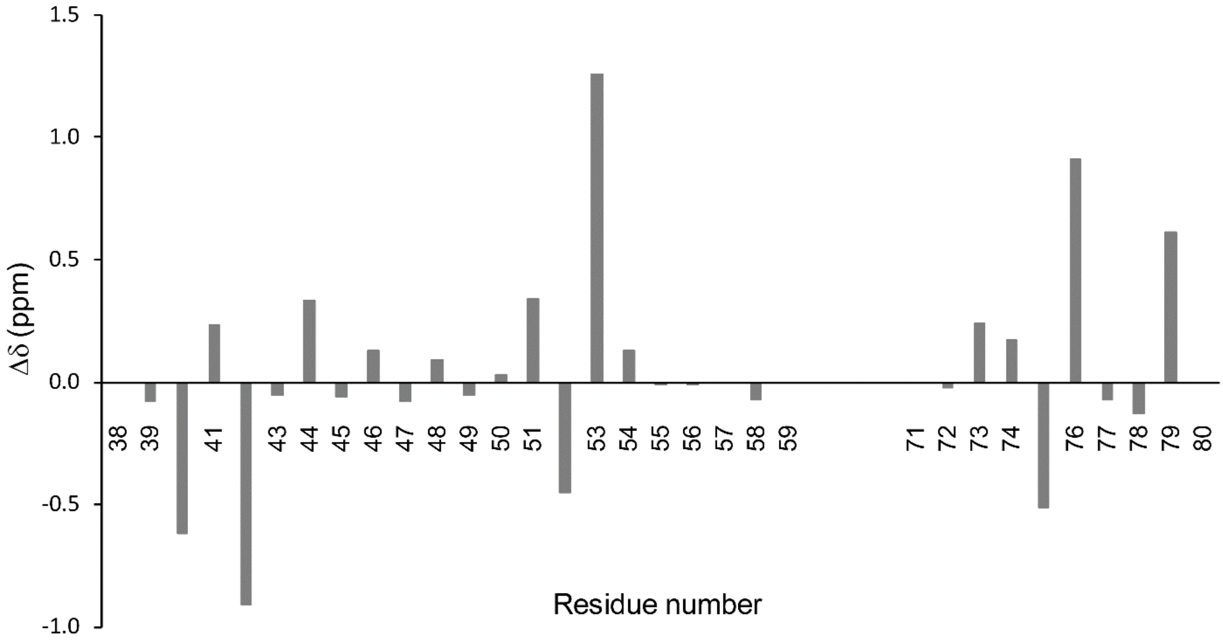

Figure S4. Plot of the backbone carbonyl carbon chemical shift differences between M26-bound and free CV-N. Δδ = δ(bound) – δ(free).

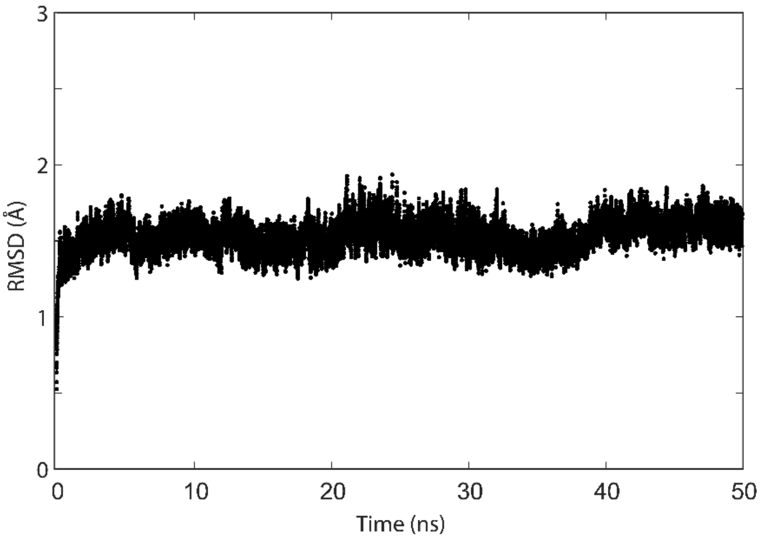

Figure S5. Backbone RMSD of the CV-N/M26 complex over a 50-ns MD trajectory with explicit water. The coordinates of all atoms were aligned relative to the first trajectory frame.

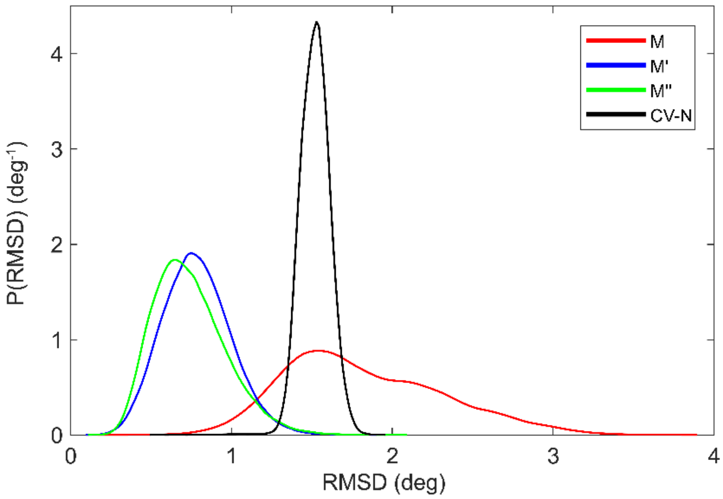

Figure S6. Probability distribution of all atom RMSDs for each sugar residue in the trisaccharide Manα(1→2)Manα(1→6)Manα*O*Me/CV-N complex over a 50-ns MD trajectory in explicit water. The coordinates of all atoms were aligned relative to the first trajectory frame.

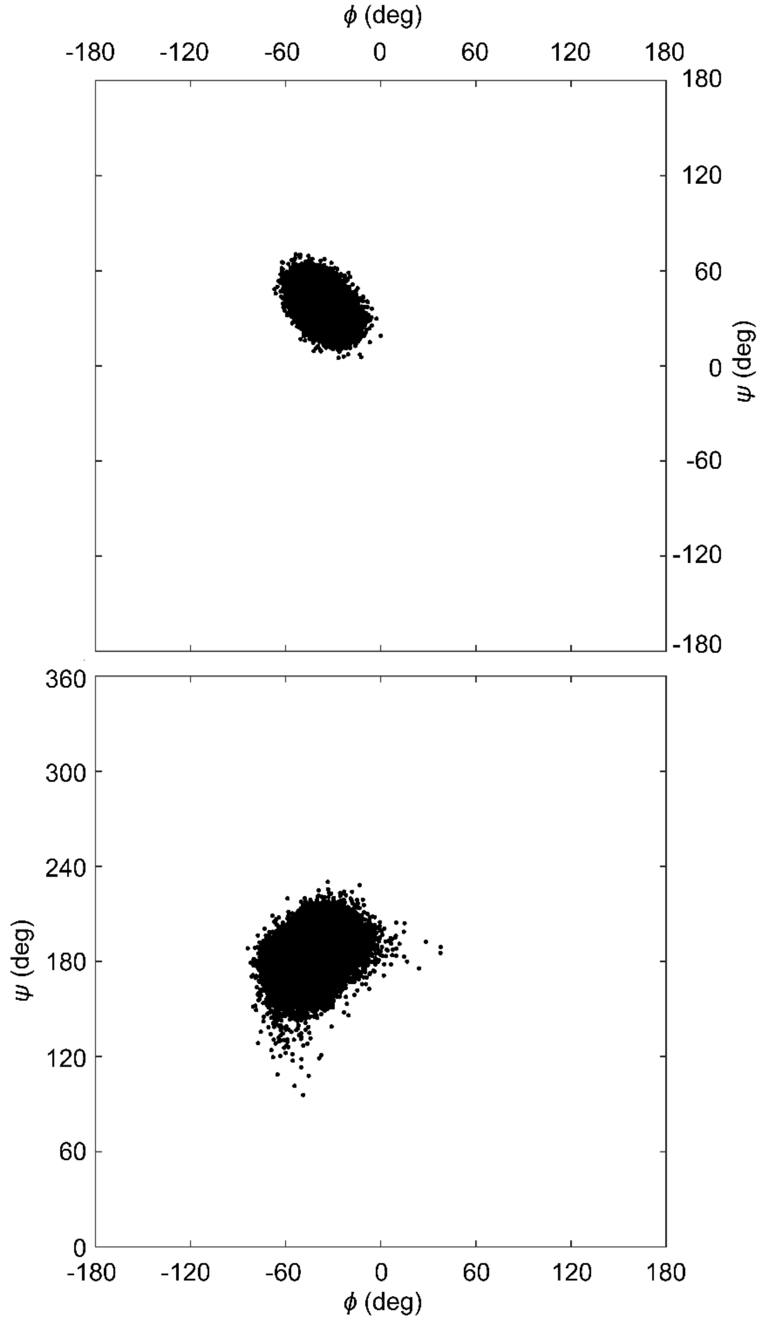

Figure S7. Scatter plots of the glycosidic torsion angles in Manα(1→2)Manα(1→6)Manα*O*Me: (top) α(1→2)-linkage and (bottom) α(1→6)-linkage.

Experimental for the synthesis of ^{13}C-labeled mannosides

General Experimental

The D-[U-$^{13}C_6$]Mannose was purchased from Omicron Biochemicals, while all other reagents and solvents were purchased from Sigma Aldrich, Fisher Scientific and Alfa Aesar. Thin layer chromatography was performed using Merck silica gel $60F_{254}$ glass plates and visualised under UV and/or with an 8% H_2SO_4 stain. Column chromatography was carried out using Davisil high-purity grade silica gel, 60Å pore size, 40-63 µm particle size and size exclusion chromatography was carried out using Biorad P2 gel. NMR spectra were recorded in $CDCl_3$, MeOD or D_2O at 25°C on Varian instruments (300, 400 and 600 MHz for ^1H and 126 and 151 MHz for ^{13}C) and were calibrated with respect to the residual solvent peak. The un-labeled compounds were synthesised in parallel with the labeled compounds **1-8** and the ^1H chemical shifts of the un-labeled compounds were reported.

Synthetic Scheme

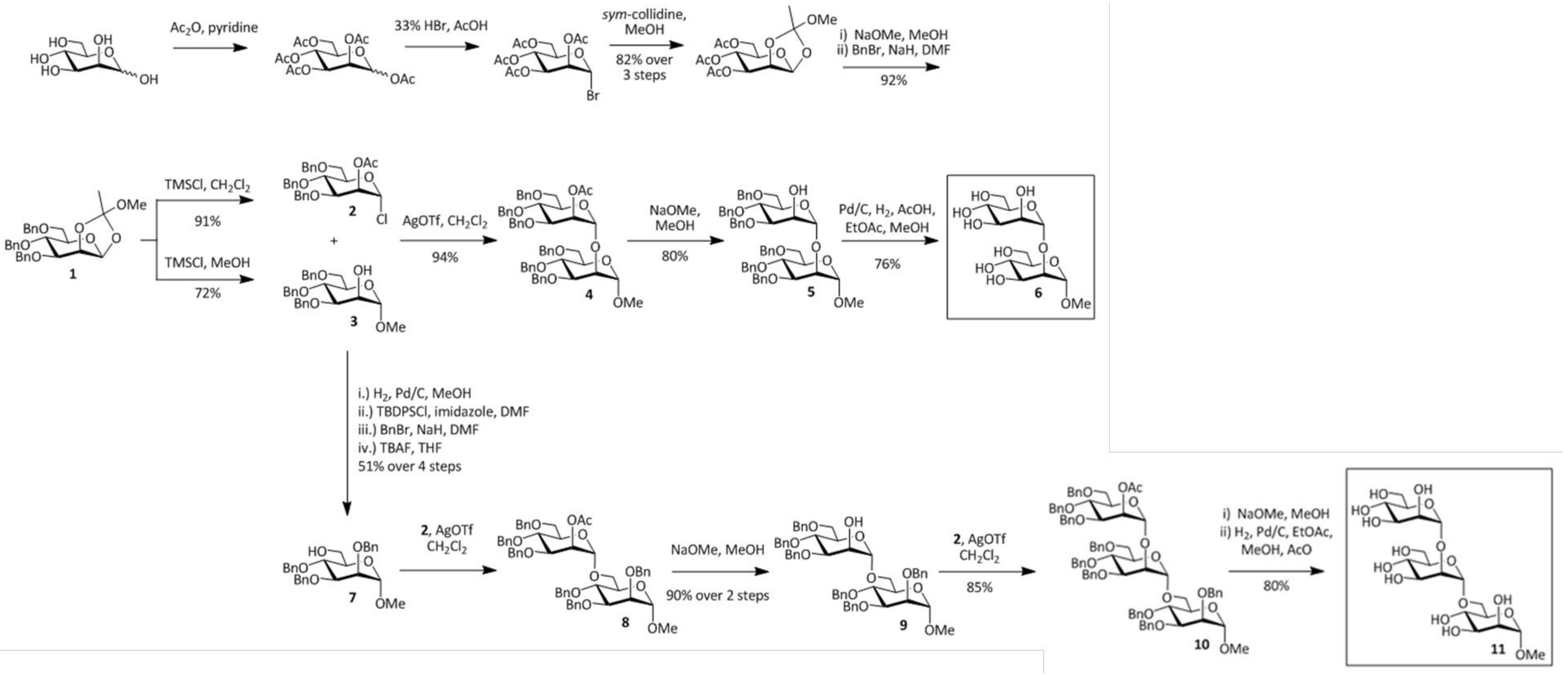

3,4,6-Tri-*O*-benzyl-1,2-*O*-(1-methoxy-ethylidene)-β-D-[$^{13}C_6$]mannopyranose (1).[4-5]

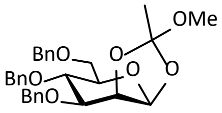

D-[$^{13}C_6$]Mannose (1.04 g, 5.58 mmoles) was dissolved in pyridine (11.3 mL) and acetic anhydride (10.6 mL) and stirred at r.t. for 15 hrs. The reaction mixture was diluted with CH_2Cl_2 (50 mL) and washed with 1M HCl (50 mL). The aqueous layer was washed with CH_2Cl_2 (3 x 100 mL) and the combined organic fractions were dried over $MgSO_4$ and concentrated.

The crude per acetylated D-[$^{13}C_6$]mannose (2.37 g, 5.98 mmoles) was dissolved in anhydrous CH_2Cl_2 (3 mL) under N_2 and cooled to 0°C. To this 33% HBr in acetic acid (2.62 mL, 14.95 mmoles) was added dropwise and this was stirred at 0°C for 15 minutes and then at r.t. for 17 hrs. The reaction mixture was diluted with CH_2Cl_2 (50 mL) and washed with aq. Na_2CO_3 (50 mL). The aqueous layer was then washed with CH_2Cl_2 (3 x 100 mL) and the combined organic fractions were dried over $MgSO_4$ and concentrated.

The crude bromosugar (2.34 g, 5.07 mmoles) was then dissolved in anhydrous CH_2Cl_2 (10 mL) and anhydrous MeOH (8 mL) and to this *sym*-collidine (1.6 mL) was added. This was stirred at 40°C for 16 hrs, after which time the reaction mixture was concentrated and precipitated from pentane and diethyl ether to yield the crude acetylated orthoester (1.68 g, 82% over three steps).

The acetylated orthoester (1.60 g, 4.35 mmoles) was dissolved in CH_2Cl_2 (1 mL) and MeOH (10 mL) and to this 1M NaOMe (0.44 mL, 0.44 mmoles) was added and stirred at r.t. for 30 mins. The reaction mixture was then concentrated and dried. The crude triol (1.2 g, 4.93 mmoles) was dissolved in anhydrous DMF (15 mL) under N_2 and cooled to 0°C. NaH (1.03 g, 25.88 mmoles) was added over 15 mins and then left to stir at this temperature for a further 15 mins. BnBr (2.93 mL, 24.65) was then added and left to stir at r.t. for a total of 15 hrs. The reaction mixture was quenched with MeOH (5 mL), concentrated and purified using flash silica gel chromatography (9.75:0.25 cyclohexane/Et_3N to 9:0.75:0.25 cyclohexane/EtOAc/Et_3N) to yield compound **1** (2.06 g, 92%, exo/endo = 97:3). NMR of exo product: ^{1}H NMR (300 MHz, $CDCl_3$) δ 7.44 – 7.16 (m, 15H), 5.35 (d, *J* = 2.5 Hz, 1H), 4.89 (d, *J* = 10.8 Hz, 1H), 4.78 (s, 2H), 4.60 (d, *J* = 11.3 Hz, 2H), 4.54 (d, *J* = 12.1 Hz, 1H), 4.40 (dd, *J* = 4.0, 2.5 Hz, 1H), 3.92 (t, *J* = 9.3 Hz, 1H), 3.79 – 3.66 (m, 3H), 3.48 – 3.36 (m, 1H), 3.28 (s, 3H), 1.73 (s, 3H).* ^{13}C NMR (151 MHz, $CDCl_3$) δ 138.2 (d, *J* = 2.7 Hz) (x2), 137.8 (d, *J* = 2.1 Hz), 128.5, 128.4, 128.3, 128.0, 128.0, 127.8, 127.5, 127.5, 97.6 (dd, *J* = 34.5, 3.8 Hz), 79.5 – 78.5 (m), 77.6 – 76.5 (m), 75.2, 74.7 – 73.6 (m), 73.3, 72.4, 69.5 – 68.3 (m), 49.8, 24.4. NMR data agrees with published values.[5]

2-*O*-acetyl-3,4,6-*O*-benzyl-α-D-[$^{13}C_6$]mannopyranosyl chloride (2).[6]

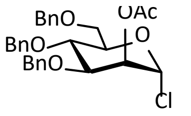

Compound **1** (230 mg, 0.45 mmoles) was dissolved in anhydrous CH_2Cl_2 (3.5 mL) under N_2. To this TMSCl (0.11 mL, 0.85 mmoles) was added and stirred at 45°C for 2 hrs. The reaction mixture was concentrated and dried to yield compound **3** (210 mg, 91%). This was used in the following step

*Indicates the ^{1}H chemical shifts of the un-labeled compounds were reported.

without any further purification. ^{1}H NMR (600 MHz, $CDCl_3$) δ 7.36 – 7.24 (m, 13H), 7.18 – 7.14 (m, 2H), 6.06 (bs, 1H), 5.46 (bs, 1H), 4.86 (d, *J* = 10.7 Hz, 1H), 4.70 (d, *J* = 11.2 Hz, 1H), 4.66 (d, *J* = 12.1 Hz, 1H), 4.57 (d, *J* = 11.2 Hz, 1H), 4.50 (dd, *J* = 11.4, 4.5 Hz, 2H), 4.25 (bd, *J* = 8.7 Hz, 1H), 4.08 – 4.02 (m, 1H), 3.98 (t, *J* = 10.2 Hz, 1H), 3.83 (dd, *J* = 11.2, 3.8 Hz, 1H), 3.69 (dd, *J* = 11.26, 1.76 Hz, 1H), 2.16 (s, 3H). ^{13}C NMR (126 MHz, $CDCl_3$) δ 170.1, 138.1 (d, *J* = 3.4 Hz), 137.9 (d, *J* = 2.4 Hz), 137.5 (d, *J* = 2.9 Hz), 128.5 (x2), 128.4 (x3), 128.1 (x2), 128.0 (x2), 127.9 (x2), 127.8 (x2), 127.7 (x2), 90.3 (dd, *J* = 45.1, 2.9 Hz), 77.3 – 76.2 (m), 75.4, 74.7 – 73.9 (m), 73.8 – 73.1 (m), 73.5, 72.2, 70.9 (dd, *J* = 44.9, 38.8 Hz), 68.3 – 67.6 (m), 20.9. NMR data agrees with published values.[6-7]

Methyl 3,4,6-tri-*O*-benzyl-α-D-[$^{13}C_6$]mannopyranoside (3).[4]

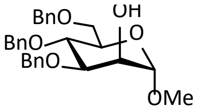

Compound **1** (263 mg, 0.51 mmoles) was dissolved in anhydrous MeOH (2.2 mL) under N_2. To this TMSCl (0.11 mL, 8.524 mmoles) was added and stirred at r.t. for 15 hours. The reaction mixture was diluted with CH_2Cl_2 (20 mL) and washed with aq. $NaHCO_3$. The aqueous layer was then washed with CH_2Cl_2 (3 x 100 mL) and the combined organic fractions were dried over $MgSO_4$, concentrated and purified using flash silica gel chromatography (8:2 cyclohexane/EtOAc) to yield compound **2** (173 mg, 72%). ^{1}H NMR (600 MHz, $CDCl_3$) δ 7.37 – 7.23 (m, 13H), 7.17 (dd, *J* = 7.5, 1.9 Hz, 2H), 4.82 (d, *J* = 10.9 Hz, 1H), 4.79 (bs, 1H), 4.72 – 4.64 (m, 2H), 4.66 (d, *J* = 1.6 Hz, 1H), 4.54 (d, *J* = 12.2 Hz, 1H), 4.50 (d, *J* = 10.9 Hz, 1H), 4.02 (bs, 1H), 3.89 – 3.80 (m, 2H), 3.78 – 3.68 (m, 3H), 3.36 (s, 3H). ^{13}C NMR (126 MHz, $CDCl_3$) δ 138.3 (d, *J* = 3.4 Hz), 138.2 (d, *J* = 2.3 Hz), 137.9 (d, *J* = 2.7 Hz), 128.5 (x2), 128.3 (x3), 127.9, 127.8 (x2), 127.8 (x3), 127.8 (x2), 127.6, 127.6, 100.3 (d, *J* = 48.6 Hz), 80.4 – 79.5 (m), 75.0, 74.2 (t, *J* = 41.4 Hz), 73.5, 72.0, 70.9 (ddt, *J* = 43.2, 40.8, 2.3 Hz), 68.9 (dt, *J* = 44.9, 3.2 Hz), 68.2 (dd, *J* = 48.5, 35.7 Hz), 54.9 (dd, *J* = 4.0, 2.0 Hz). NMR data agrees with published values.[6]

Methyl 2-*O*-acetyl-3,4,6-tri-*O*-benzyl-α-D-[$^{13}C_6$]mannopyranosyl-(1→2)-3,4,6-tri-*O*-benzyl-α-D-[$^{13}C_6$]mannopyranoside (4).

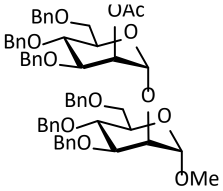

Procedure adapted from Bundle *et al.*[8] Compounds **2** (100 mg, 0.212 mmoles) and **3** (210 mg, 0.407 mmoles) were dried on the Schlenck line overnight then dissolved in CH_2Cl_2 (3 mL) under N_2, cooled to -15°C and stirred with activated 4Å molecular sieves (300 mg) for 45 mins. In a separate round bottom flask the AgOTf (256 mg, 0.996 mmoles) was dissolved in toluene (1 mL) and CH_2Cl_2 (1 mL) at r.t. under N_2 and stirred with activated 4Å molecular sieves (80 mg) for the same amount of time. This was then added dropwise to the mixture of compound **2** and **3** and this was left to slowly warm to r.t. over 4 hrs. The reaction mixture was then quenched with Et_3N (0.3 mL), filtered through Celite, concentrated and purified using flash silica gel chromatography (8:2 cyclohexane/EtOAc) to yield

compound **4** (191 mg, 94%). ^{1}H NMR (600 MHz, $CDCl_3$) δ 7.37 – 7.21 (m, 26H), 7.21 – 7.16 (m, 4H), 5.53 (bs, 1H), 5.07 (bs, 1H), 4.84 (dd, *J* = 10.8, 5.7 Hz, 2H), 4.77 (s, 1H), 4.67 (s, 3H), 4.65 (d, *J* = 2.9 Hz, 2H), 4.55 (dd, *J* = 11.6, 6.6 Hz, 2H), 4.49 (d, *J* = 12.0 Hz, 1H), 4.46 (d, *J* = 10.8 Hz, 1H), 4.40 (d, *J* = 10.9 Hz, 1H), 4.03 – 3.92 (m, 3H), 3.92 – 3.86 (m, 1H), 3.86 – 3.79 (m, 2H), 3.80 – 3.72 (m, 2H), 3.73 – 3.66 (m, 3H), 3.25 (s, 3H), 2.12 (s, 3H). ^{13}C NMR (126 MHz, $CDCl_3$) δ 138.5 (x2), 138.4, 138.4, 138.2, 138.0, 128.4, 128.3, 128.3, 128.3, 128.2, 128.1, 127.9, 127.8, 127.6, 127.6, 127.5, 127.5 (x2), 127.4, 127.4, 99.8 (d, *J* = 17.4 Hz), 99.4 (d, *J* = 17.9 Hz), 80.1 – 79.3 (m), 78.1 (dd, *J* = 41.6, 37.8 Hz), 75.1 (x2), 74.56 (td, *J* = 41.3, 28.6 Hz), 73.5, 73.3, 71.9 (x2), 71.7 (td, *J* = 42.5, 41.7, 19.1 Hz) (x3), 69.6 – 68.8 (m) (x2), 68.8 – 68.2 (m) (x3), 54.6, 21.1. NMR data agrees with published values.[6, 9]

Methyl 3,4,6-tri-*O*-benzyl-α-D-[$^{13}C_6$]mannopyranosyl-(1→2)-3,4,6-tri-*O*-benzyl-α-D-[$^{13}C_6$]mannopyranoside (**5**)

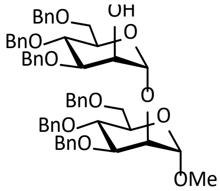

Compound **4** (185 mg, 0.195 mmoles) was dissolved in CH_2Cl_2 (1 mL) and MeOH (5.5 mL) and to this 1M NaOMe (78 µL, 0.078 mmoles) was added and stirred at r.t. for 4 hrs. Amberlite (IR 120 H+ form) resin (30 mg) was added and stirred for 30 mins. The reaction mixture was then filtered, concentrated and purified using flash silica gel chromatography (100% toluene to 9:1 toluene/acetone) to yield compound **5** (143 mg, 80%). ^{1}H NMR (600 MHz, $CDCl_3$) δ 7.37 – 7.22 (m, 26H), 7.19 (m, 4H), 5.14 (bs, 1H), 4.83 (dd, *J* = 13.8, 9.6 Hz, 2H), 4.80 (bs, 1H), 4.71 – 4.66 (m, 2H), 4.66 – 4.61 (m, 2H), 4.59 (d, *J* = 11.3 Hz, 1H), 4.55 (s, 2H), 4.55 – 4.47 (m, 3H), 4.12 (t, *J* = 3.3 Hz, 1H), 4.03 (s, 1H), 3.94 – 3.81 (m, 6H), 3.74 – 3.68 (m, 4H), 3.24 (s, 3H). ^{13}C NMR (151 MHz, $CDCl_3$) δ 138.6, 138.5 (d, *J* = 3.4 Hz), 138.4 (d, *J* = 3.4 Hz), 138.3 (d, *J* = 2.8 Hz), 138.2, 137.9 (d, *J* = 2.8 Hz), 128.5, 128.4, 128.3 (x2), 128.2, 127.9, 127.8, 127.7, 127.6, 127.5, 127.4, 127.3, 101.1 (d, *J* = 48.6 Hz), 99.8 (d, *J* = 47.9 Hz), 80.3 – 79.4 (m) (x2), 74.6 (dt, *J* = 50.3, 41.3 Hz) (x3), 73.4, 73.3, 71.6 (ddd, *J* = 44.5, 41.0, 23.6 Hz) (x2), 72.2, 72.1, 69.2 (ddt, *J* = 44.9, 21.1, 3.3 Hz) (x2), 68.5 (ddd, *J* = 48.6, 35.7, 3.4 Hz), 54.7 (dd, *J* = 4.0, 2.0 Hz). NMR data agrees with published values.[6, 9-10]

Methyl α-D-[$^{13}C_6$]mannopyranosyl-(1→2)-α-D-[$^{13}C_6$]mannopyranoside (**6**).

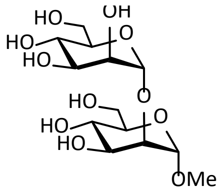

Compound **5** (64 mg, 0.070 mmoles) was dissolved in EtOAc (1.2 mL) and to this AcOH (0.3 mL) and a suspension of Pd/C (10% wt) (52 mg, 0.049 mmoles) in anhydrous MeOH (3 mL) was added. The round bottom flask was purged with hydrogen and left to stir under a balloon of H_2 at r.t. for 22 hrs. The reaction mixture was filtered through Celite, concentrated and purified using C-18 silica (9:1

H_2O/MeOH) followed by a normal phase flash silica gel column (6.75:3:0.25 DCM/MeOH/AcOH) to yield compound **6** (19 mg, 76%). ^{1}H NMR (400 MHz, MeOD) δ 4.98 (d, *J* = 1.8 Hz, 1H), 4.94 (d, *J* = 1.7 Hz, 1H), 3.98 (dd, *J* = 3.3, 1.8 Hz, 1H), 3.89 – 3.82 (m, 1H), 3.82 (dd, *J* = 3.5, 1.9 Hz, 2H), 3.79 (dd, *J* = 9.3, 3.3 Hz, 1H), 3.75 – 3.65 (m, 4H), 3.59 (t, *J* = 9.5 Hz, 2H), 3.47 (ddd, *J* = 9.8, 6.1, 2.4 Hz, 1H), 3.37 (s, 3H).* ^{13}C NMR (151 MHz, MeOD) δ 102.71 (d, *J* = 44.9 Hz), 99.67 (d, *J* = 47.6 Hz), 78.97 (ddd, *J* = 47.4, 38.2, 3.0 Hz), 73.31 (dt, *J* = 76.2, 41.7 Hz), 71.40 – 69.76 (m), 67.45 (td, *J* = 39.6, 31.0 Hz), 61.61 (ddd, *J* = 43.9, 8.2, 4.0 Hz), 53.84 (d, *J* = 4.2 Hz). NMR data agrees with published values.[11-12]

Methyl 2,3,4-tri-O-benzyl-α-D-[$^{13}C_6$]mannopyranoside (**7**).

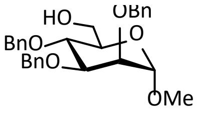

Compound **3** (270 mg, 0.56 mmoles) was dissolved in anhydrous MeOH (2 mL) and CH_2Cl_2 (2 mL) and to this a suspension of Pd/C (10% wt) (359 mg, 0.34 mmoles) in anhydrous MeOH (3 mL) was added. Using a Berghoff hydrogenator system, the reaction mixture was left to stir at 15 bar for 15.5 hrs at r.t. The reaction mixture was then filtered through Celite and concentrated.

The crude methyl mannopyranoside (154 mg, 0.77 mmoles) was dissolved in anhydrous DMF (2.2 mL) under N_2. To this imidazole (110 mg, 1.62 mmoles) and TBDPSCl (0.22 mL, 0.85 mmoles) were added and left to stir at r.t. for 4 hrs. The reaction mixture was diluted with EtOAc (10 mL) and washed with brine (50 mL). The aqueous layer was then washed with EtOAc (3 x 20 mL) and the combined organic fractions were dried over $MgSO_4$ and concentrated.

The crude triol (350 mgs, 0.80 mmoles) was dissolved in anhydrous DMF (4.5 mL) under N_2 and cooled to 0°C. NaH (128 mg, 3.19 mmoles) was added over 15 mins and then left to stir at this temperature for a further 15 mins. BnBr (0.38 mL, 3.19 mmoles) was then added and left to stir at r.t. for a total of 15 hrs. The reaction mixture was quenched with MeOH (2 mL), diluted with CH_2Cl_2 (20 mL) and washed with brine (40 mL). The aqueous layer was then washed with CH_2Cl_2 (3 x 20 mL) and the combined organic fractions were dried over $MgSO_4$ and concentrated.

The crude fully protected mannoside (210 mg, 0.29 mmoles) was dissolved in anhydrous THF (4 mL) under N_2 and to this 1M TBAF (0.74 mL, 0.74 mmoles) was added and stirred at r.t. for 15 hours. The reaction mixture was diluted with EtOAc (10 mL) and washed with brine (40 mL). The aqueous layer was then washed with EtOAc (3 x 20 mL) and the combined organic fractions were dried over $MgSO_4$, concentrated and purified using flash silica gel chromatography (8:2 cyclohexane/EtOAc to 1:1 cyclohexane/EtOAc) to yield compound **7** (137 mg, 51% over 4 steps). ^{1}H NMR (500 MHz, $CDCl_3$) δ 7.44 – 7.20 (m, 15H), 4.96 (d, *J* = 10.9 Hz, 1H), 4.80 (d, *J* = 12.3 Hz, 1H), 4.73 – 4.66 (m, 3H), 4.65 (s, 2H), 3.98 (t, *J* = 9.5 Hz, 1H), 3.92 (dd, *J* = 9.4, 3.0 Hz, 1H), 3.86 (dd, *J* = 11.7, 3.0 Hz, 1H), 3.82 – 3.76 (m, 2H), 3.64 (ddd, *J* = 9.6, 4.7, 3.0 Hz, 1H), 3.32 (s, 3H).* ^{13}C NMR (126 MHz, $CDCl_3$) δ 138.60 (d, *J* = 2.9 Hz), 138.54 (d, *J* = 3.4 Hz), 138.39 (d, *J* = 2.1 Hz), 128.54 (x2), 128.52 (x2), 128.51 (x2), 128.15 (x2), 127.97 (x2), 127.84 (x2), 127.71 (x3), 99.47 (d, *J* = 47.9 Hz), 80.97 – 79.64 (m), 75.64 – 74.16 (m), 73.08, 72.32, 72.13 (t, *J* = 41.7 Hz), 62.57 (dt, *J* = 42.0, 3.3 Hz), 55.19 – 54.42 (m). NMR data agrees with literature values.[13]

Methyl 2-*O*-acetyl-3,4,6-tri-*O*-benzyl-α-D-[$^{13}C_6$]mannopyranosyl-(1→6)-2,3,4-tri-*O*-benzyl-α-D-[$^{13}C_6$]mannopyranoside (8).

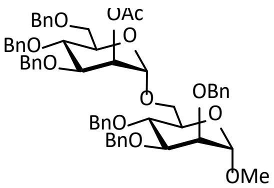

Compounds **2** (180 mg, 0.35 mmoles) and **7** (91 mg, 0.19 mmoles) were dried on the Schlenck line overnight then dissolved in CH_2Cl_2 (2 mL) under N_2, cooled to -15°C and stirred with activated 4Å molecular sieves (300 mg) for 45 mins. In a separate round bottom flask the AgOTf (149 mg, 0.58 mmoles) was dissolved in toluene (1 mL) and CH_2Cl_2 (1 mL) at r.t. under N_2. This was then added dropwise to the mixture of compounds **2** and **7** and left to stir at this temperature for 2 hrs. The reaction mixture was then quenched with Et_3N (0.2 mL), filtered through Celite and concentrated to yield compound **8** (232 mg, crude). ^{1}H NMR (500 MHz, $CDCl_3$) δ 7.41 – 7.13 (m, 30H), 5.48 (dd, *J* = 3.2, 1.9 Hz, 1H), 4.97 (d, *J* = 1.8 Hz, 1H), 4.92 (d, *J* = 11.2 Hz, 1H), 4.87 (d, *J* = 10.9 Hz, 1H), 4.73 (s, 2H), 4.72 (d, *J* = 1.8 Hz, 1H), 4.68 (d, *J* = 5.0 Hz, 1H), 4.65 (d, *J* = 4.2 Hz, 1H), 4.61 (d, *J* = 1.9 Hz, 2H), 4.53 – 4.41 (m, 4H), 3.97 (dd, *J* = 9.3, 3.2 Hz, 1H), 3.95 – 3.83 (m, 4H), 3.84 – 3.77 (m, 2H), 3.77 – 3.66 (m, 3H), 3.61 (dd, *J* = 10.8, 1.9 Hz, 1H), 3.27 (s, 3H), 2.16 (s, 3H).* ^{13}C NMR (126 MHz, $CDCl_3$) δ 170.30, 138.61 (d, *J* = 3.6 Hz), 138.47 (d, *J* = 4.3 Hz) (x2), 138.30 (t, *J* = 2.5 Hz) (x2), 137.84 (d, *J* = 3.0 Hz), 128.37 (x2), 128.34 (x3), 128.29 (x2), 128.25 (x3), 128.20 (x2), 127.83 (x2), 127.78 (x2), 127.74 (x2), 127.64 (x8), 127.55, 127.49, 127.48, 127.44, 98.70 (d, *J* = 47.7 Hz), 97.95 (d, *J* = 48.4 Hz), 80.26 (dd, *J* = 41.4, 38.2 Hz), 77.53 (dd, *J* = 41.8, 37.9 Hz), 75.05, 75.01 – 73.71 (m), 73.34, 71.13 (dt, *J* = 58.5, 42.8 Hz), 72.69, 71.99, 71.32, 68.97 – 68.00 (m), 66.70 (d, *J* = 45.2 Hz), 54.66, 21.16. NMR data agrees with literature values.[14]

Methyl 3,4,6-tri-*O*-benzyl-α-D-[$^{13}C_6$]mannopyranosyl-(1→6)-2,3,4-tri-*O*-benzyl-α-D-[$^{13}C_6$]mannopyranoside (9).

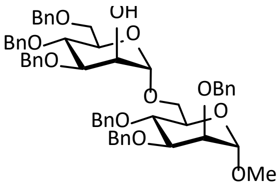

Crude compound **8** (232 mg, 0.24 mmoles) was dissolved in CH_2Cl_2 (0.2 mL) and MeOH (1.8 mL) and to this 1M NaOMe (49 µL, 0.05 mmoles) was added and stirred at r.t. for 4 hrs. Amberlite (IR 120 H^+ form) resin (30 mg) was added and stirred for 30 mins. The reaction mixture was then filtered, concentrated and purified using flash silica gel chromatography (8:2 cyclohexane/EtOAc to 7:3 cyclohexane/EtOAc) to yield compound **9** (160 mg, 90% over two steps). ^{1}H NMR (500 MHz, $CDCl_3$) δ 7.43 – 7.22 (m, 28H), 7.17 (dd, *J* = 7.3, 2.1 Hz, 2H), 5.09 (d, *J* = 1.7 Hz, 1H), 4.93 (d, *J* = 11.1 Hz, 1H), 4.84 (d, *J* = 10.9 Hz, 1H), 4.78 – 4.68 (m, 3H), 4.67 – 4.61 (m, 4H), 4.55 (dd, *J* = 22.6, 11.4 Hz, 2H), 4.49 (dd, *J* = 11.5, 9.4 Hz, 2H), 4.13 (t, *J* = 2.3 Hz, 1H), 3.95 – 3.85 (m, 5H), 3.83 – 3.72 (m, 3H), 3.72 – 3.65 (m, 2H), 3.62 (dd, *J* = 10.8, 1.9 Hz, 1H), 3.29 (s, 3H).* ^{13}C NMR (126 MHz, $CDCl_3$) δ 138.56 (d, *J* = 1.4 Hz), 138.53 (d, *J* = 1.3 Hz), 138.49 (d, *J* = 2.8 Hz), 138.36 (d, *J* = 2.1 Hz), 138.32 (d, *J* = 2.3 Hz), 137.87 (d, *J* = 2.6 Hz), 128.49 (x2), 128.38 (x2), 128.36 (x2), 128.33 (x2), 128.29 (x2), 128.27 (x2), 128.00 (x2), 127.89

(x2), 127.85 (x2), 127.78 (x2), 127.68 (x3), 127.67 (x3), 127.59, 127.53 (x2), 127.50, 99.62 (d, J = 48.3 Hz), 98.80 (d, J = 47.4 Hz), 80.23 (dd, J = 41.5, 38.3 Hz), 79.90 – 79.03 (m), 75.24 – 73.72 (m), 73.38, 72.73, 72.06, 71.43, 71.21 (td, J = 42.2, 41.5, 33.5 Hz), 68.83 (dt, J = 44.6, 3.1 Hz), 68.08 (ddd, J = 48.2, 35.7, 3.3 Hz), 66.30 (dd, J = 44.9, 3.5 Hz), 54.71 (t, J = 2.9 Hz). NMR data agrees with literature values.[14]

Methyl 2-O-acetyl-3,4,6-tri-*O*-benzyl-α-D-[$^{13}C_6$]mannopyranosyl-(1→2)-3,4,6-tri-*O*-benzyl-α-D-[$^{13}C_6$]mannopyranosyl-(1→6)-2,3,4-tri-*O*-benzyl-α-D-[$^{13}C_6$]mannopyranoside (**10**).

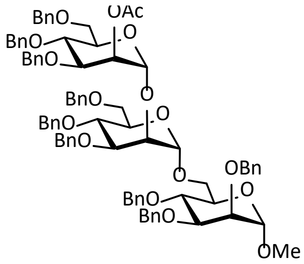

Compounds **2** (153 mg, 0.297 mmoles) and **9** (150 mg, 0.165 mmoles) were dried on the Schlenck line overnight, dissolved in CH_2Cl_2 (3 mL) under N_2, cooled to -15°C and stirred with activated 4Å molecular sieves (275 mg) for 45 mins. In a separate round bottom flask the AgOTf (127 mg, 0.495 mmoles) was dissolved in toluene (0.5 mL) and CH_2Cl_2 (0.5 mL) at r.t. under N_2. This was then added dropwise to the mixture of compound **2** and **9** and this was left to slowly warm to r.t. over 2 hrs. The reaction mixture was then quenched with Et_3N (0.3 mL), filtered through Celite, concentrated and purified using flash silica gel chromatography (9:1 cyclohexane/EtOAc) to yield compound **10** (194 mg, 85%). ^{1}H NMR (500 MHz, $CDCl_3$) δ 7.40 – 7.12 (m, 45H), 5.55 (dd, J = 3.2, 1.9 Hz, 1H), 5.11 (d, J = 1.8 Hz, 1H), 5.01 (d, J = 1.9 Hz, 1H), 4.90 (d, J = 11.1 Hz, 1H), 4.86 (dd, J = 10.9, 8.9 Hz, 1H), 4.74 – 4.43 (m, 15H), 4.39 (d, J = 11.0 Hz, 1H), 4.12 (t, J = 2.4 Hz, 1H), 4.02 – 3.73 (m, 12H), 3.71 – 3.56 (m, 5H), 3.24 (s, 3H), 2.13 (s, 3H).* ^{13}C NMR (126 MHz, $CDCl_3$) δ 171.27, 138.63, 138.60, 138.54, 138.50, 138.42, 138.28 (d, J = 1.8 Hz) (x2), 138.24, 138.06, 128.34 (x5), 128.28 (x2), 128.25 (x3), 128.22 (x2), 128.20 (x4), 128.15 (x2), 127.90 (x4), 127.78 (x5), 127.73 (x2), 127.63 (x3), 127.62 (x4), 127.58 (x2), 127.55, 127.50, 127.47, 127.43 (x2), 127.28 (x2), 99.66 (d, J = 49.0 Hz), 99.06 (d, J = 47.8 Hz), 98.96 (d, J = 47.4 Hz), 80.52 (t, J = 39.8 Hz), 79.68 – 77.53 (m), 75.02, 74.91, 74.87, 74.73,73.31, 73.17, 72.63, 72.10, 71.92, 71.53 (dt, J = 79.0, 42.6 Hz), 71.46, 69.64 – 68.12 (m), 66.68 (d, J = 44.9 Hz), 54.76, 21.20.

Methyl *O*-α-D-[$^{13}C_6$]mannopyranosyl-(1→2)-*O*-α-D-[$^{13}C_6$]mannopyranosyl-(1→6)-*O*-α-D-[$^{13}C_6$]mannopyranoside (**11**).

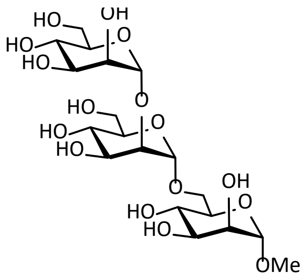

Compound **10** (250 mg, 0.179 mmoles) was dissolved in MeOH (3 mL) and CH_2Cl_2 (0.5 mL) and to this 1M NaOMe (54 µL, 0.100 mmoles) was added and stirred at r.t. for 4 hrs. Amberlite (IR 120 H^+ form) resin (20 mg) was then added and stirred for 30 mins. The reaction mixture was then filtered and concentrated to yield the crude benzylated trimannoside (223 mg).

A portion of the crude benzylated trimannoside (55 mg, 0.041 mmoles) was dissolved in EtOAc (0.8 mL) and to this AcOH (0.2 mL) and a suspension of Pd/C (10% wt) (39 mg, 0.037 mmoles) in anhydrous MeOH (2 mL) was added. Using a Berghoff hydrogenator system, the reaction mixture was left to stir at 15 bar for 16.5 hrs at r.t. The reaction mixture was then filtered through Celite, concentrated and purified using P2 size exclusion chromatography to yield compound **11** (17.7 mg, 80%).^{1}H NMR (500 MHz, D_2O) δ 5.14 (d, *J* = 1.8 Hz, 1H), 5.02 (d, *J* = 1.8 Hz, 1H), 4.74 (d, *J* = 1.6 Hz, 1H), 4.07 (dd, *J* = 3.4, 1.8 Hz, 1H), 4.01 (dd, *J* = 3.4, 1.8 Hz, 1H), 3.98 – 3.91 (m, 3H), 3.88 (dd, *J* = 11.9, 1.8 Hz, 2H), 3.84 (dd, *J* = 9.7, 3.4 Hz, 1H), 3.80 – 3.66 (m, 9H), 3.62 (t, *J* = 9.7 Hz, 1H), 3.40 (s, 3H).* ^{13}C NMR (126 MHz, D_2O) δ 101.85 (d, *J* = 44.9 Hz), 100.55 (d, *J* = 45.9 Hz), 97.49 (d, *J* = 47.2 Hz), 78.19 (dd, *J* = 47.3, 38.1 Hz), 72.51 (dt, *J* = 60.0, 41.5 Hz), 70.84 – 68.69 (m), 66.87 – 65.63 (m), 65.27 (d, *J* = 44.5 Hz), 60.54 (ddt, *J* = 43.3, 23.5, 3.1 Hz), 54.32. NMR data agrees with published values.[11-12, 15]

References

(1) Hakkarainen, B.; Kenne, L.; Lahmann, M.; Oscarson, S.; Sandström, C., *Magn. Reson. Chem.* **2007,** *45*, 1076-1080.

(2) Zhao, H. Q.; Pan, Q. F.; Zhang, W. H.; Carmichael, I.; Serianni, A. S., *J. Org. Chem.* **2007,** *72*, 7071-7082.

(3) Nestor, G.; Anderson, T.; Oscarson, S.; Gronenborn, A. M., *J. Am. Chem. Soc.* **2018,** *140*, 339-345.

(4) Franks, N. E.; Montgomery, R., *Carbohydr. Res.* **1968,** *6*, 286-+.

(5) Zhang, Y. M.; Mallet, J. M.; Sinaÿ, P., *Carbohydr. Res.* **1992,** *236*, 73-88.

(6) Ogawa, T.; Katano, K.; Sasajima, K.; Matsui, M., *Tetrahedron* **1981,** *37*, 2779-2786.

(7) Chang, C. W.; Chang, S. S.; Chao, C. S.; Mong, K. K. T., *Tetrahedron Letters* **2009,** *50*, 4536-4540.

(8) Dang, A. T.; Johnson, M. A.; Bundle, D. R., *Org. Biomol. Chem.* **2012,** *10*, 8348-8360.

(9) Jain, R. K.; Liu, X. G.; Oruganti, S. R.; Chandrasekaran, E. V.; Matta, K. L., *Carbohydr. Res.* **1995,** *271*, 185-196.

(10) Maity, S. K.; Ghosh, R., *Synlett* **2012**, 1919-1922.

(11) Sandström, C.; Berteau, O.; Gemma, E.; Oscarson, S.; Kenne, L.; Gronenborn, A. M., *Biochemistry* **2004,** *43*, 13926-13931.

(12) Sandström, C.; Hakkarainen, B.; Matei, E.; Glinchert, A.; Lahmann, M.; Oscarson, S.; Kenne, L.; Gronenborn, A. M., *Biochemistry* **2008,** *47*, 3625-3635.

(13) Gaunt, M. J.; Yu, J. Q.; Spencer, J. B., *J. Org. Chem.* **1998,** *63*, 4172-4173.

(14) Li, Z. J.; Li, H.; Cai, M. S., *Carbohydr. Res.* **1999,** *320*, 1-7.

(15) Khan, S. H.; Piskorz, C. F.; Matta, K. L., *J. Carbohydr. Chem.* **1994,** *13*, 1025-1035.

^{1}H NMR spectrum of 3,4,6-tri-*O*-benzyl-1,2-*O*-(1-methoxy-ethylidene)-β-D-mannopyranose (1).*

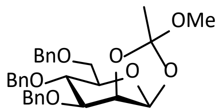

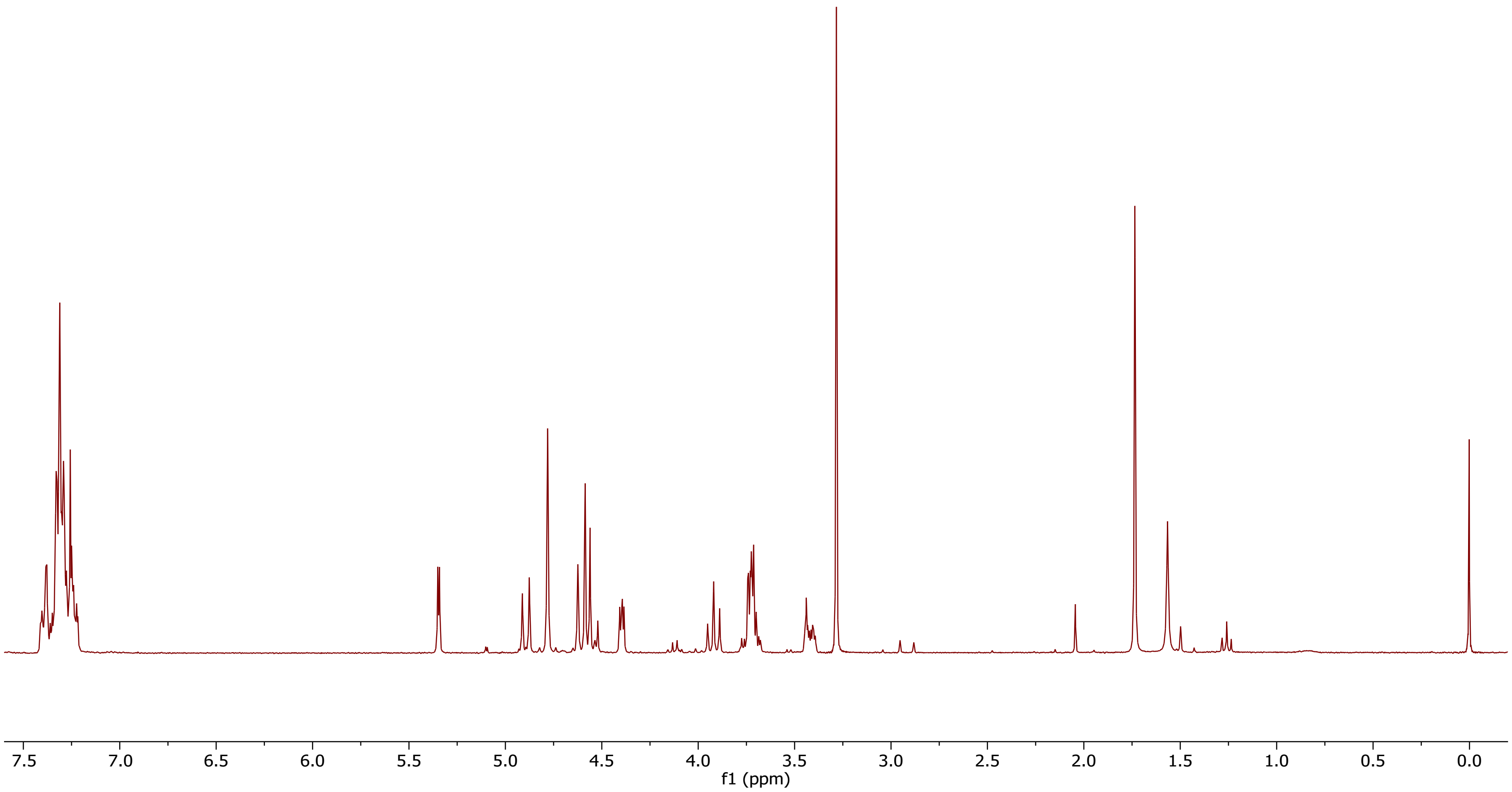

^{1}H NMR spectrum of 3,4,6-tri-*O*-benzyl-1,2-*O*-(1-methoxy-ethylidene)-β-D-[$^{13}C_6$]mannopyranose (**1**).

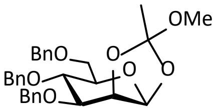

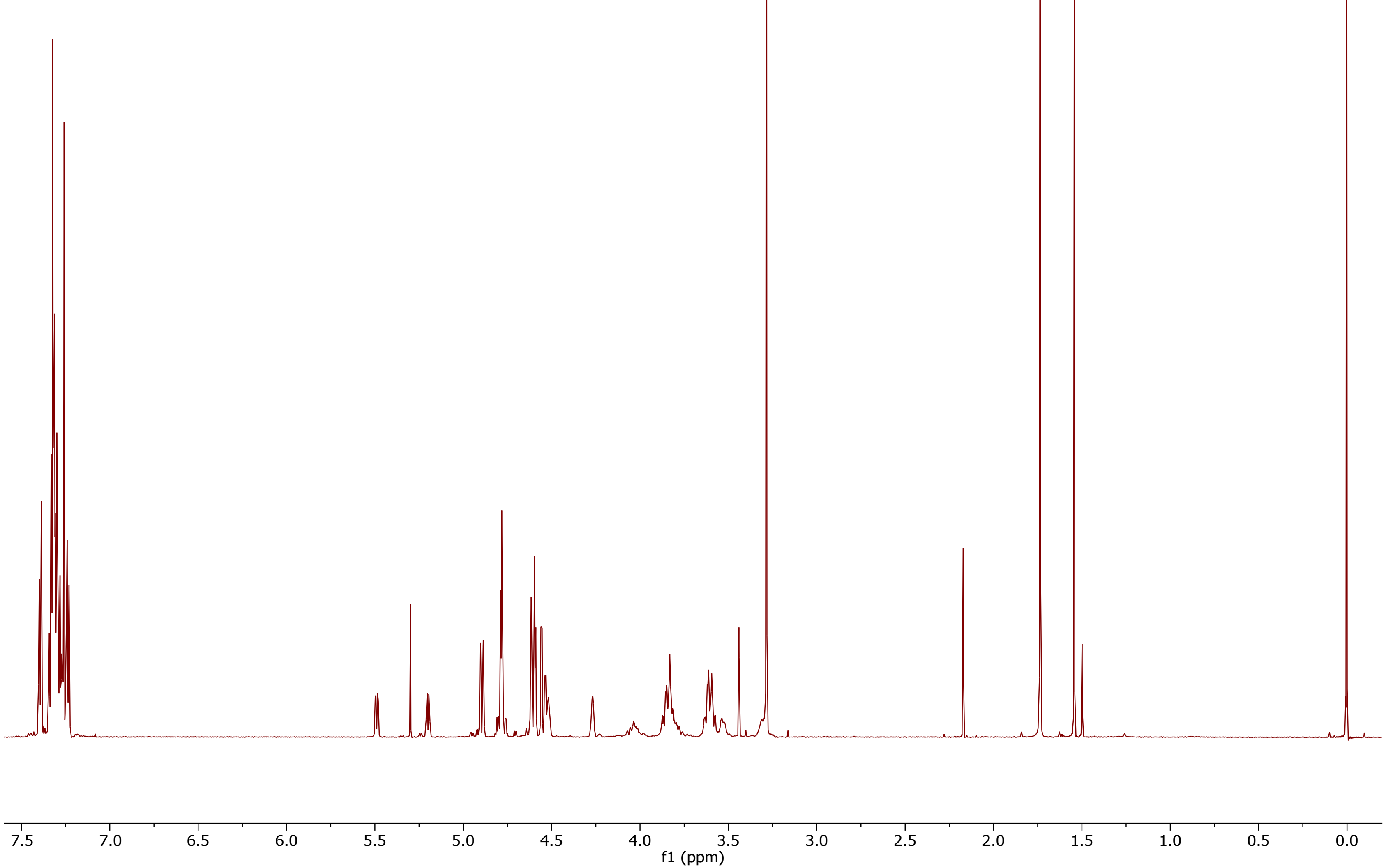

^{13}C NMR spectrum of 3,4,6-tri-*O*-benzyl-1,2-*O*-(1-methoxy-ethylidene)-β-D-[$^{13}C_6$]mannopyranose (1).

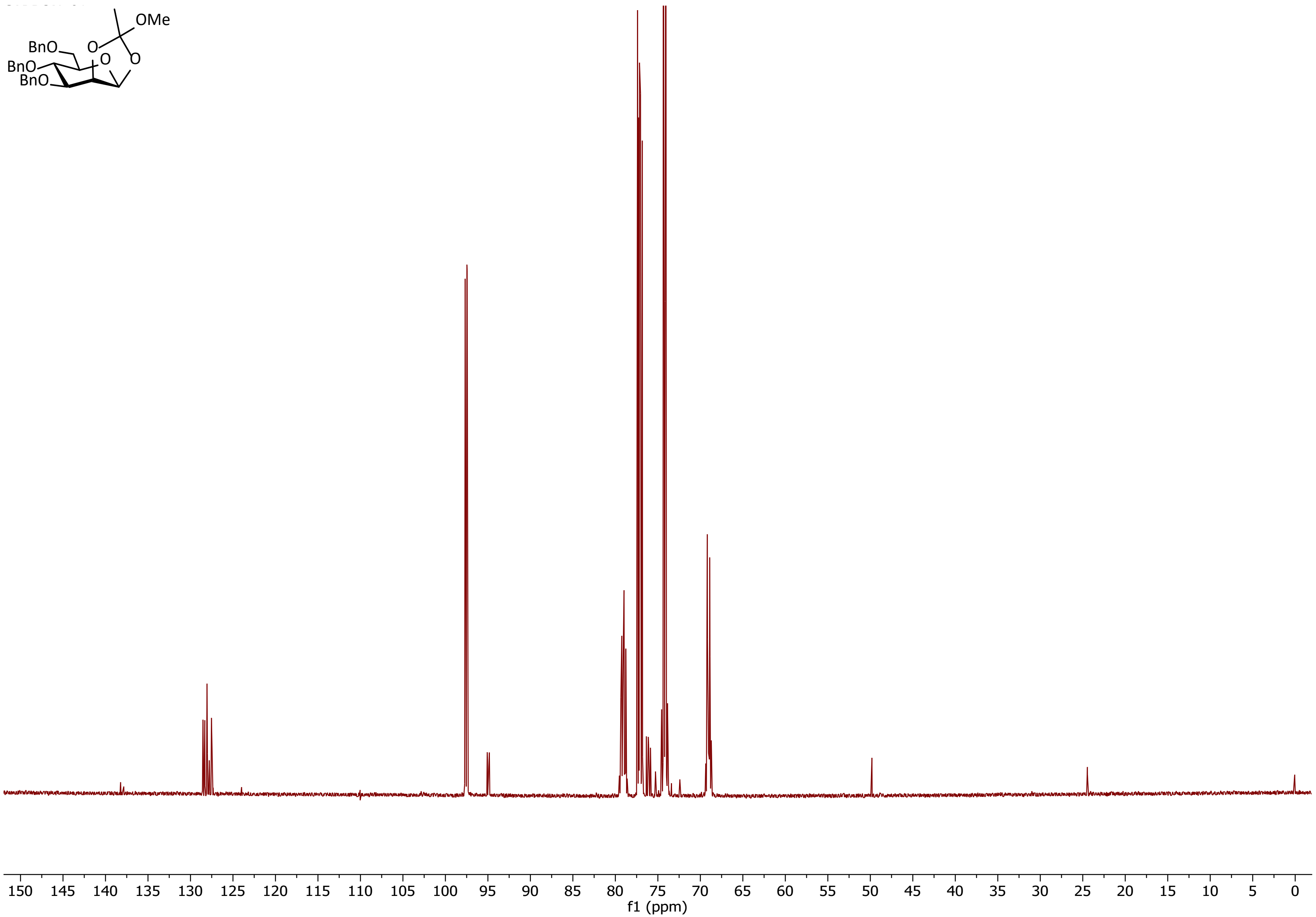

^{1}H NMR (^{13}C decoupled) spectrum of 2-*O*-acetyl-3,4,6-*O*-benzyl-α-D-[$^{13}C_6$]mannopyranosyl chloride (2).

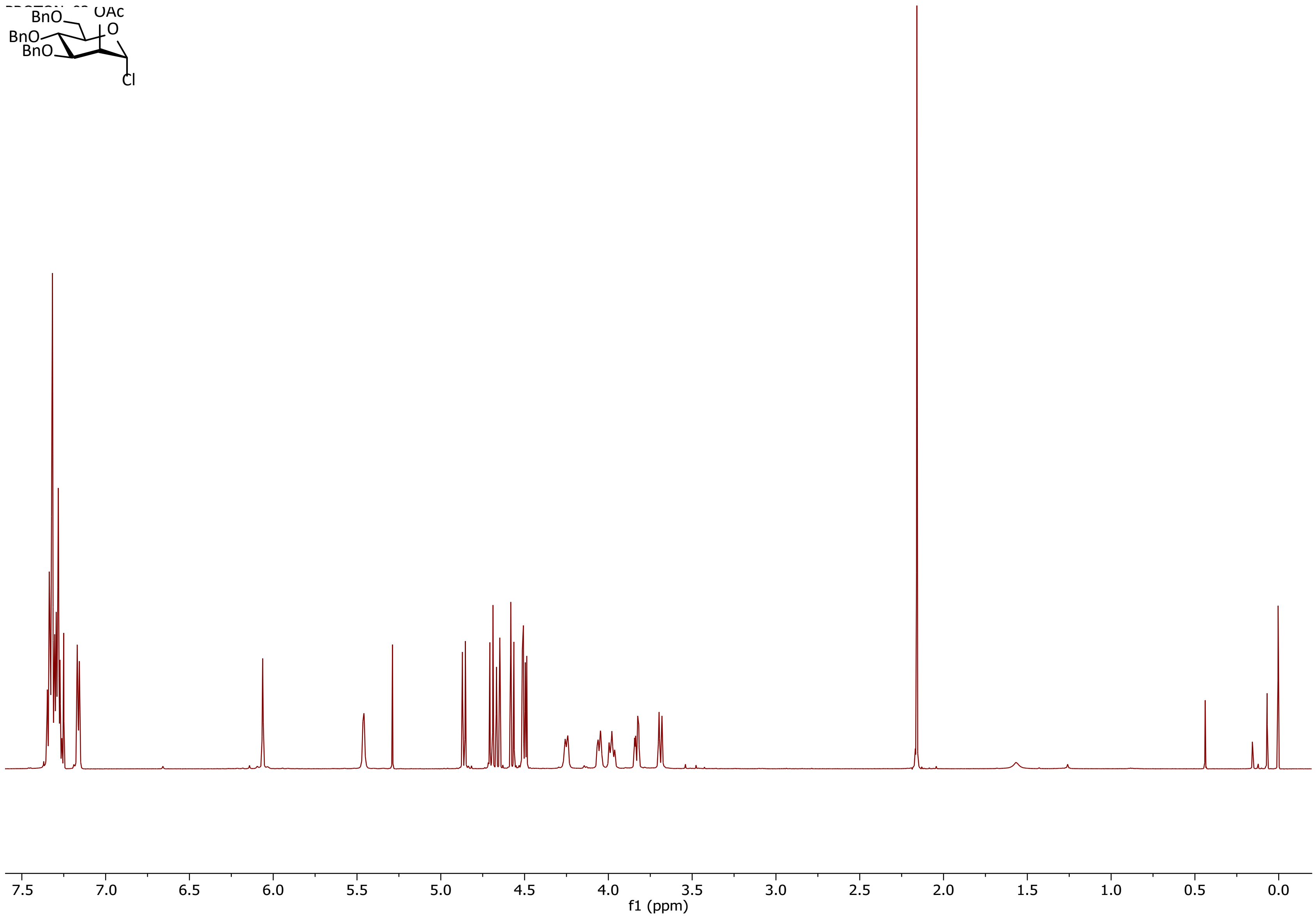

^{1}H NMR spectrum of 2-*O*-acetyl-3,4,6-*O*-benzyl-α-D-[$^{13}C_6$]mannopyranosyl chloride (2).

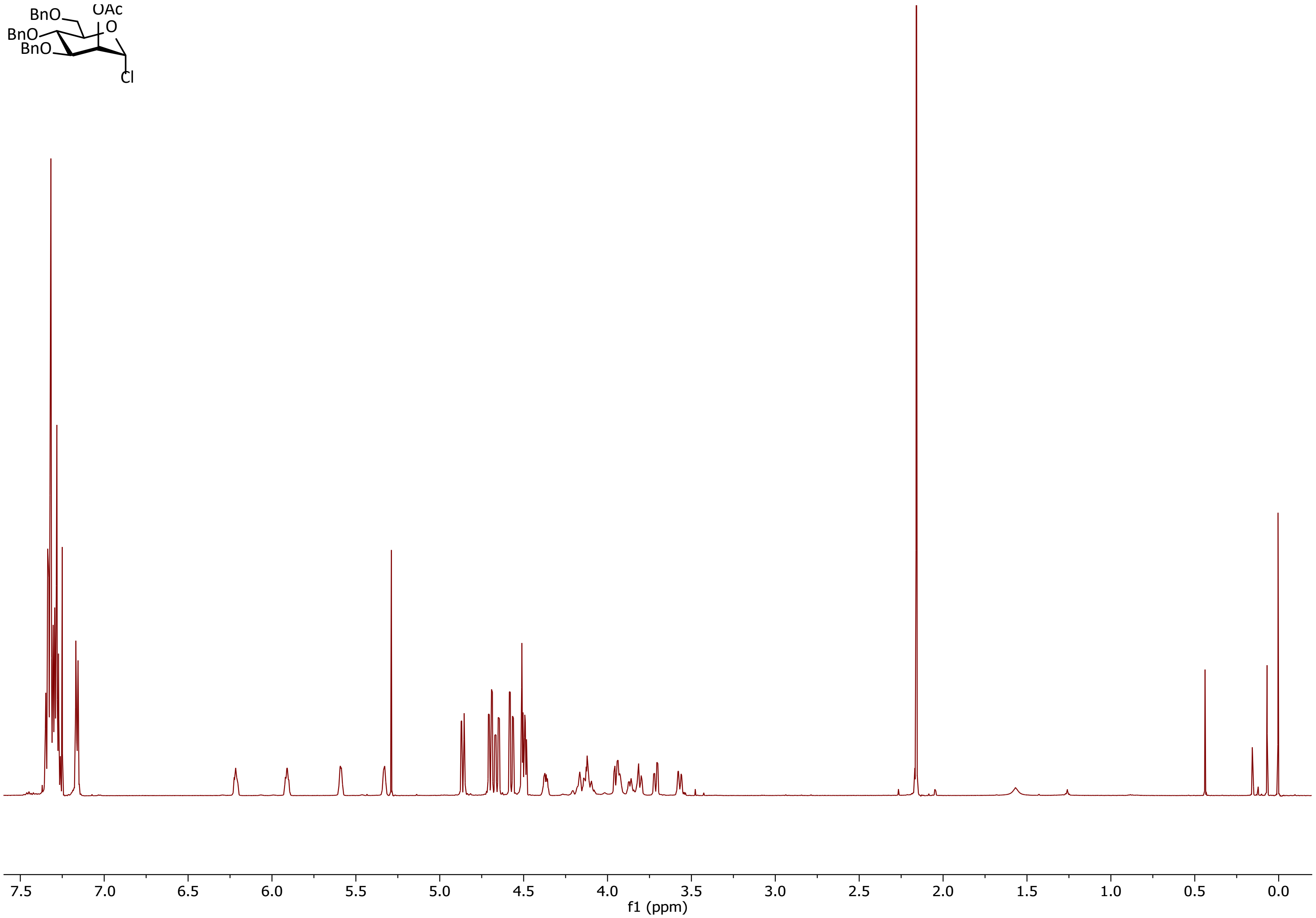

^{13}C NMR spectrum of 2-*O*-acetyl-3,4,6-*O*-benzyl-α-D-[$^{13}C_6$]mannopyranosyl chloride (2).

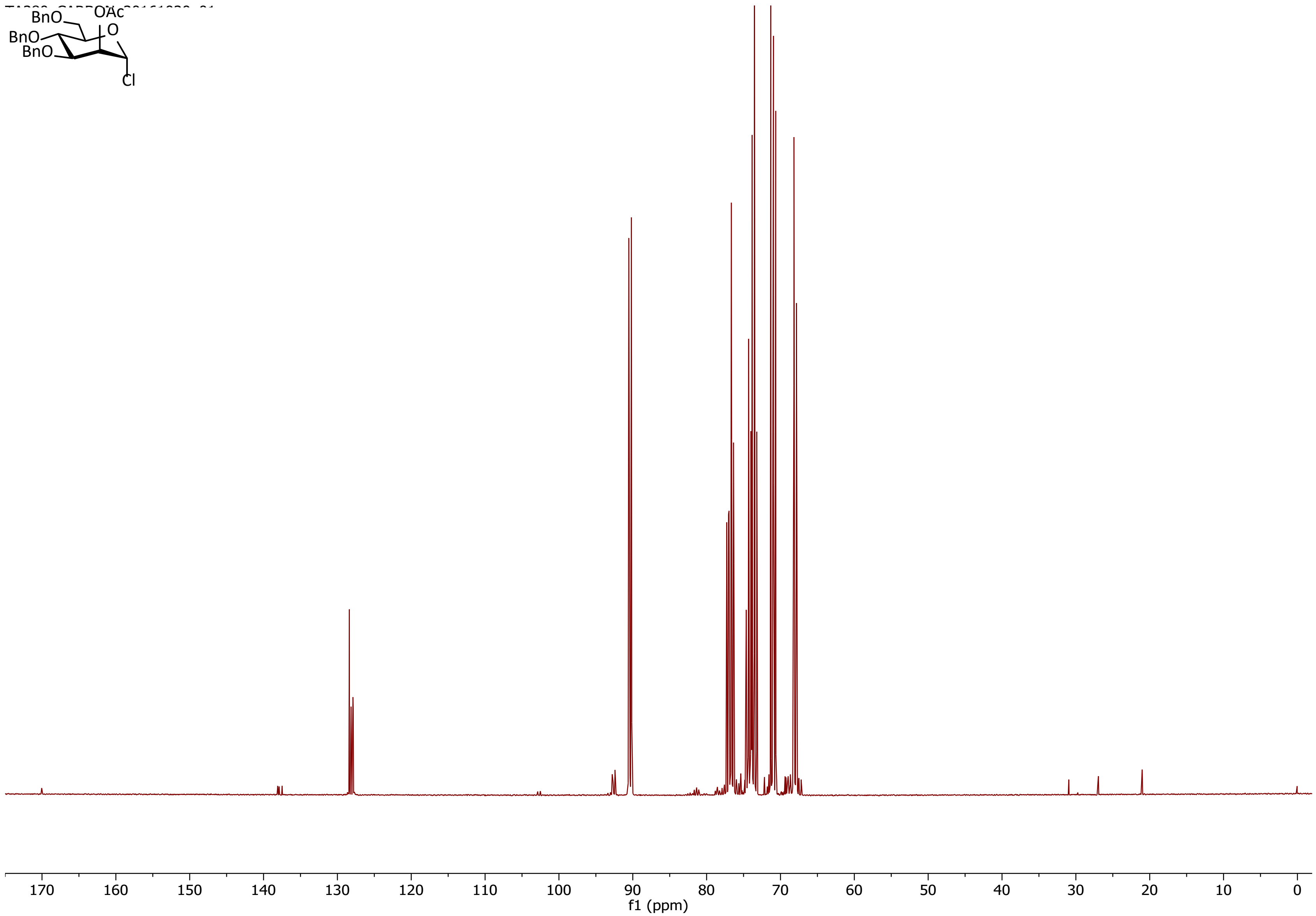

^{1}H NMR (^{13}C decoupled) spectrum of Methyl 3,4,6-tri-*O*-benzyl-α-D-[$^{13}C_6$]mannopyranoside (**3**).

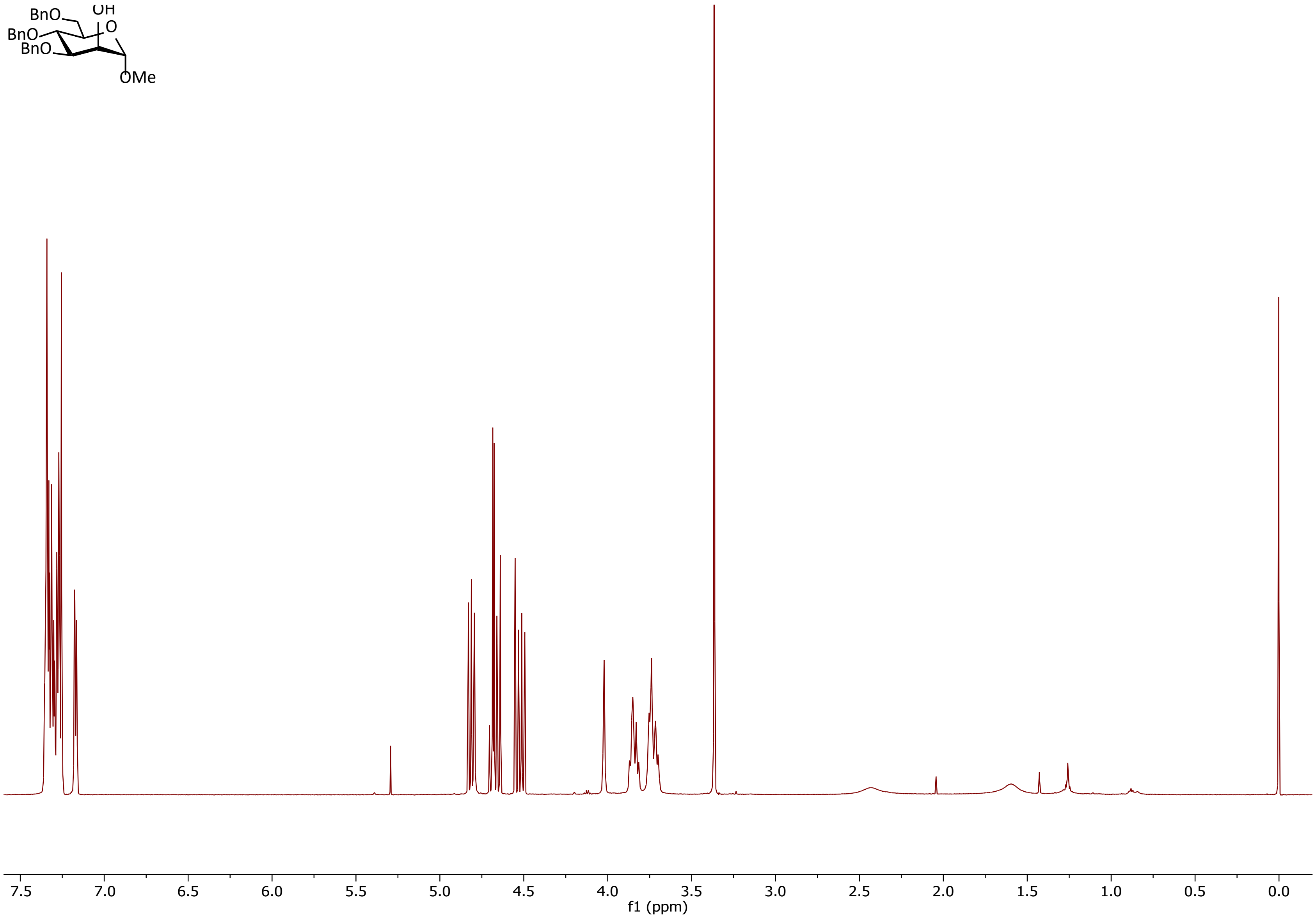

^{1}H NMR spectrum of Methyl 3,4,6-tri-*O*-benzyl-α-D-[$^{13}C_6$]mannopyranoside (**3**).

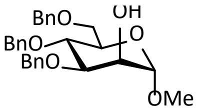

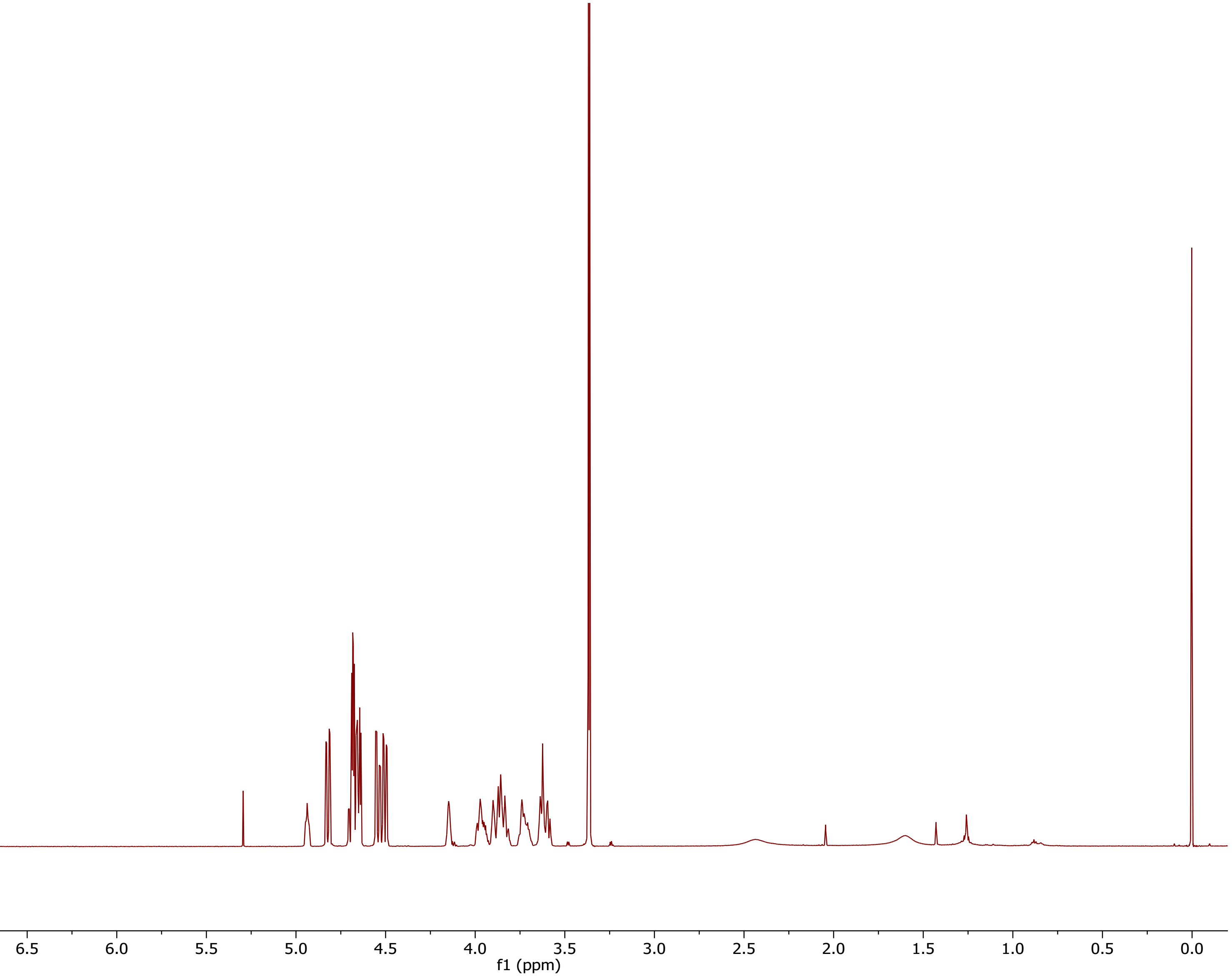

^{13}C NMR spectrum of Methyl 3,4,6-tri-*O*-benzyl-α-D-[$^{13}C_6$]mannopyranoside (3).

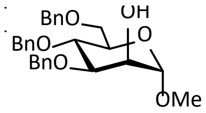

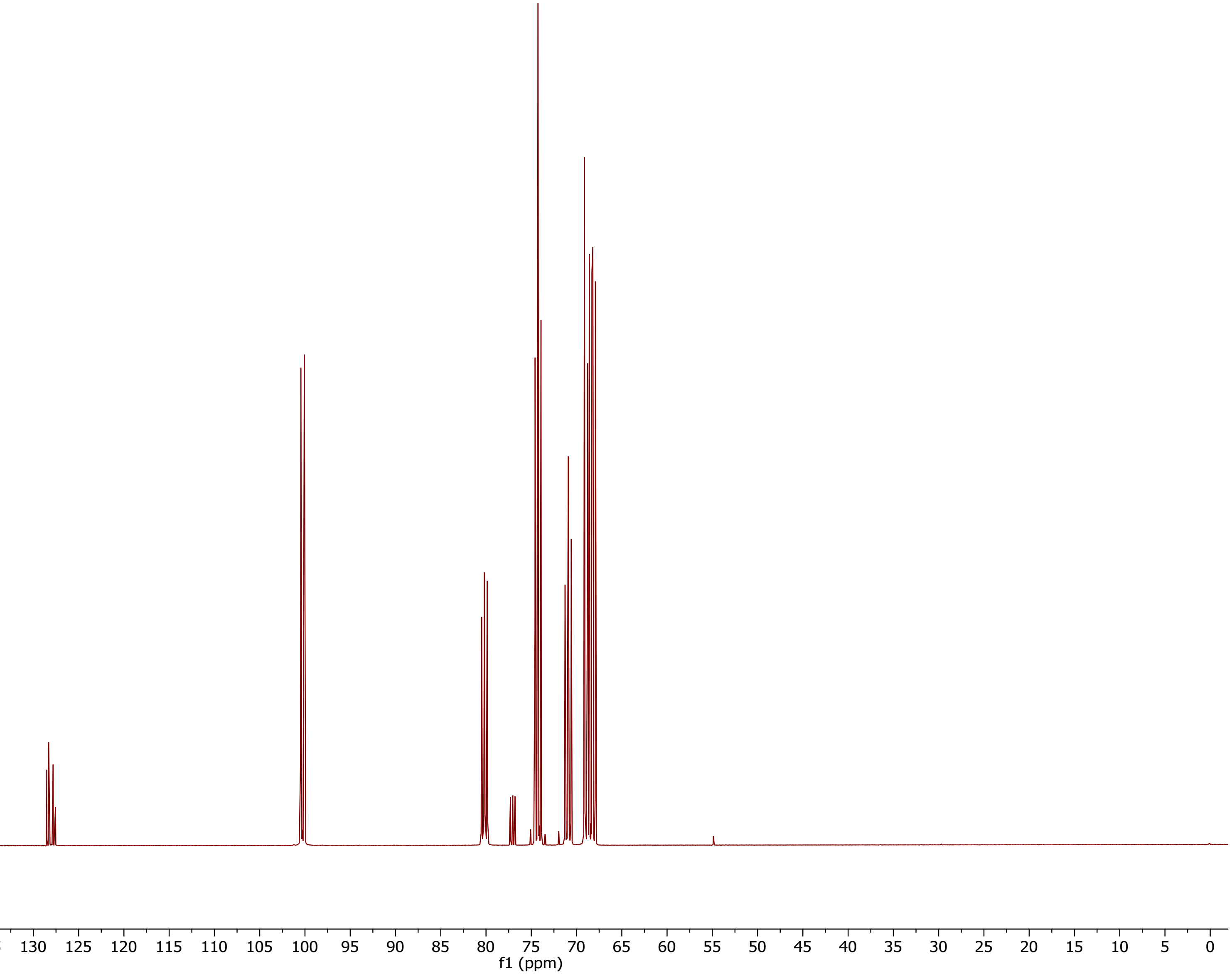

Coupled HSQC spectrum of Methyl 3,4,6-tri-*O*-benzyl-α-D-[$^{13}C_6$]mannopyranoside (3).

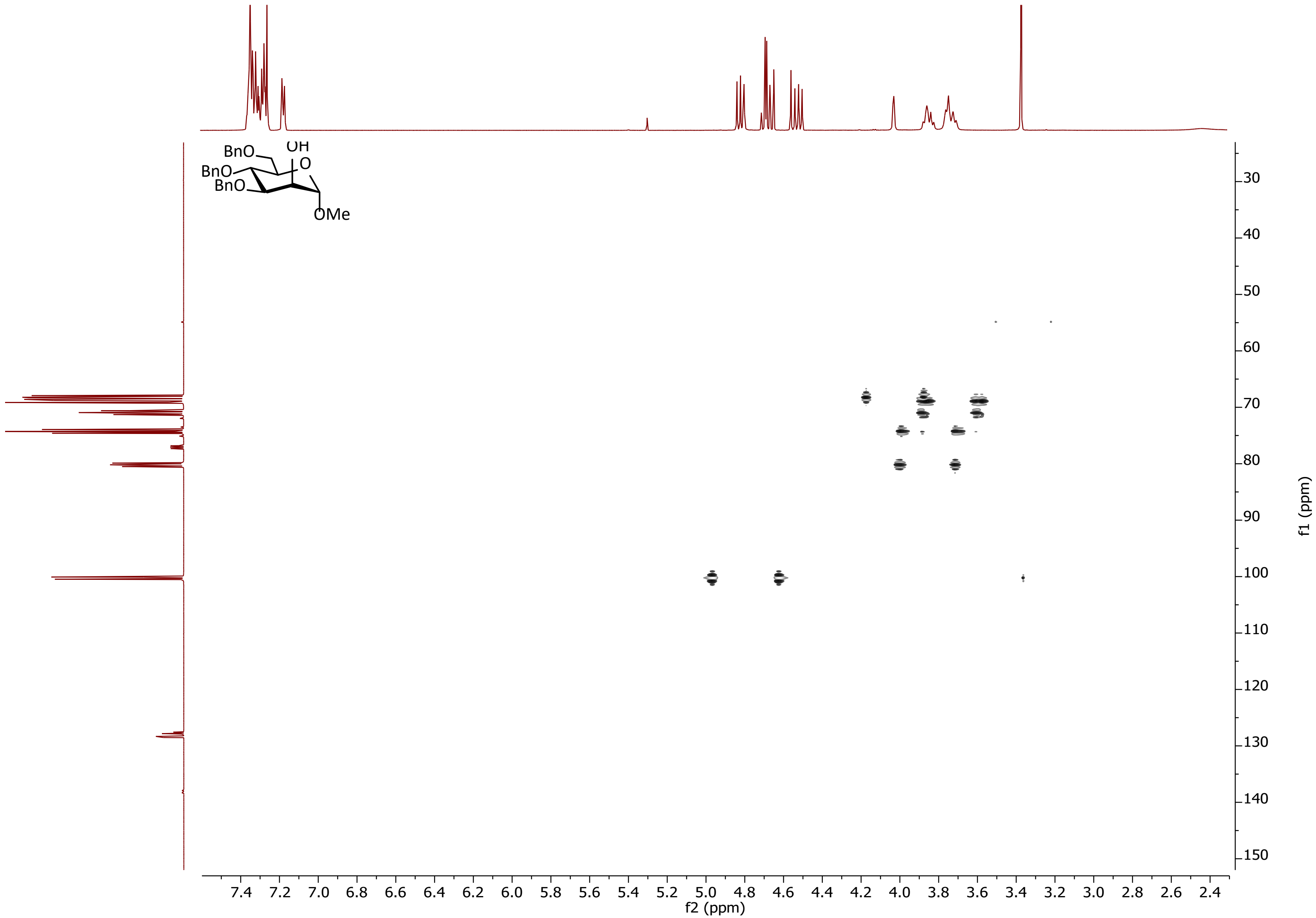

^{1}H NMR (^{13}C decoupled) spectrum of Methyl 2-*O*-acetyl-3,4,6-tri-*O*-benzyl-α-D-[$^{13}C_6$]mannopyranosyl-(1→2)-3,4,6-tri-*O*-benzyl-α-D-[$^{13}C_6$]mannopyranoside (4).

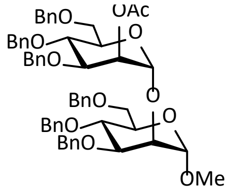

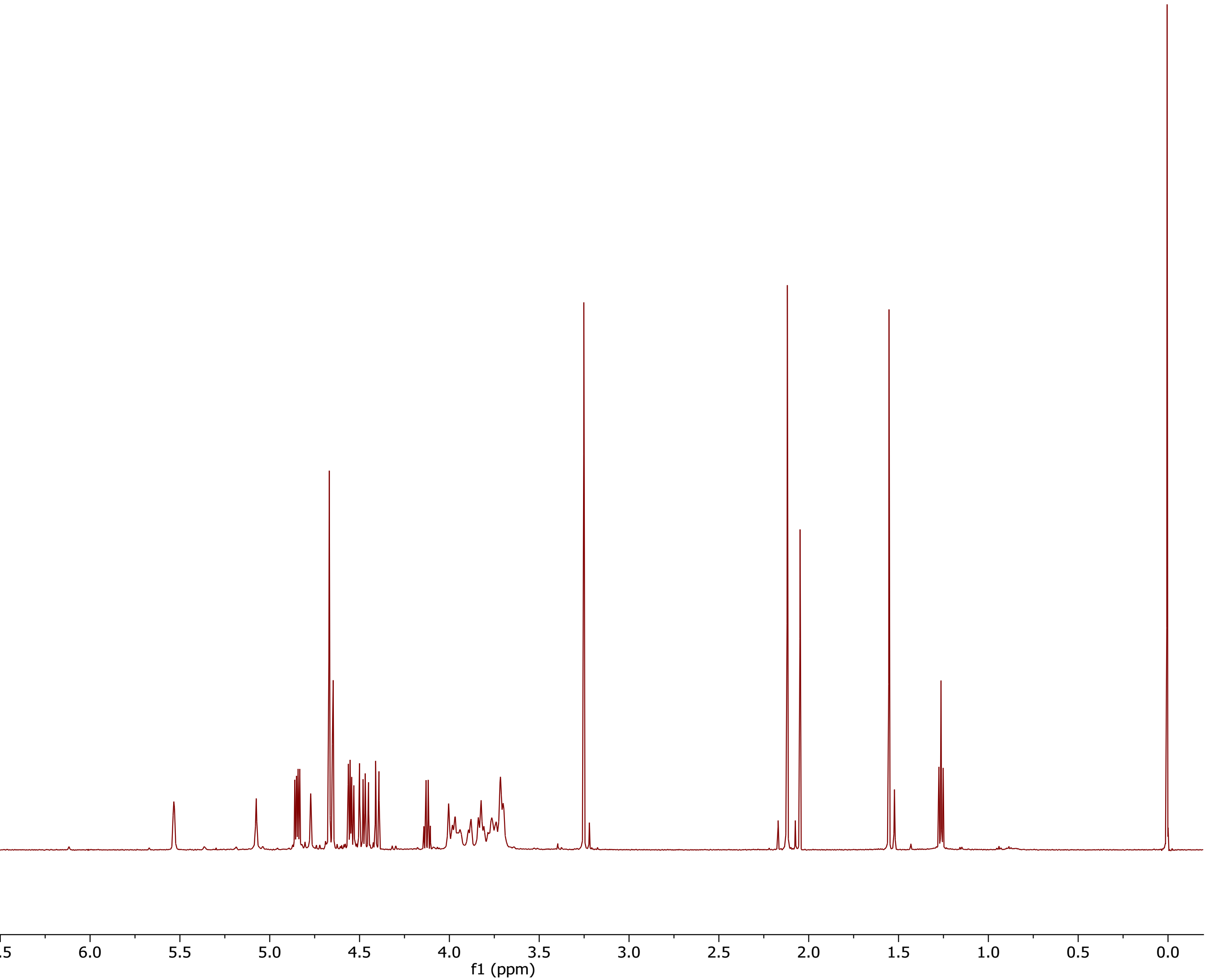

^{1}H NMR spectrum of Methyl 2-*O*-acetyl-3,4,6-tri-*O*-benzyl-α-D-[$^{13}C_6$]mannopyranosyl-(1→2)-3,4,6-tri-*O*-benzyl-α-D-[$^{13}C_6$]mannopyranoside (**4**).

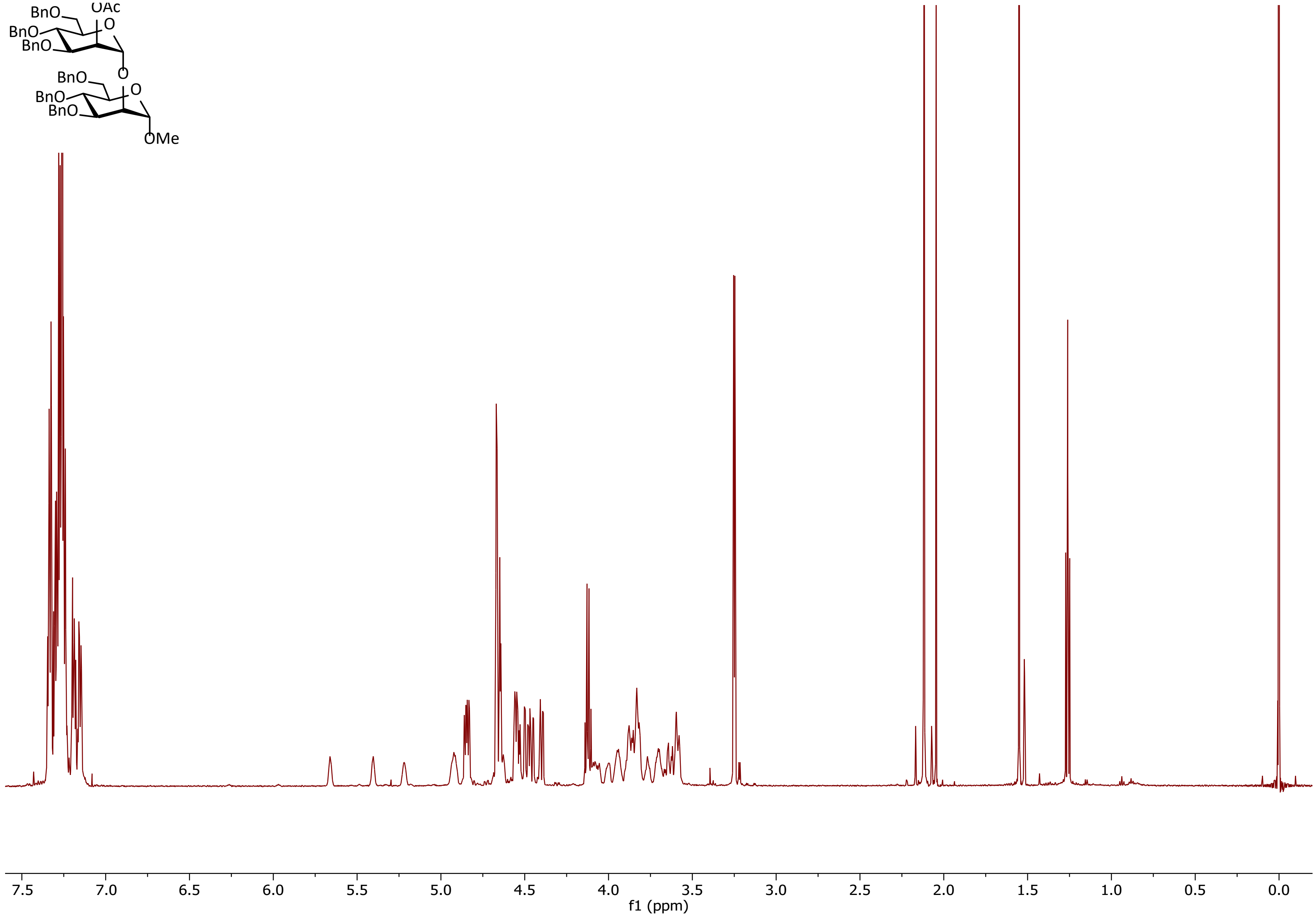

^{13}C NMR spectrum of Methyl 2-*O*-acetyl-3,4,6-tri-*O*-benzyl-α-D-[$^{13}C_6$]mannopyranosyl-(1→2)-3,4,6-tri-*O*-benzyl-α-D-[$^{13}C_6$]mannopyranoside (**4**).

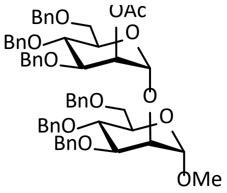

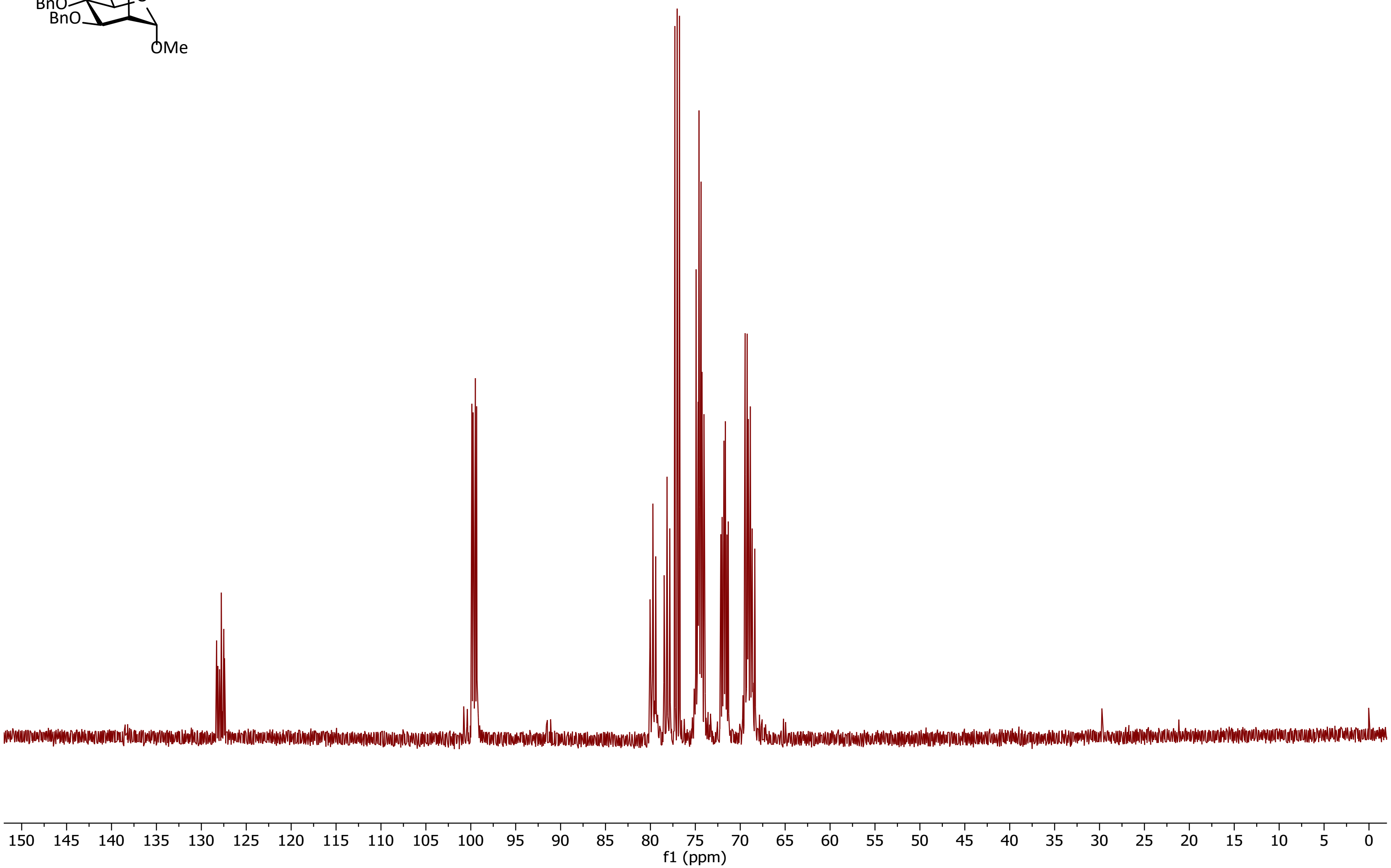

Coupled HSQC spectrum of Methyl 2-*O*-acetyl-3,4,6-tri-*O*-benzyl-α-D-[$^{13}C_6$]mannopyranosyl-(1→2)-3,4,6-tri-*O*-benzyl-α-D-[$^{13}C_6$]mannopyranoside (4).

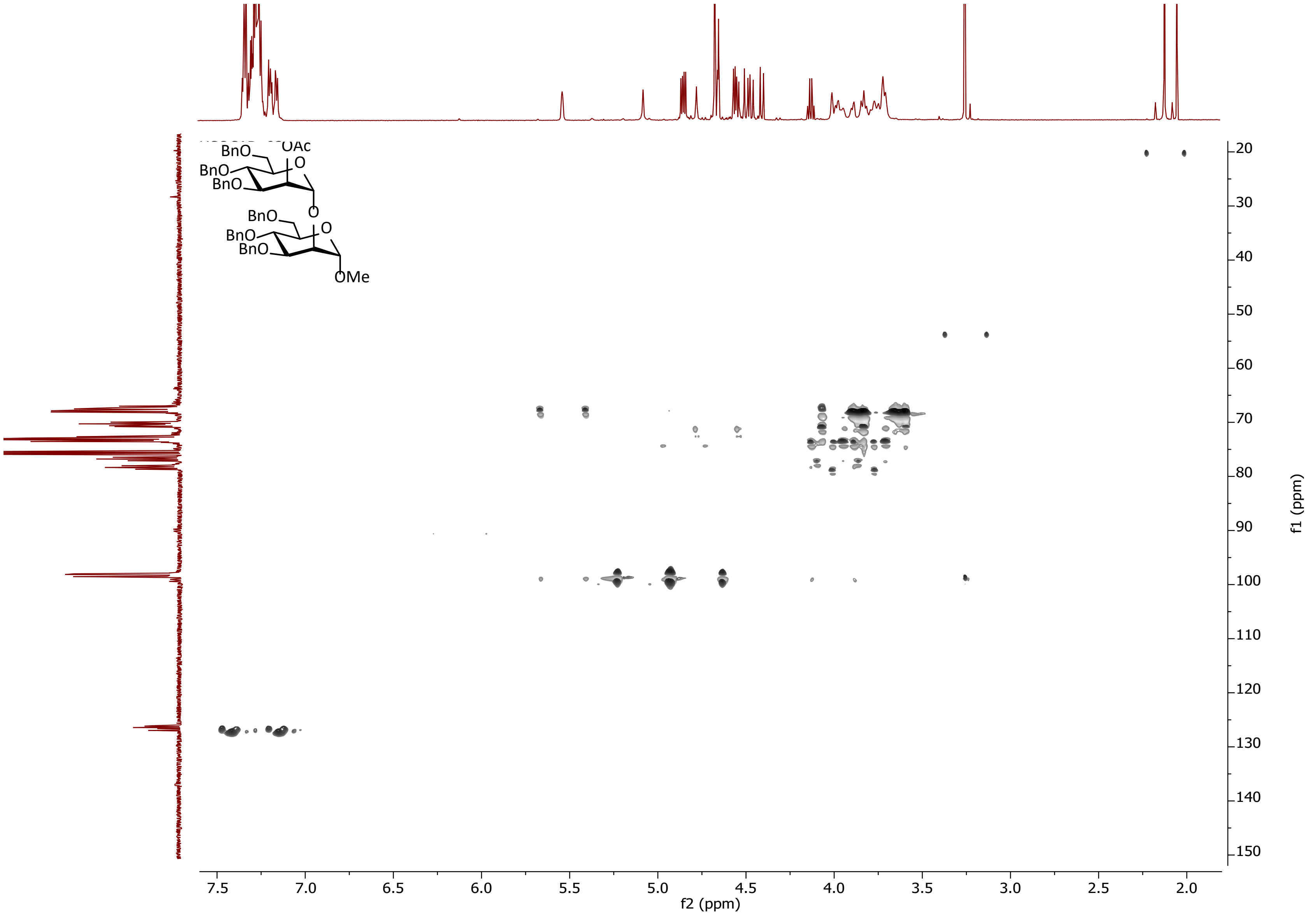

^{1}H NMR (^{13}C decoupled) spectrum of Methyl 3,4,6-tri-*O*-benzyl-α-D-[$^{13}C_6$]mannopyranosyl-(1→2)-3,4,6-tri-*O*-benzyl-α-D-[$^{13}C_6$]mannopyranoside (**5**).

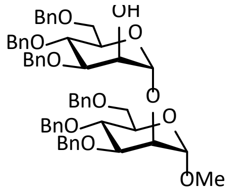

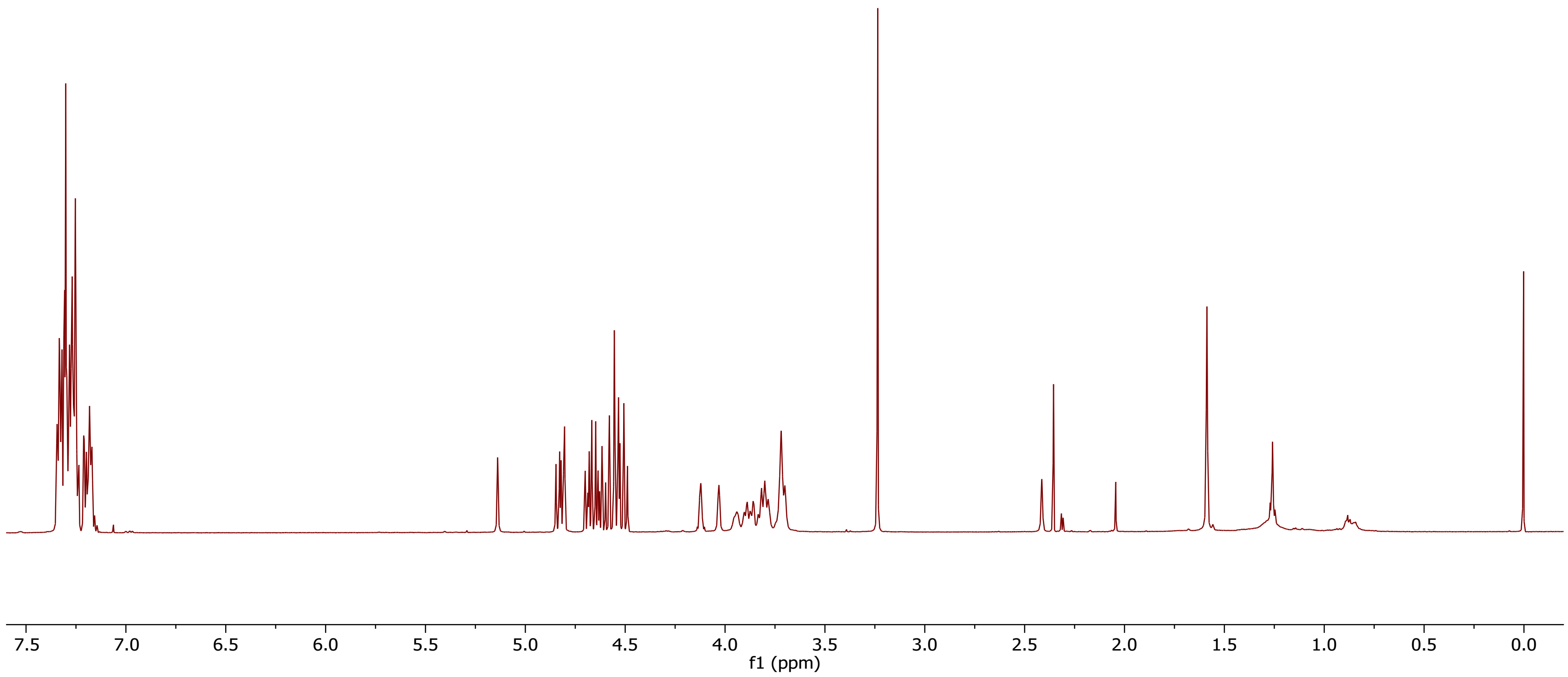

^{1}H NMR spectrum of Methyl 3,4,6-tri-*O*-benzyl-α-D-[$^{13}C_6$]mannopyranosyl-(1→2)-3,4,6-tri-*O*-benzyl-α-D-[$^{13}C_6$]mannopyranoside (**5**).

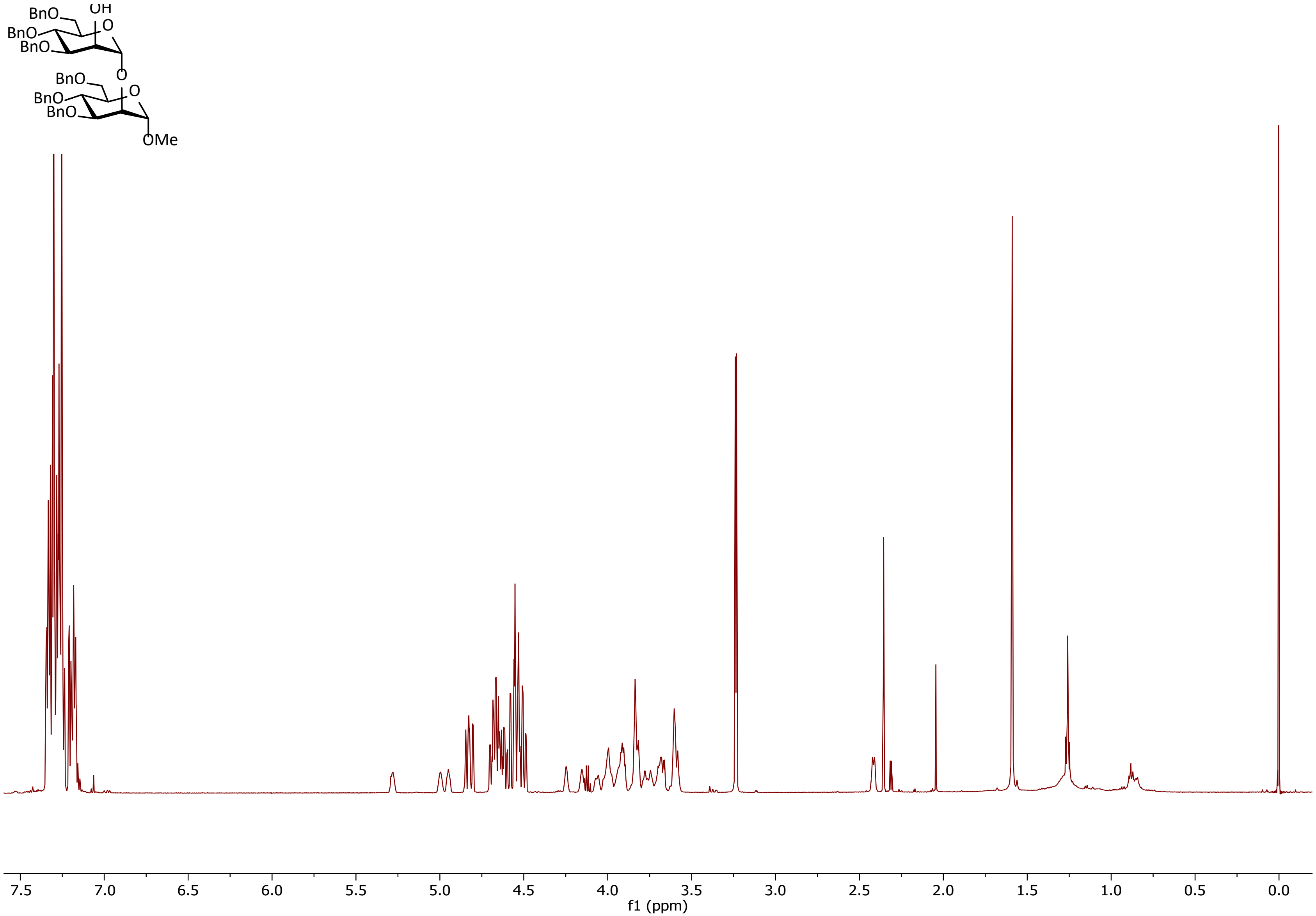

^{13}C NMR spectrum of Methyl 3,4,6-tri-*O*-benzyl-α-D-[$^{13}C_6$]mannopyranosyl-(1→2)-3,4,6-tri-*O*-benzyl-α-D-[$^{13}C_6$]mannopyranoside (**5**).

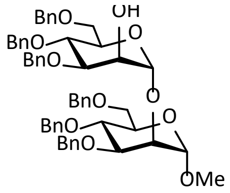

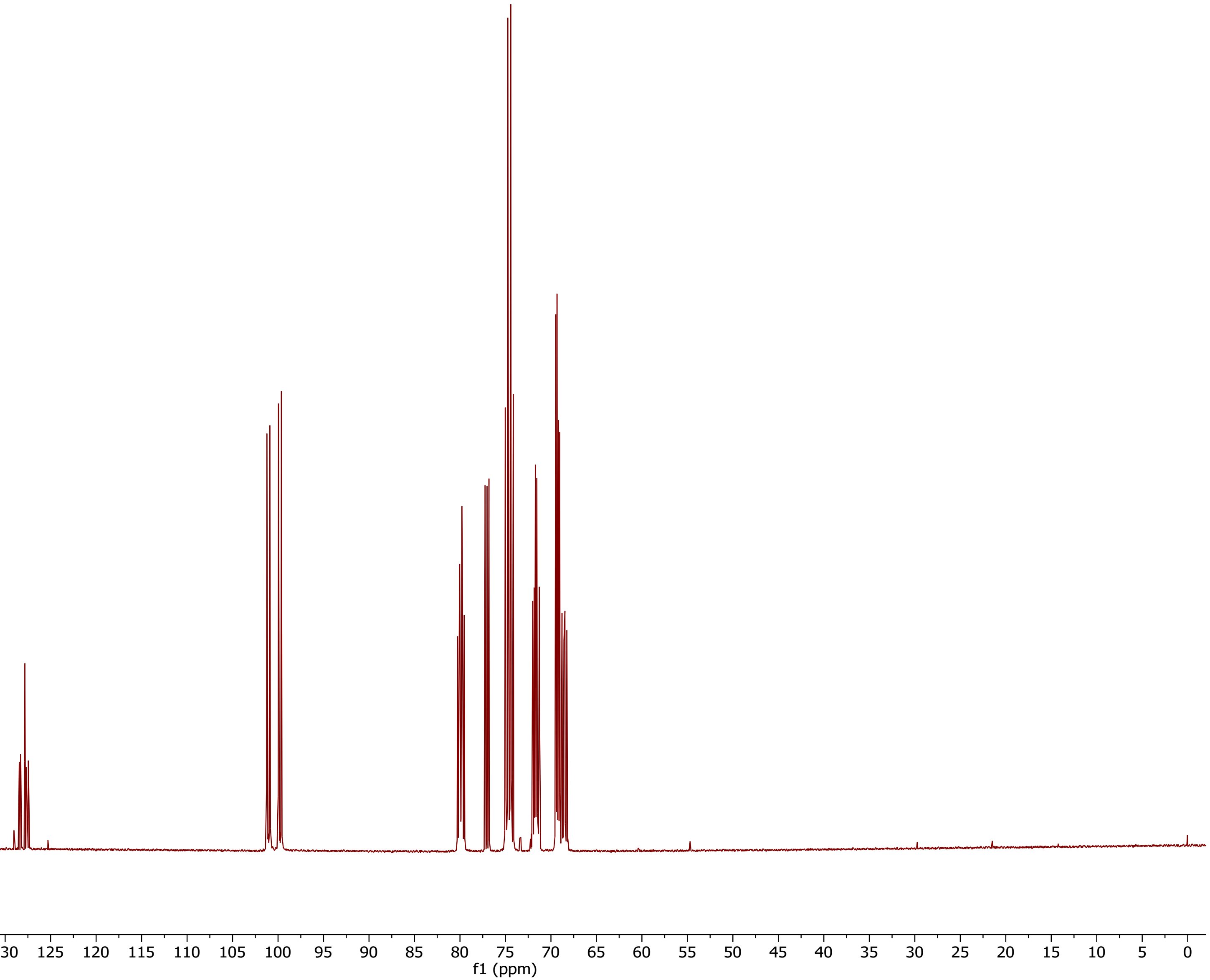

^{1}H NMR spectrum of Methyl α-D-mannopyranosyl-(1→2)-α-D-mannopyranoside (6).*

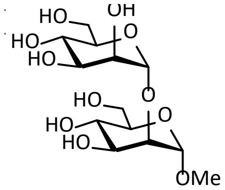

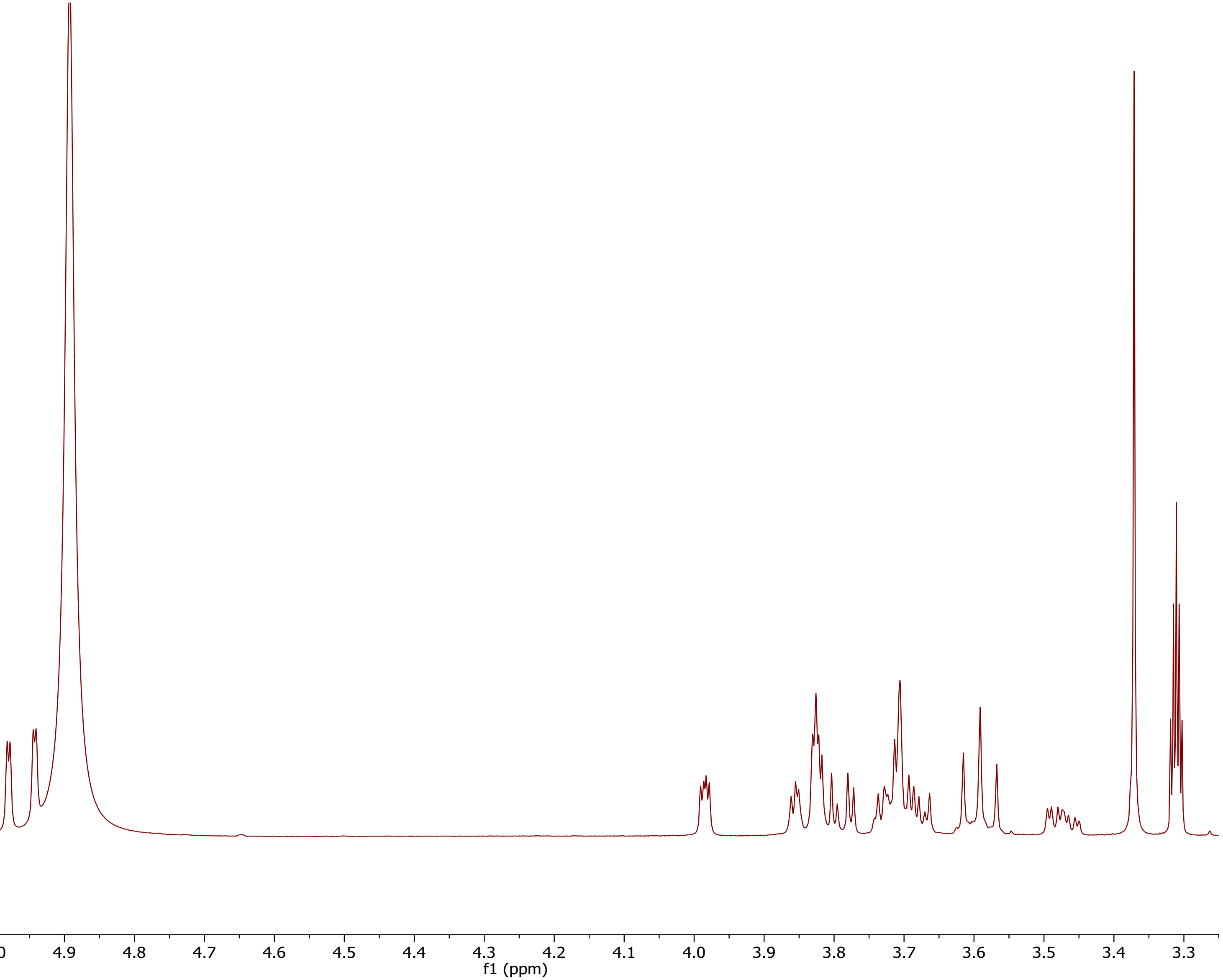

^{1}H NMR spectrum of Methyl α-D-[[13]C_6]mannopyranosyl-(1→2)-α-D-[[13]C_6]mannopyranoside (**6**).

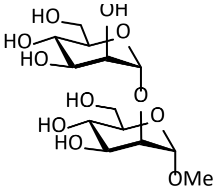

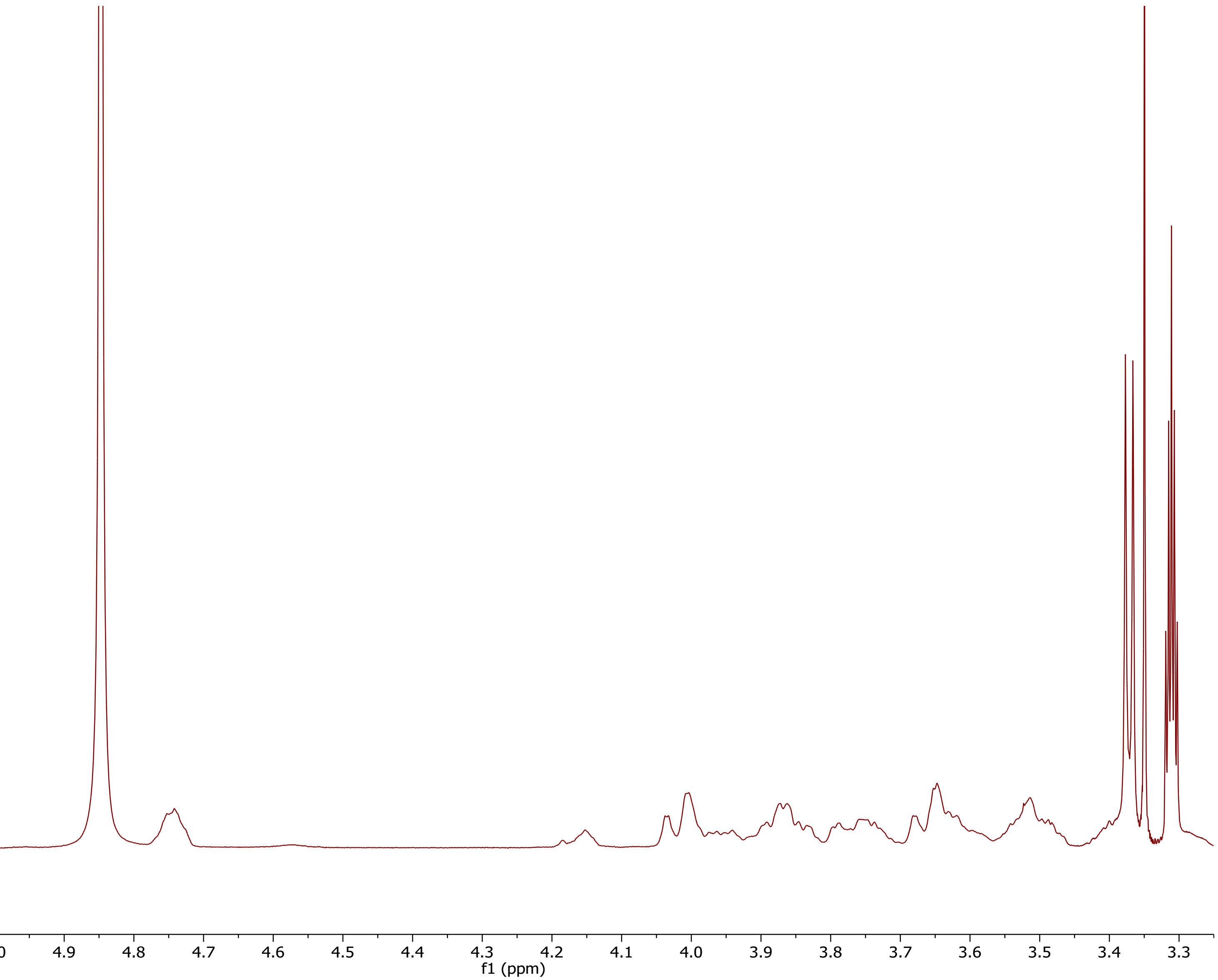

^{13}C NMR spectrum of Methyl α-D-[$^{13}C_6$]mannopyranosyl-(1→2)-α-D-[$^{13}C_6$]mannopyranoside (**6**).

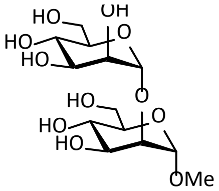

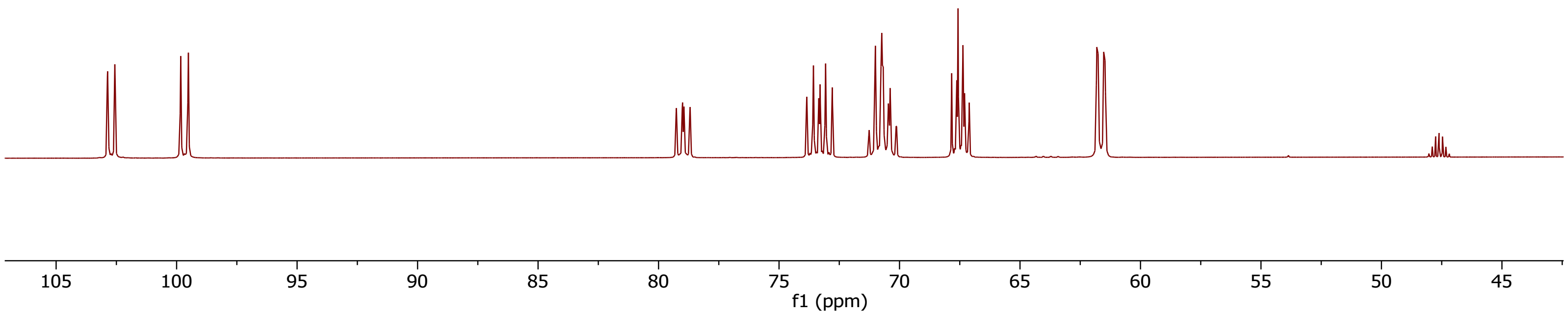

^{1}H NMR spectrum of Methyl 2,3,4-tri-O-benzyl-α-D-mannopyranoside (7).*

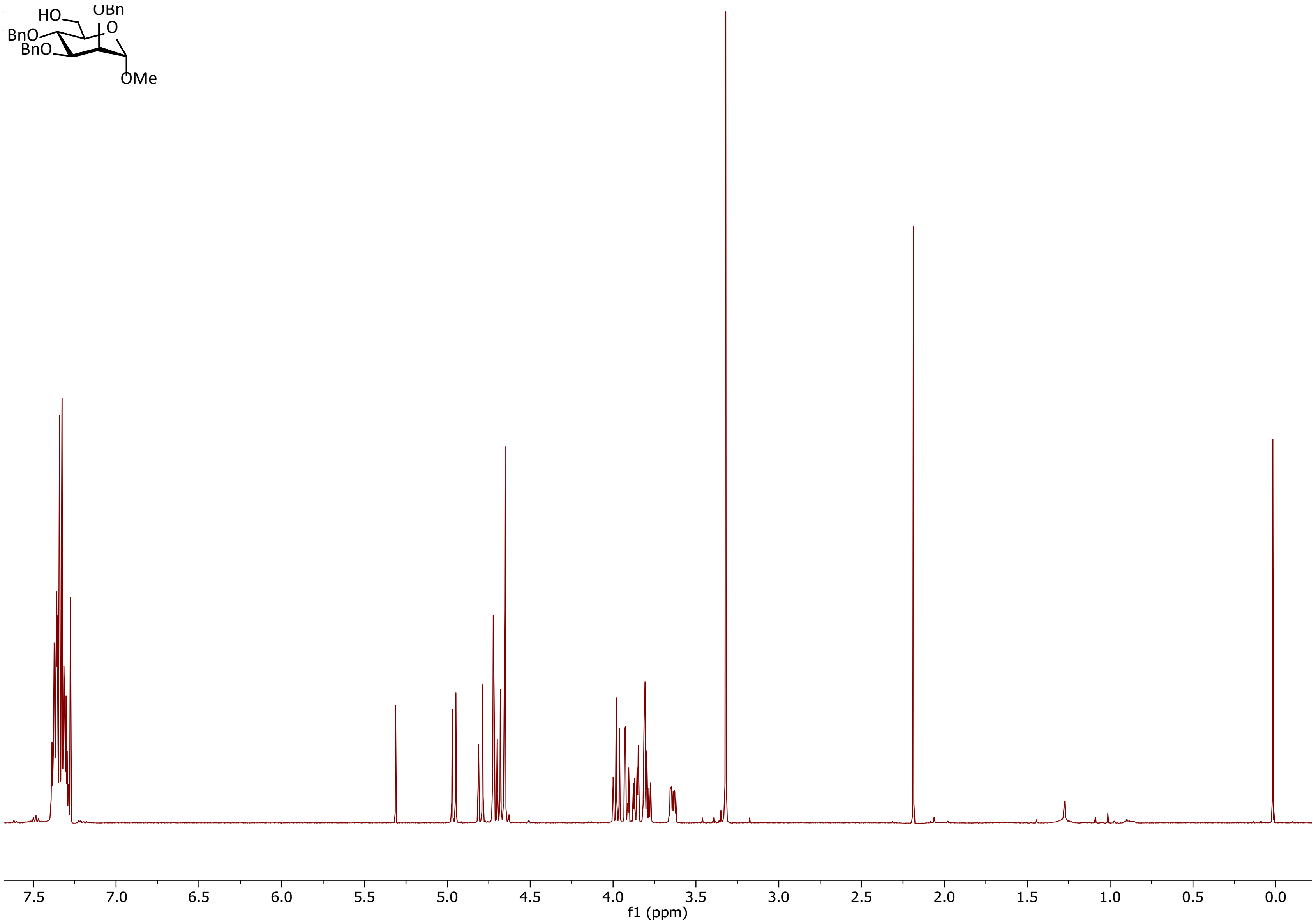

^{1}H NMR spectrum of Methyl 2,3,4-tri-O-benzyl-α-D-[$^{13}C_6$]mannopyranoside (**7**).

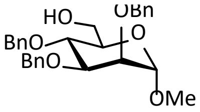

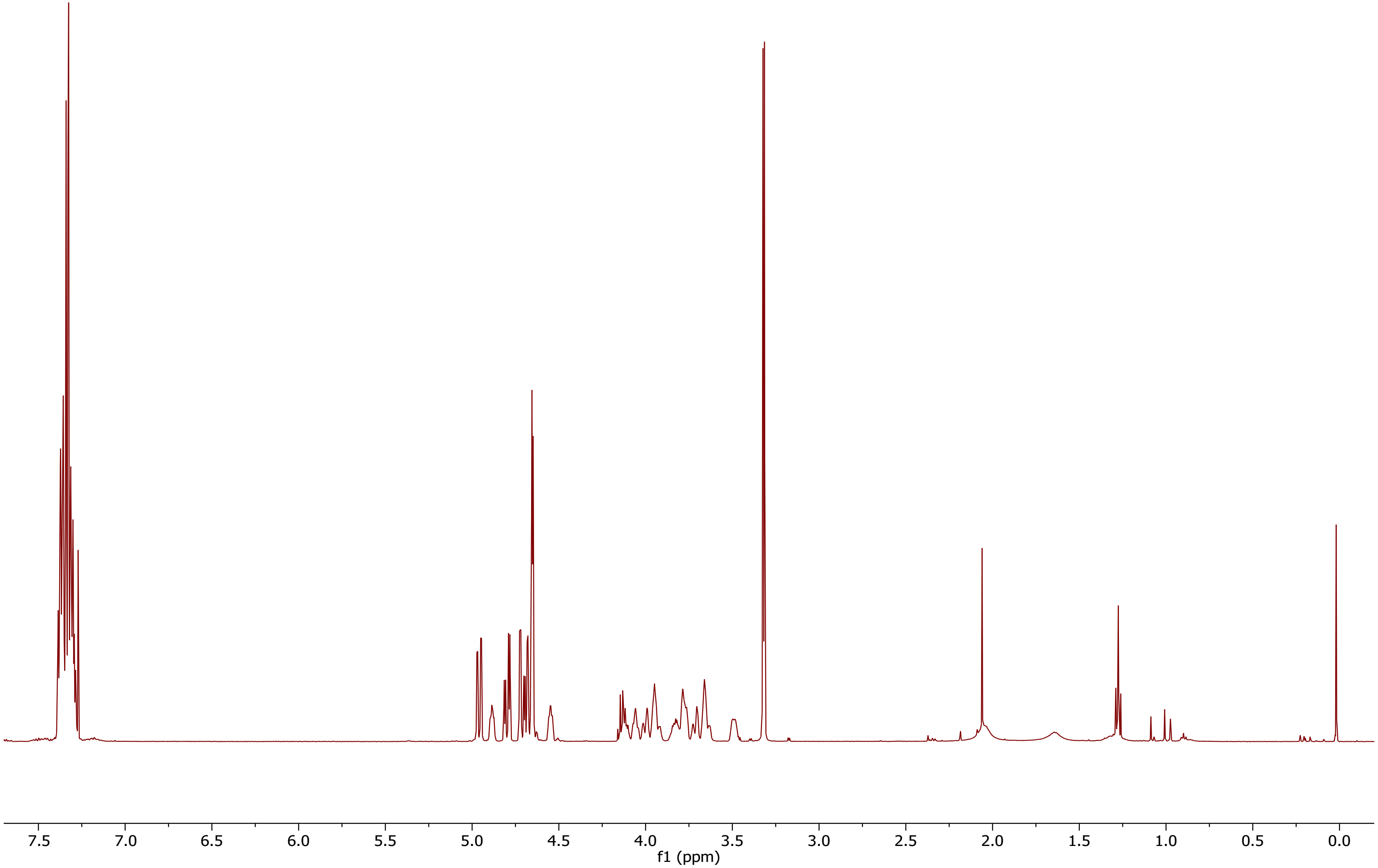

^{13}C NMR spectrum of Methyl 2,3,4-tri-O-benzyl-α-D-[$^{13}C_6$]mannopyranoside (7).

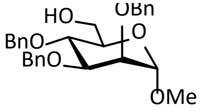

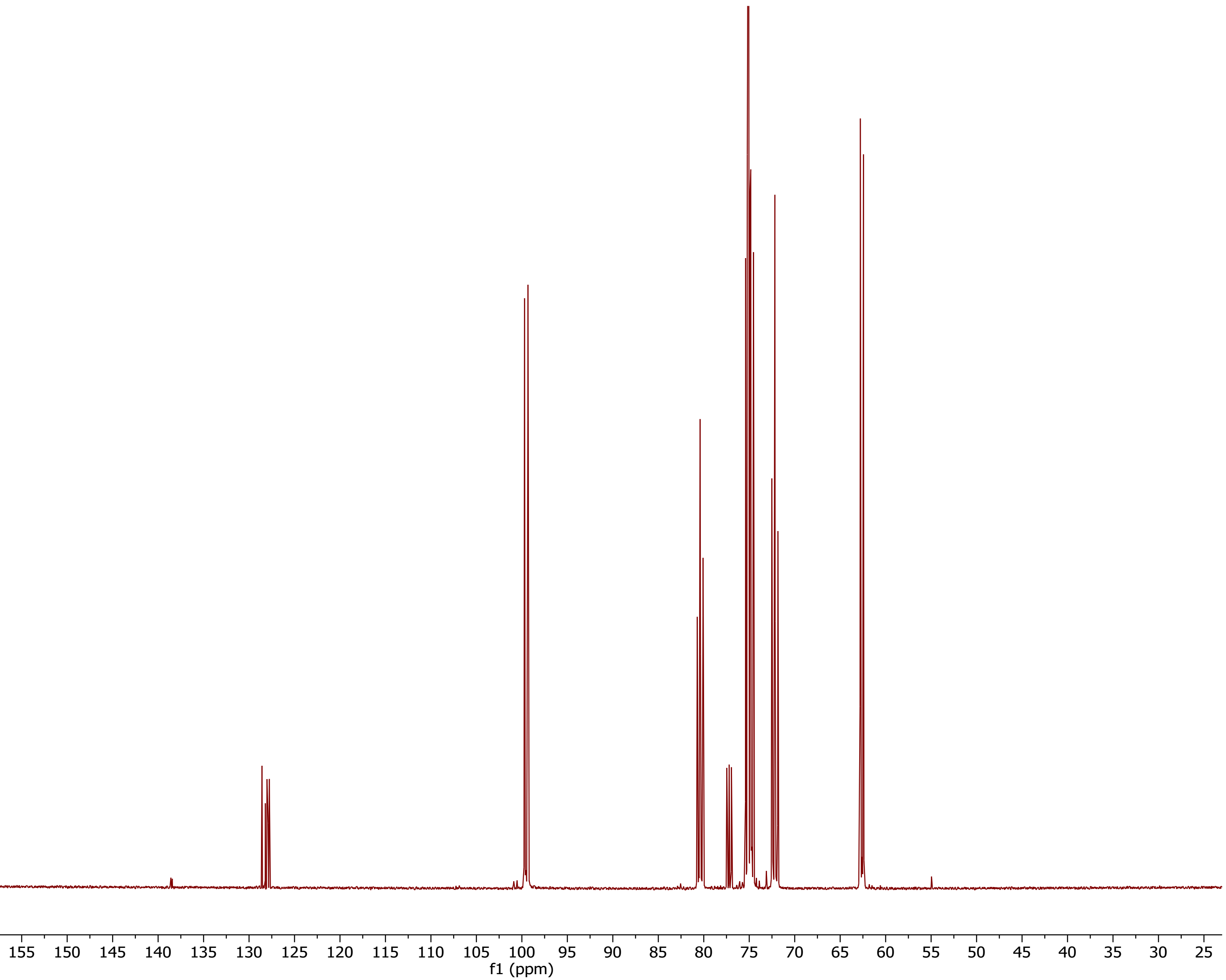

^{1}H NMR spectrum of Methyl 2-*O*-acetyl-3,4,6-tri-*O*-benzyl-α-D-mannopyranosyl-(1→6)-2,3,4-tri-*O*-benzyl-α-D-mannopyranoside (**8**).*

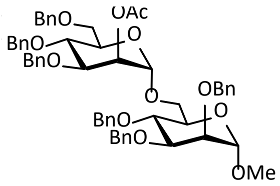

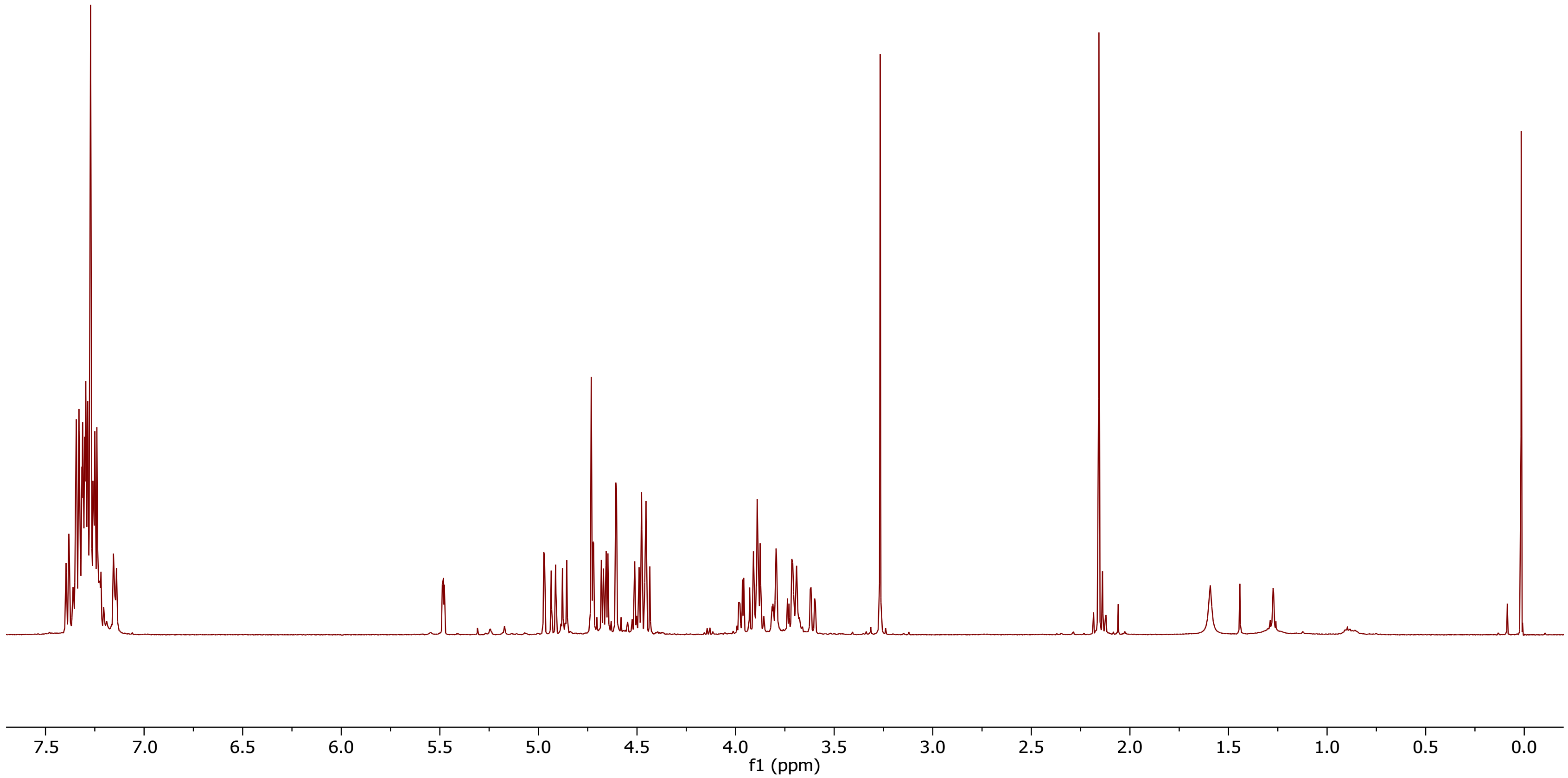

^{1}H NMR spectrum of Methyl 2-*O*-acetyl-3,4,6-tri-*O*-benzyl-α-D-[$^{13}C_6$]mannopyranosyl-(1→6)-2,3,4-tri-*O*-benzyl-α-D-[$^{13}C_6$]mannopyranoside (**8**).

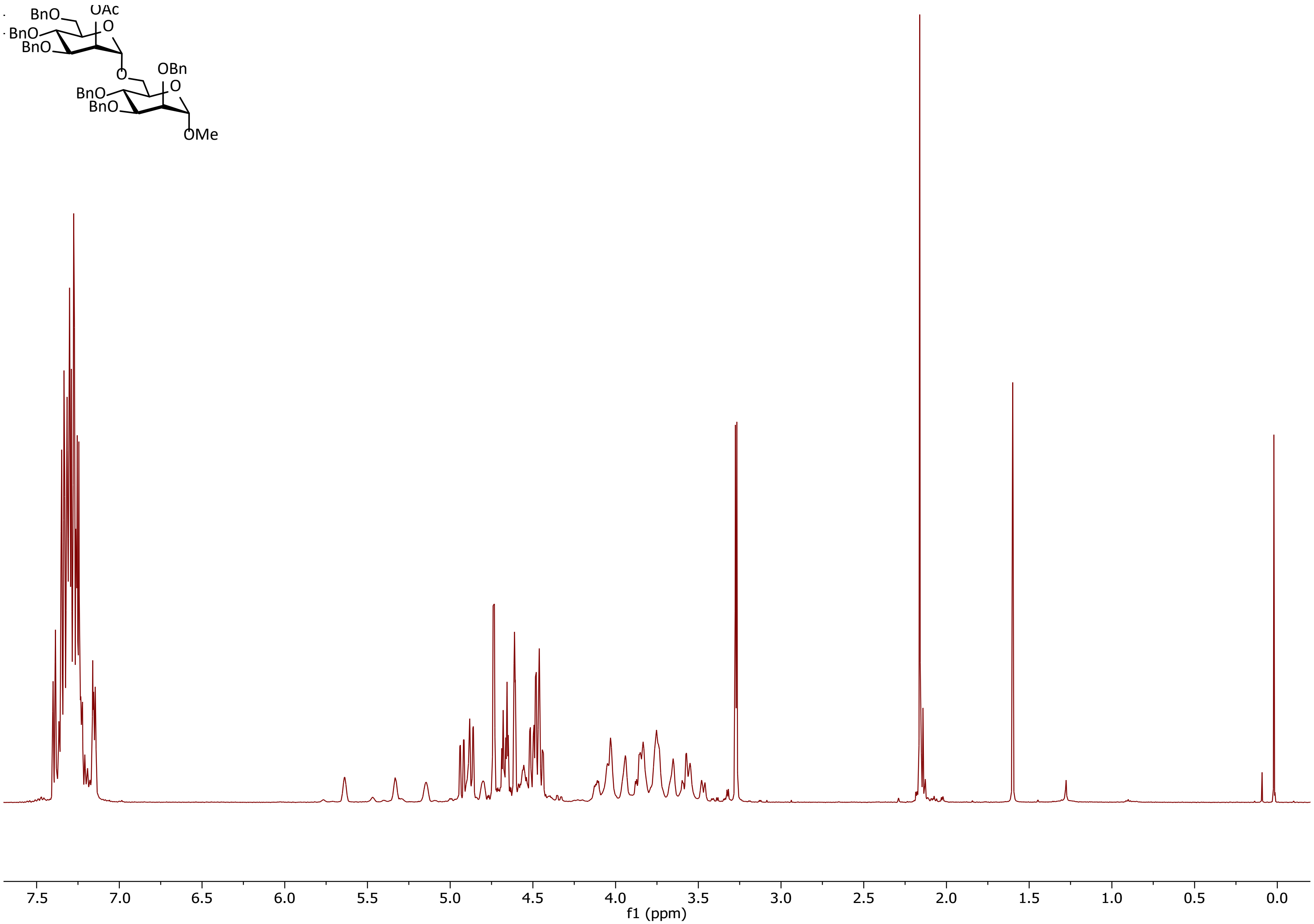

^{13}C NMR spectrum of Methyl 2-*O*-acetyl-3,4,6-tri-*O*-benzyl-α-D-[$^{13}C_6$]mannopyranosyl-(1→6)-2,3,4-tri-*O*-benzyl-α-D-[$^{13}C_6$]mannopyranoside (**8**).

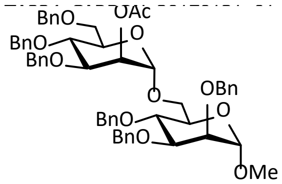

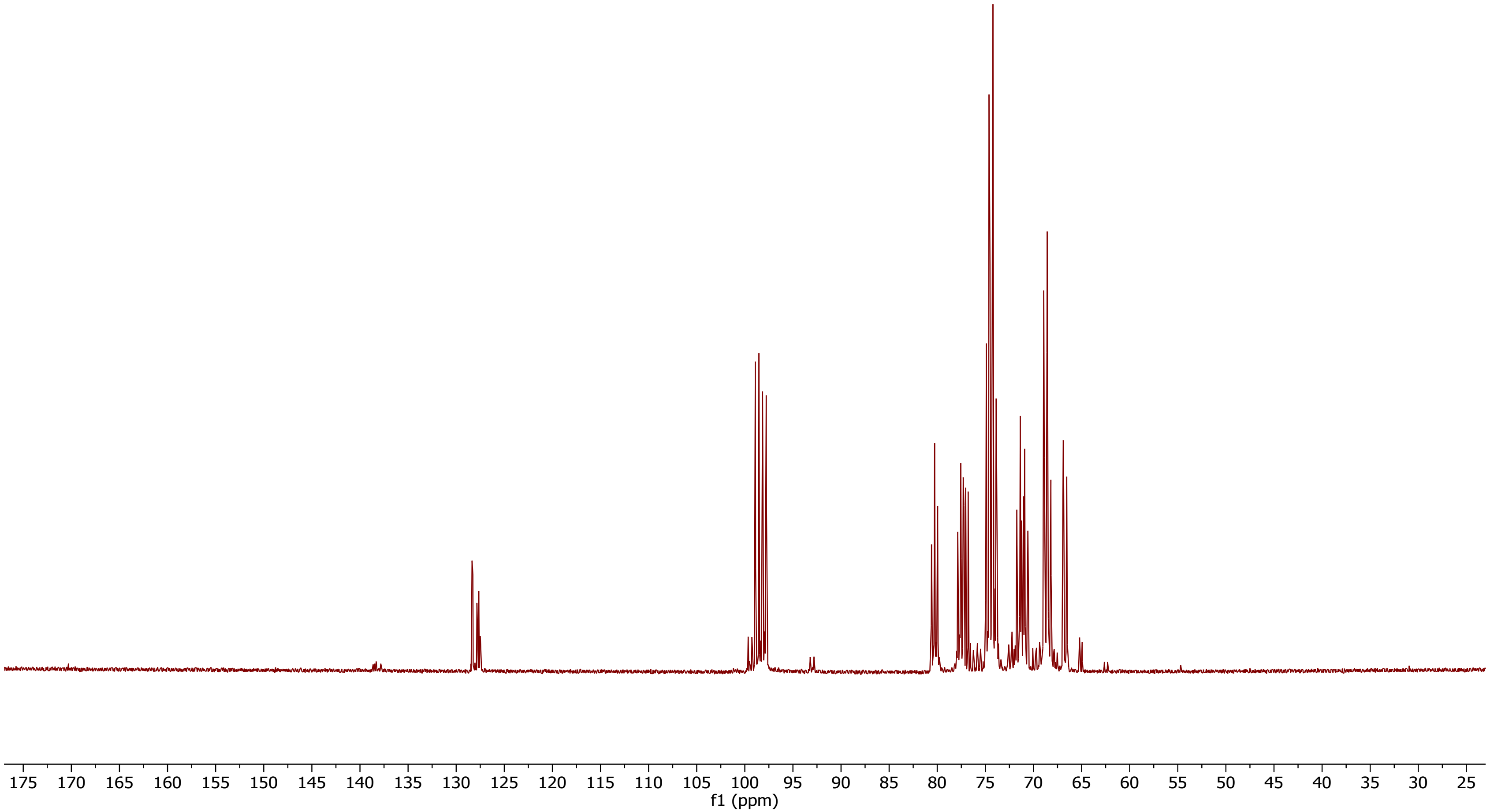

Coupled HSQC spectrum of Methyl 2-*O*-acetyl-3,4,6-tri-*O*-benzyl-α-D-[$^{13}C_6$]mannopyranosyl-(1→6)-2,3,4-tri-*O*-benzyl-α-D-[$^{13}C_6$]mannopyranoside (8).

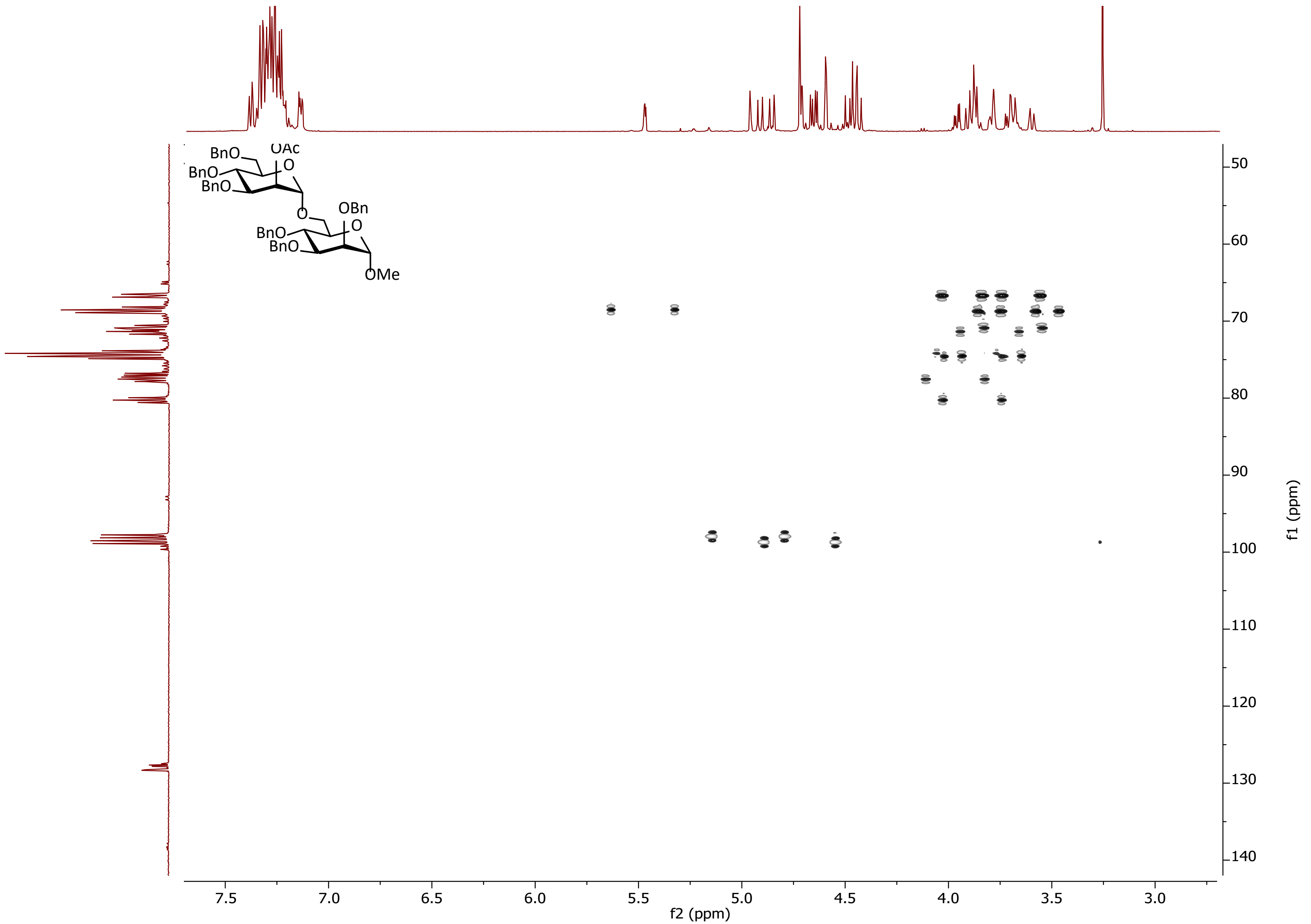

^{1}H NMR spectrum of Methyl 3,4,6-tri-*O*-benzyl-α-D-mannopyranosyl-(1→6)-2,3,4-tri-*O*-benzyl-α-D-mannopyranoside (9).*

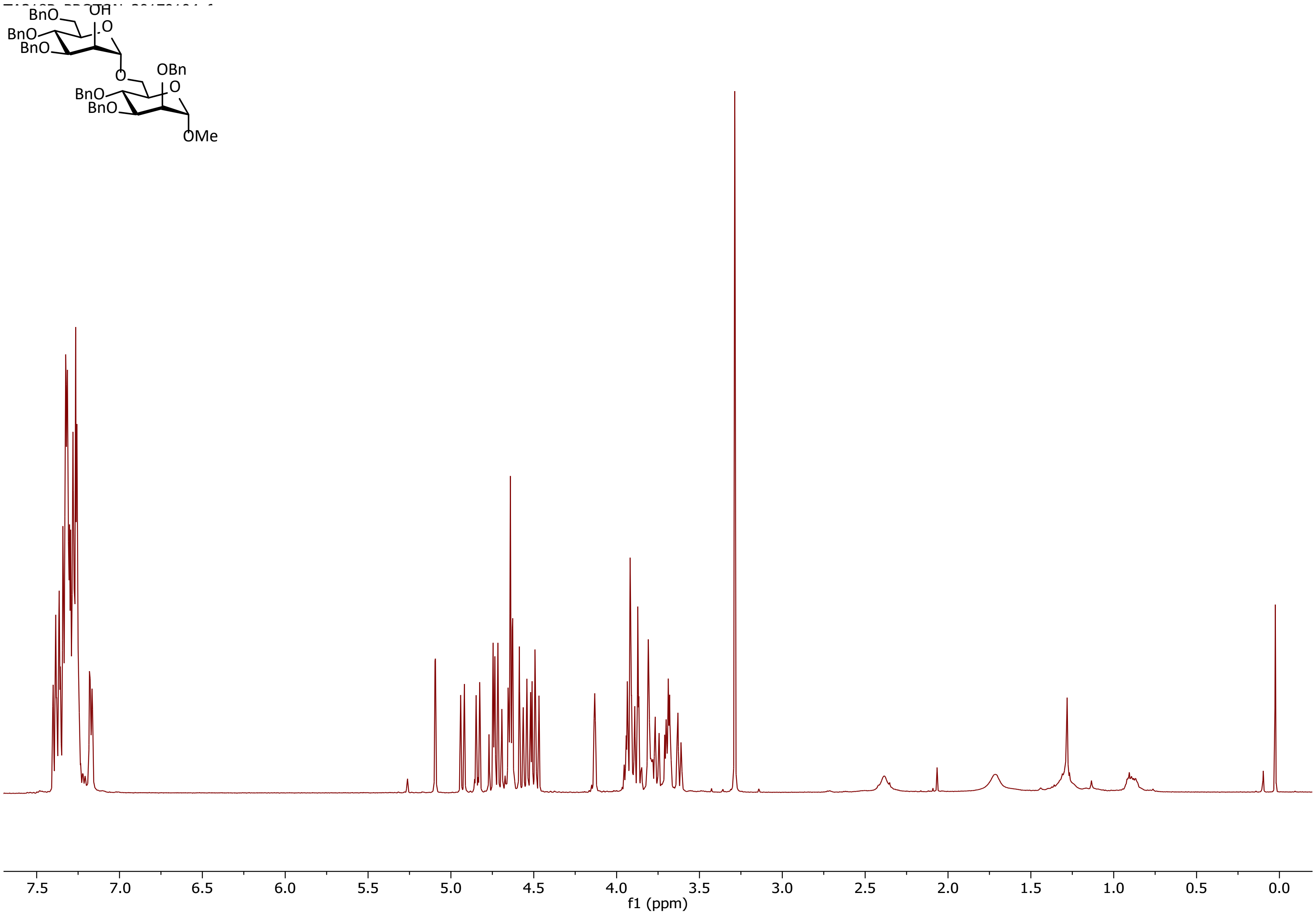

^{1}H NMR spectrum of Methyl 3,4,6-tri-*O*-benzyl-α-D-[$^{13}C_6$]mannopyranosyl-(1→6)-2,3,4-tri-*O*-benzyl-α-D-[$^{13}C_6$]mannopyranoside (**9**).

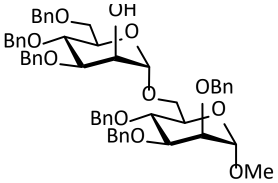

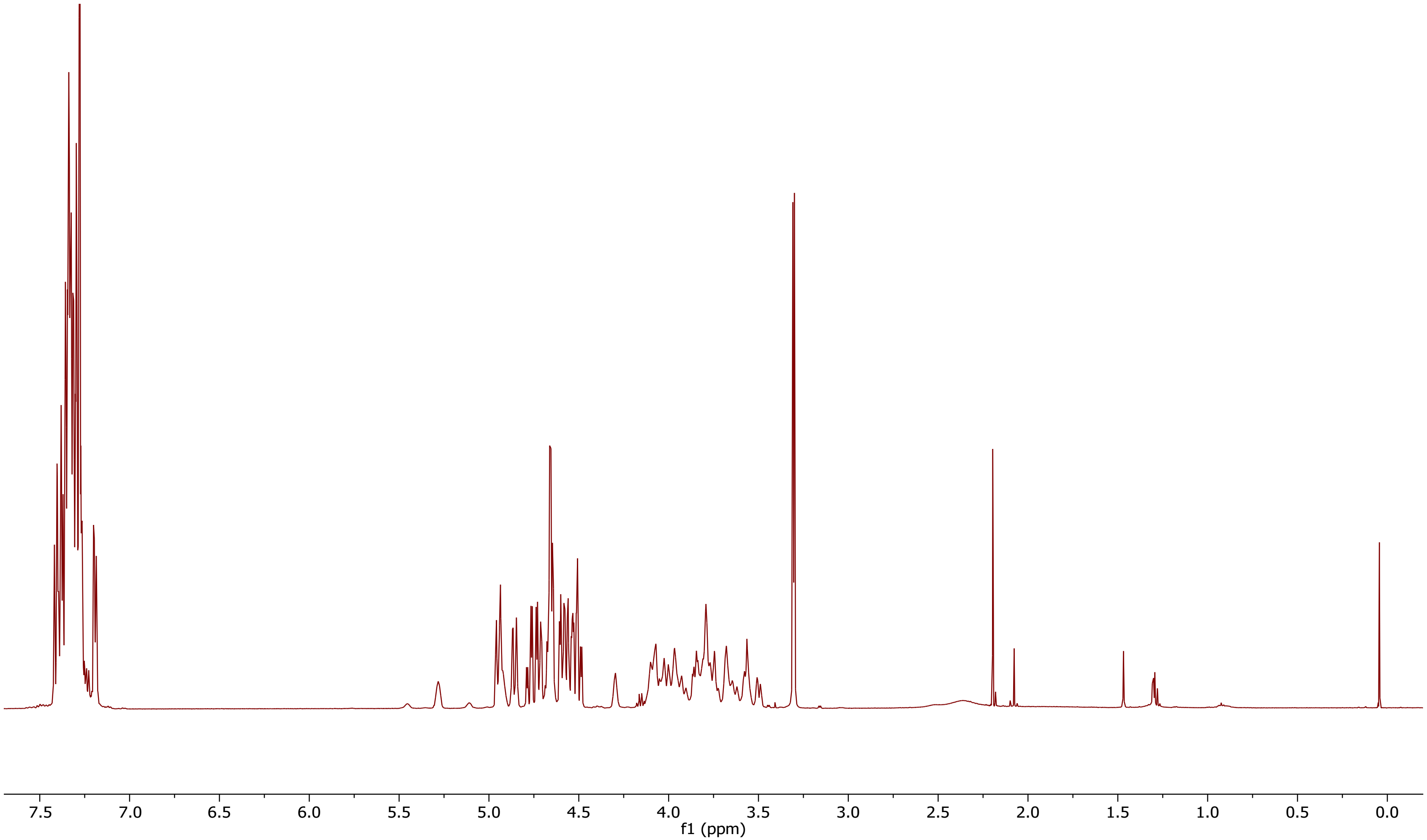

^{13}C NMR spectrum of Methyl 3,4,6-tri-*O*-benzyl-α-D-[$^{13}C_6$]mannopyranosyl-(1→6)-2,3,4-tri-*O*-benzyl-α-D-[$^{13}C_6$]mannopyranoside (**9**).

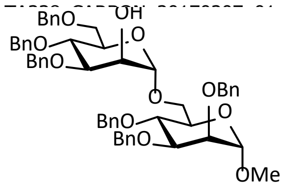

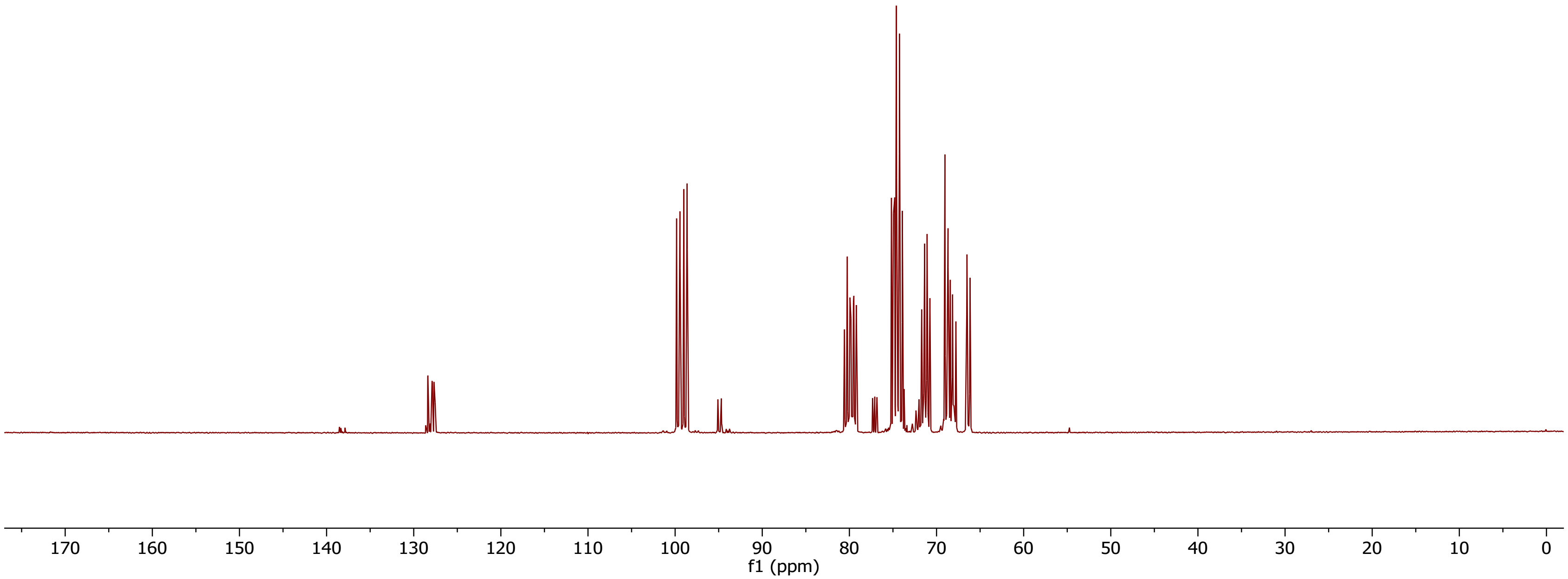

^{1}H NMR spectrum of Methyl 2-O-acetyl-3,4,6-tri-*O*-benzyl-α-D-mannopyranosyl-(1→2)-3,4,6-tri-*O*-benzyl-α-D-mannopyranosyl-(1→6)-2,3,4-tri-*O*-benzyl-α-D-mannopyranoside (**10**).*

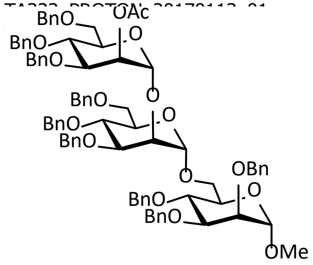

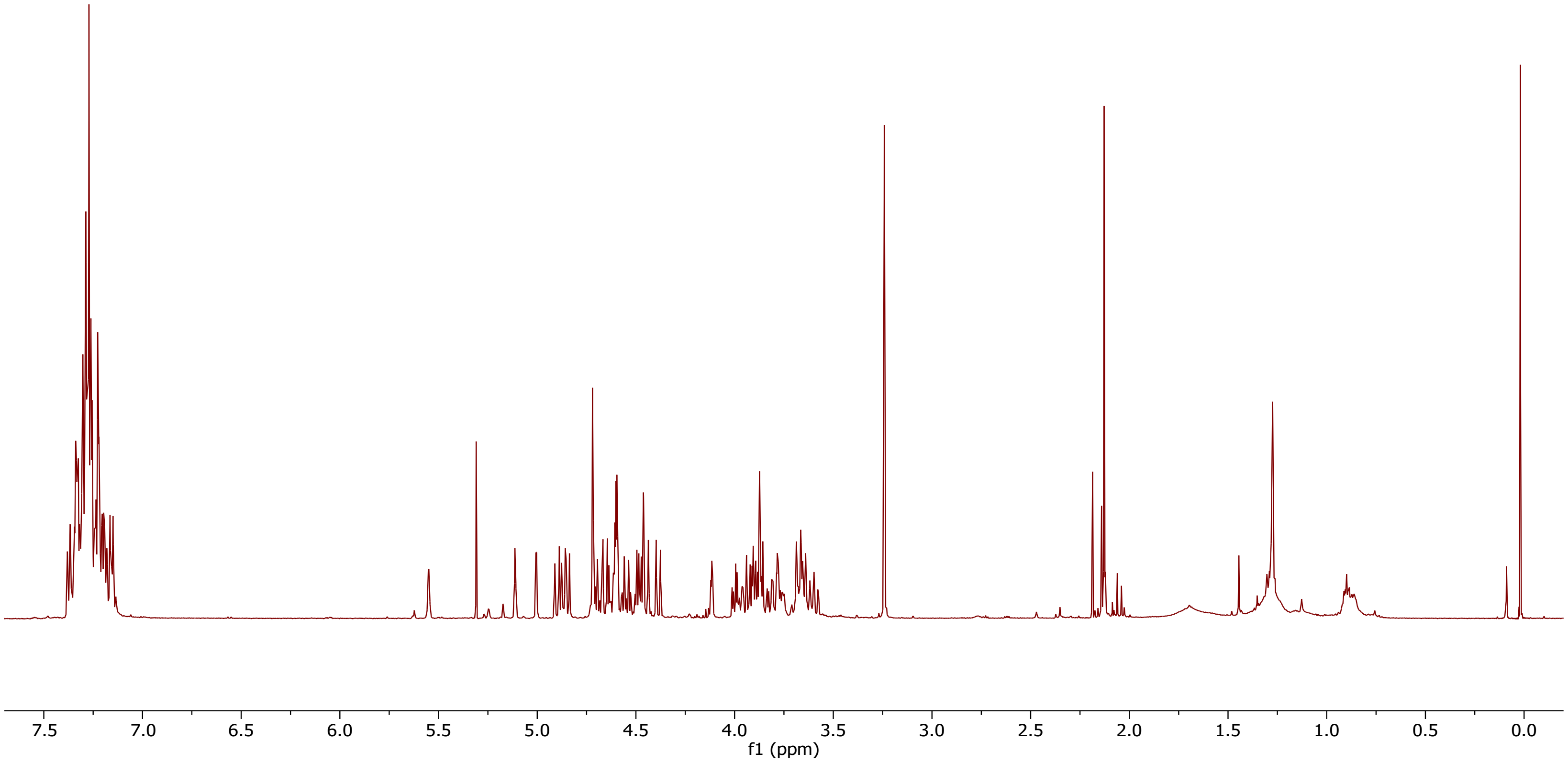

^{1}H NMR spectrum of Methyl 2-O-acetyl-3,4,6-tri-*O*-benzyl-α-D-[[13]C_6]mannopyranosyl-(1→2)-3,4,6-tri-*O*-benzyl-α-D-[[13]C_6]mannopyranosyl-(1→6)-2,3,4-tri-*O*-benzyl-α-D-[[13]C_6]mannopyranoside (10).

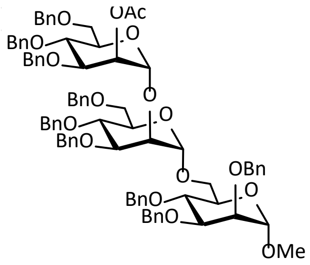

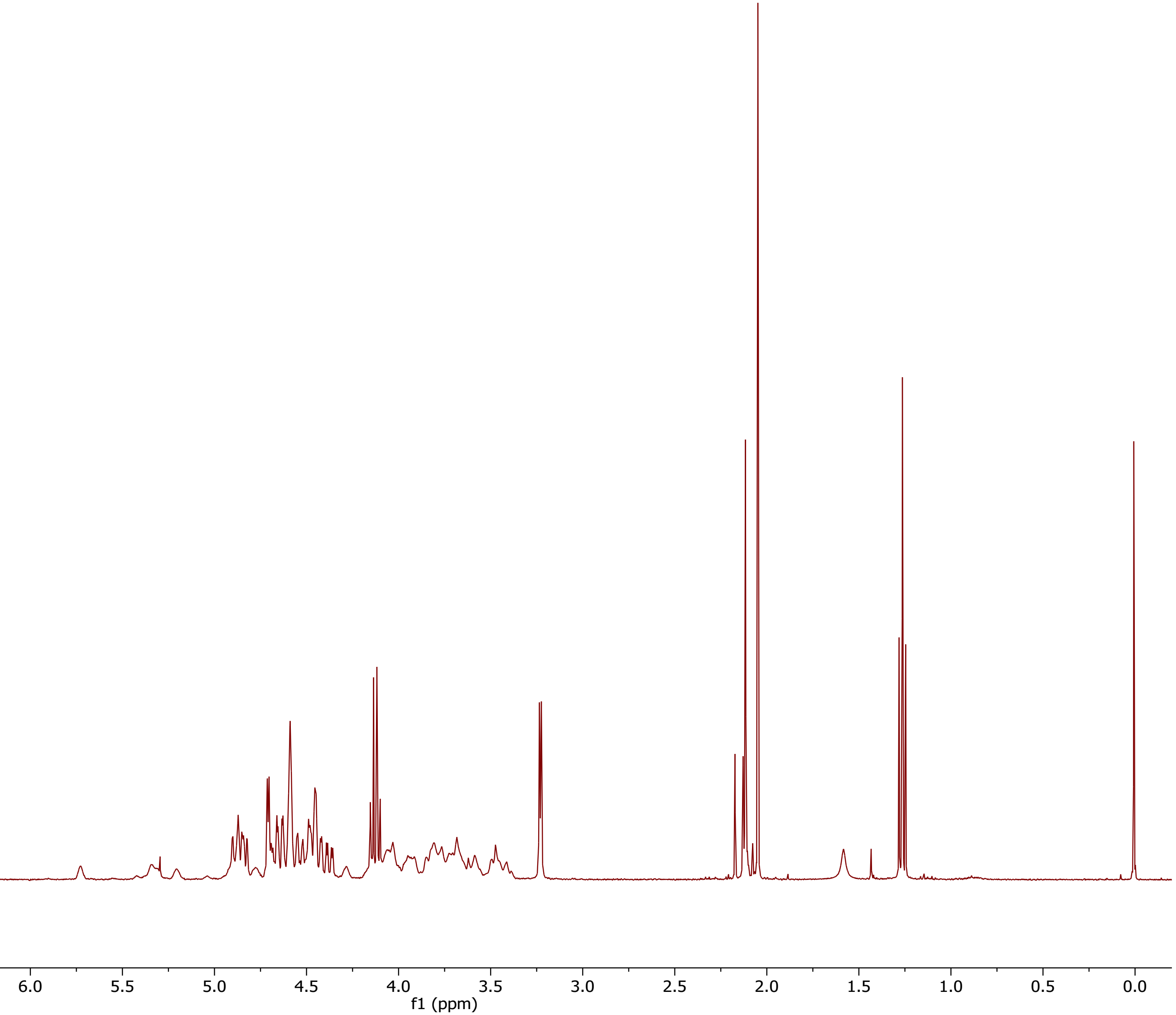

^{13}C NMR spectrum of Methyl 2-O-acetyl-3,4,6-tri-*O*-benzyl-α-D-[$^{13}C_6$]mannopyranosyl-(1→2)-3,4,6-tri-*O*-benzyl-α-D-[$^{13}C_6$]mannopyranosyl-(1→6)-2,3,4-tri-*O*-benzyl-α-D-[$^{13}C_6$]mannopyranoside (**10**).

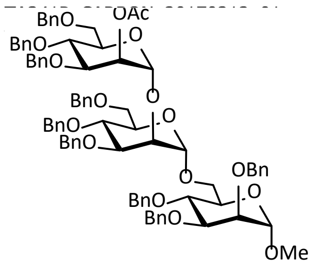

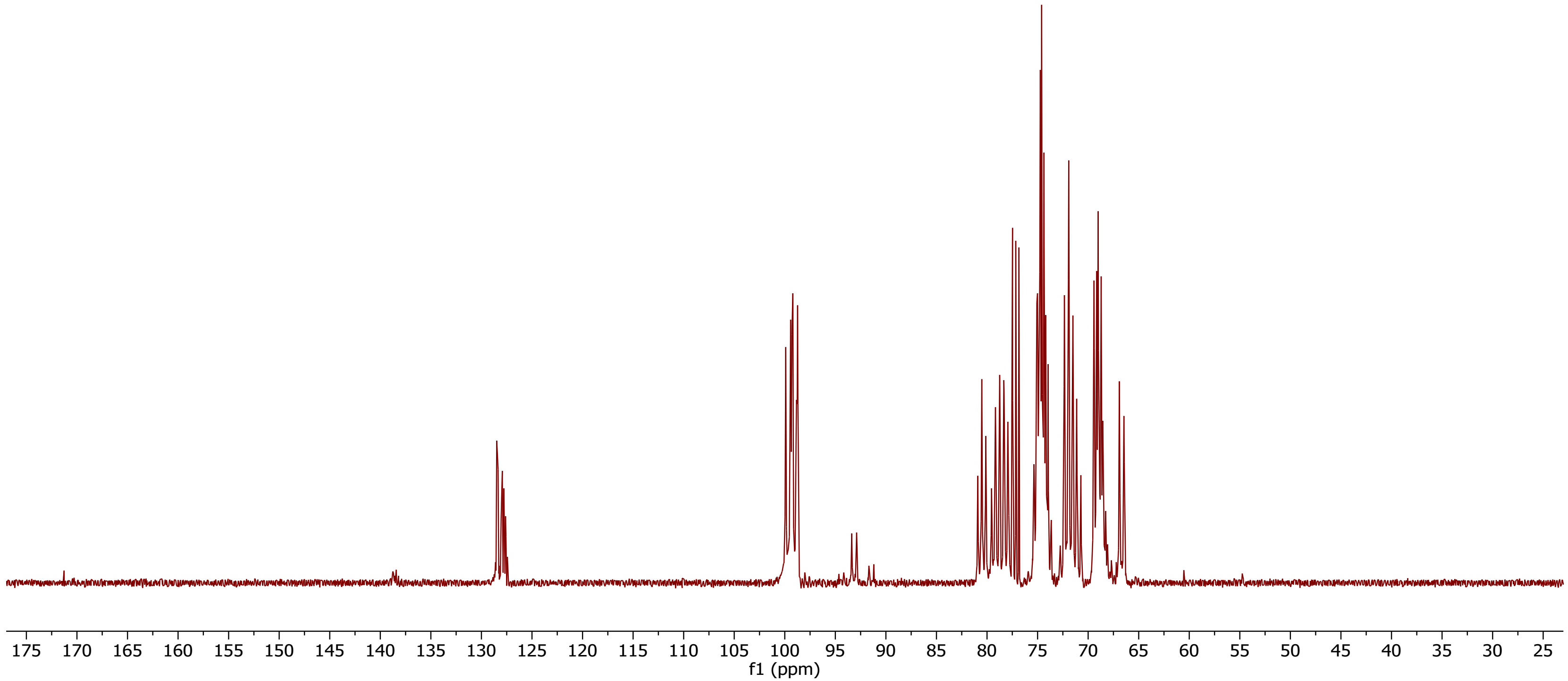

Coupled HSQC spectrum of Methyl 2-O-acetyl-3,4,6-tri-*O*-benzyl-α-D-[$^{13}C_6$]mannopyranosyl-(1→2)-3,4,6-tri-*O*-benzyl-α-D-[$^{13}C_6$]mannopyranosyl-(1→6)-2,3,4-tri-*O*-benzyl-α-D-[$^{13}C_6$]mannopyranoside (10).

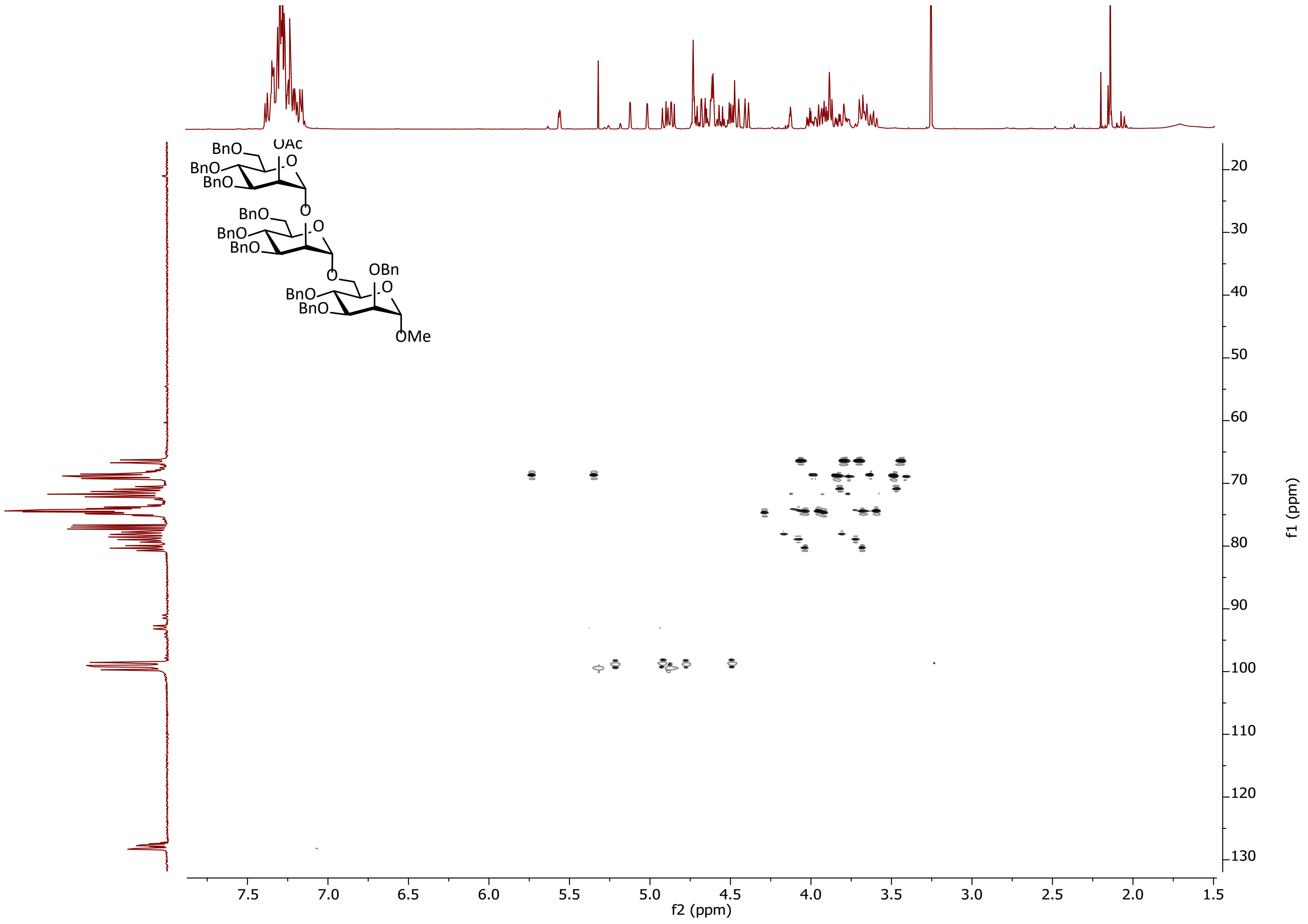

^{1}H NMR spectrum of Methyl *O*-α-D-mannopyranosyl-(1→2)-*O*-α-D-mannopyranosyl-(1→6)-*O*-α-D-mannopyranoside (**11**).*

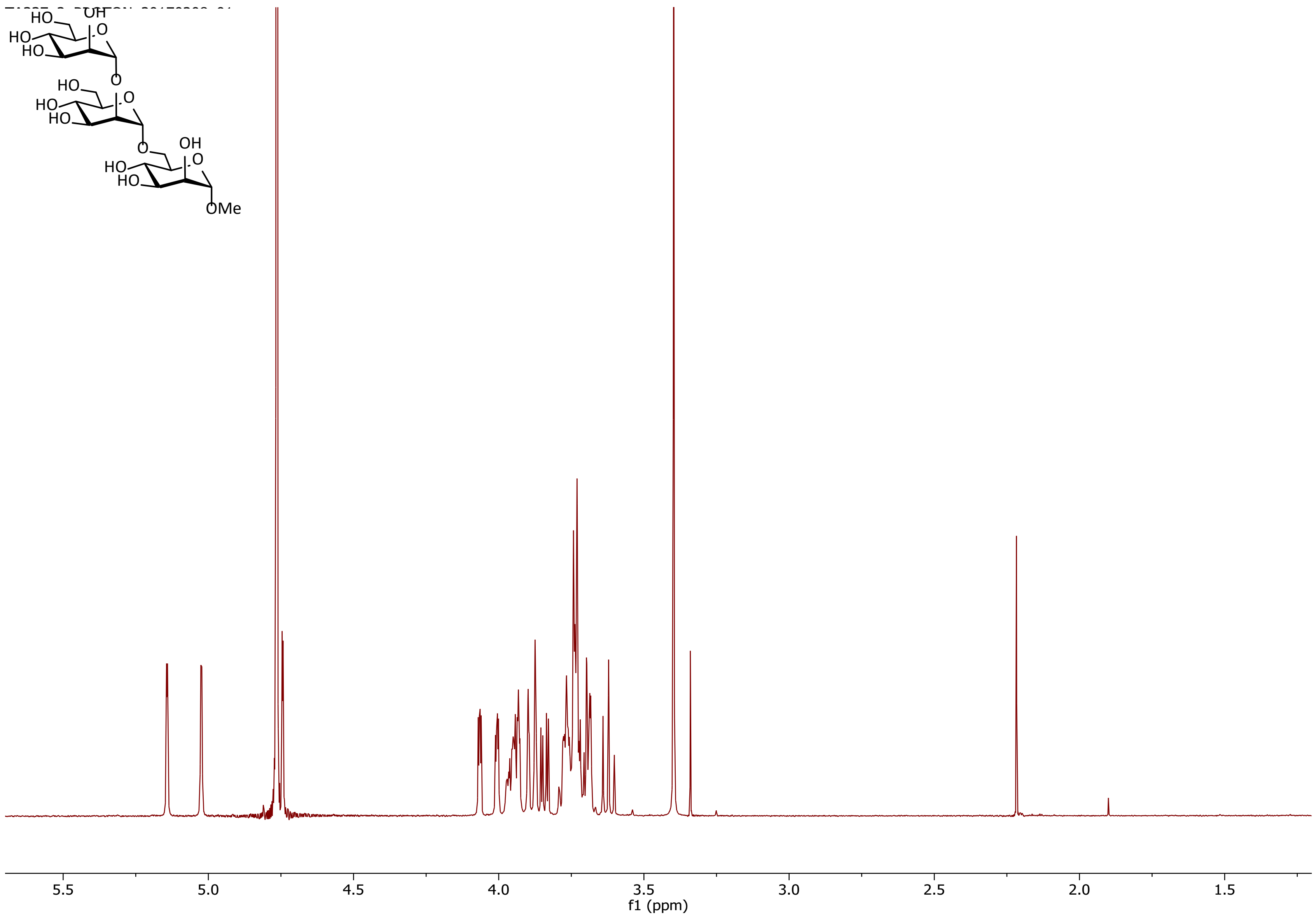

^{1}H NMR spectrum of Methyl *O*-α-D-[$^{13}C_6$]mannopyranosyl-(1→2)-*O*-α-D-[$^{13}C_6$]mannopyranosyl-(1→6)-*O*-α-D-[$^{13}C_6$]mannopyranoside (11).

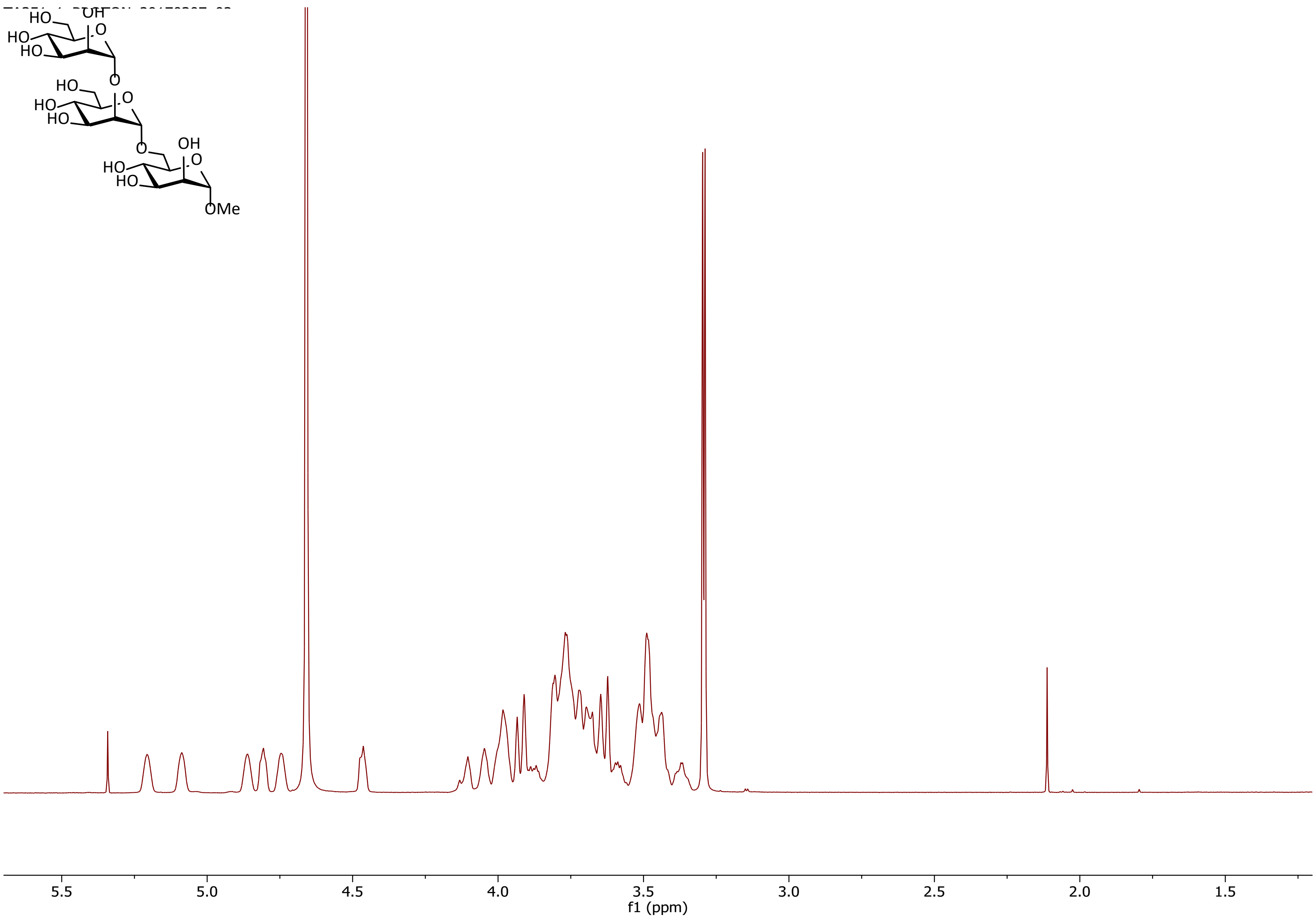

^{13}C NMR spectrum of Methyl *O*-α-D-[$^{13}C_6$]mannopyranosyl-(1→2)-*O*-α-D-[$^{13}C_6$]mannopyranosyl-(1→6)-*O*-α-D-[$^{13}C_6$]mannopyranoside (11).

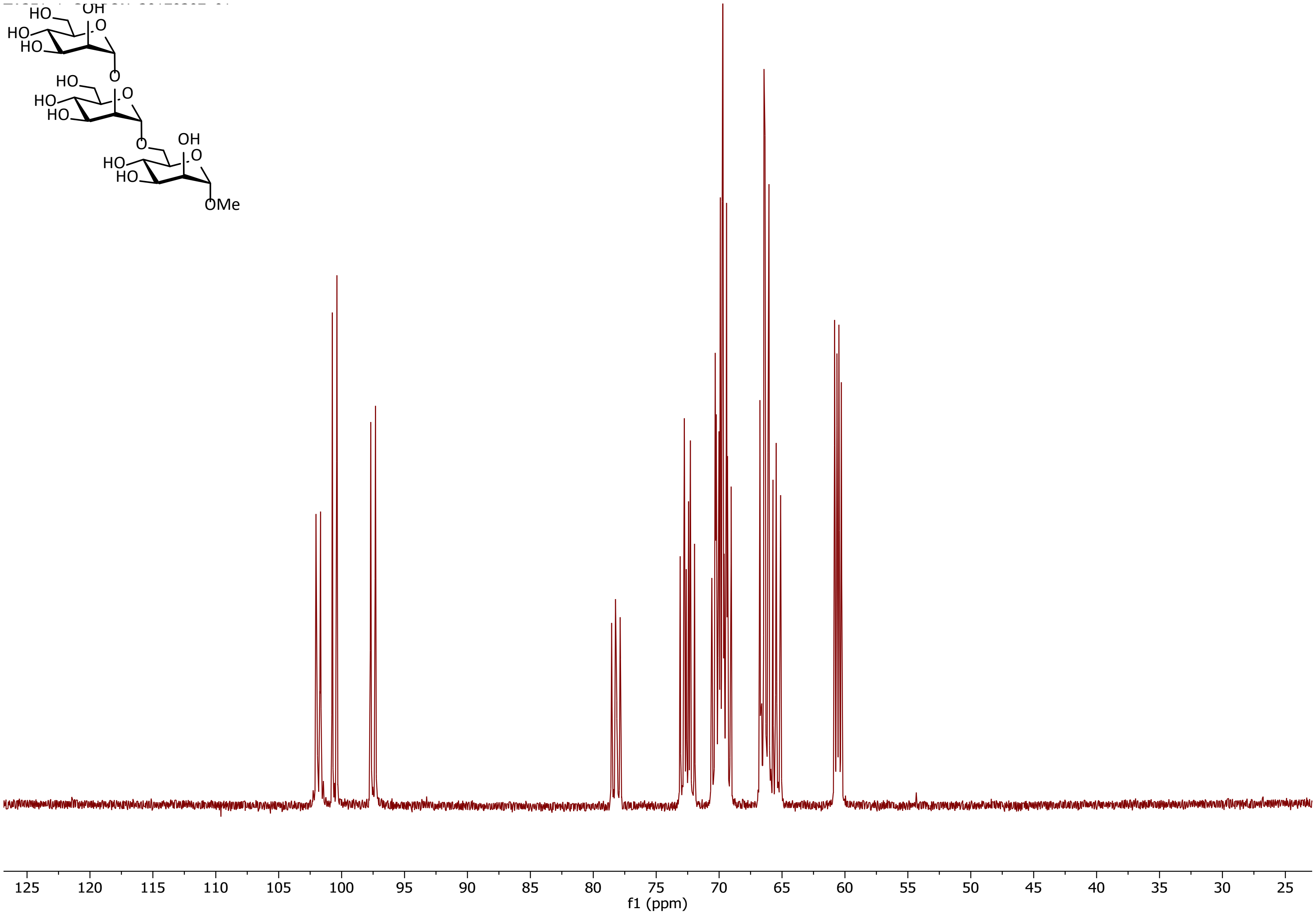

Supplement: Supplementary_data_cwaa081 [file supplementary_data_cwaa081.pdf]
